# Cell-free DNA size deconvolution resolves nucleosomal origins and reveals tumor-associated fragmentomic alterations

**DOI:** 10.1038/s41467-026-72925-4

**Published:** 2026-05-08

**Authors:** Ze Zhou, Wendy N. Cooper, Zhao Cheng, Sara Lightowlers, Charlotte E. Coles, Amit Roshan, Nitzan Rosenfeld, Hui Zhao

**Affiliations:** 1 Centre for Cancer Cell and Molecular Biology, Barts Cancer Institute, Queen Mary University of London, London, UK; 2 Cancer Research UK Cambridge Institute, University of Cambridge, Li Ka Shing Centre, Cambridge, UK; 3 Cancer Research UK Cambridge Centre, Cancer Research UK Cambridge Institute, Li Ka Shing Centre, Cambridge, UK; 4 Department of Oncology, University of Cambridge School of Clinical Medicine, Cambridge, UK; 5 Department of Plastic & Reconstructive Surgery, Cambridge University Hospitals NHS Foundation Trust, Cambridge, UK

**Keywords:** Diagnostic markers, Tumour biomarkers

## Abstract

Analysis of cell-free DNA (cfDNA) fragmentomic features holds great promise for minimally invasive cancer diagnostics. Although selectively analyzing short plasma cfDNA enriches tumor-derived DNA (ctDNA), the mechanisms shaping cfDNA size profiles remain incompletely understood. Here, we develop a generalized model of cfDNA fragment length distributions across multiple bodily fluids (saliva, urine, cerebrospinal fluid, lymphatic fluid, and plasma), deconvoluting size profiles into ~10-bp periodic peaks (components), each approximated by a Cauchy–Lorentz distribution. This analytical framework enables investigation of cfDNA fragmentation across diverse pathological states and reveals a 159-bp component that may demarcate intra- and inter-nucleosomal cfDNA. By analyzing plasma DNA from individuals harboring germline *TP53* mutations, patients receiving radiotherapy, and liver transplantation recipients, we demonstrate that ctDNA shortening can be distinguished from phagocytosis-associated cfDNA shortening through differences in the amplitude and scale parameters of intra- and inter-nucleosomal components. Moreover, leveraging tumor-related fragmentomic alterations, characterized by increased fragmentation entropy identified through cfDNA size deconvolution, significantly enhances cancer detection.

## Abstract (web summary)

cfDNA size is a key feature in fragmentomics for non-invasive cancer detection. Here, the authors present a deconvolution analysis of cfDNA size profiles using the Lorentzian distribution, revealing insights into ctDNA fragmentomic alterations.

## Introduction

Cell-free DNA (cfDNA) molecules are non-randomly fragmented^1^. Recently, increasing attention has been given to the fragmentation patterns of cfDNA molecules, referred to as fragmentomics^1,2^. Among cfDNA fragmentomic features, fragment length is one of the most comprehensively studied characteristics^3–5^, showing great potential for detecting multiple cancer types, including at early stages^6,7^. The vast majority of cfDNA fragments are shorter than 250 bp^1,4,5^, reflecting DNA packaging within mononucleosomes or bound to transcription factors^2,8,9^. Plasma cfDNA typically has a modal fragment length of ~167 bp (the major peak)^8^, with a series of 10-bp periodic minor peaks observed in shorter fragments (< 150 bp)^5^. These minor peaks are thought to result from histones shielding the DNA from nuclease digestion^8,10,11^. In this structure, ~147 bp of DNA is thought to be wrapped around a histone octamer core, with an additional ~20 bp stabilized by the linker histone H1^2,12^. cfDNA molecules in other bodily fluids exhibit diverse size profiles. In particular, those from cerebrospinal fluid (CSF)^13^, urine^14^, and saliva^15^ are more fragmented than plasma cfDNA, enriching for short DNA molecules and exhibiting more pronounced 10-bp periodic oscillations.

In the plasma of cancer patients, circulating tumor-derived DNA (ctDNA) molecules generally exhibit shorter fragment lengths than background normal cfDNA, which is primarily of hematopoietic origin^1^. This size shortening pattern has been consistently observed across multiple cancer types and in various ctDNA manifestations, including DNA molecules carrying tumor mutant alleles^16^, genomic regions with tumor-associated copy number gains^4^, as well as xenografted human tumor DNA in mouse models^16^. The size profiles of shortened ctDNA and normal plasma cfDNA intersect at ~150 bp^1^. Accordingly, plasma DNA molecules below 150 bp are commonly classified as short fragments. Mouliere et al. demonstrated that in silico selection of these short plasma cfDNA fragments (< 150 bp) could enhance ctDNA detection by enriching tumor-derived ctDNA molecules^16^.

cfDNA fragment length features have also demonstrated potential for cancer detection^6,7^. The proportions of cfDNA fragments within specific size ranges have frequently been used to characterize the cfDNA size distribution. Each proportion, determined by the area under the curve within that range, represents an integral over the specified size interval. For instance, Mouliere et al. developed machine learning methods for cancer detection that utilized the proportions of fragments in multiple size ranges (e.g., 20–150, 100–150, 160–180, 180–220, and 250–320 bp) and the ratios between these proportions^16^. Cristiano et al. developed DELFI (DNA evaluation of fragments for early interception), which employs the ratio of short (100–150 bp) to long (151–220 bp) cfDNA molecules within 5 Mb bins across the whole genome for cancer detection^17^. Zhang et al. extended this approach to short (65–150 bp), intermediate (151–220 bp), and long (221–400 bp) fragment ratios within 5 Mb bins, and further characterized chromosome arm-level cfDNA size profiles by analyzing multiple 5-bp size ranges from 65 bp to 400 bp^18^.

Beyond the size range, intensive efforts have been devoted to harnessing cfDNA size profiles for cancer detection. For example, Renaud et al. applied the non-negative matrix factorization (NMF) algorithm to extract ctDNA fragment length signatures and constructed a representative NMF component for cancer detection^19^. Furthermore, Mouliere et al. incorporated the extent of the 10 bp periodic oscillations in cfDNA size profile, measured as the peak-to-trough height difference in sub-nucleosomal fragments, as a feature in machine learning models to enhance cancer detection^16^. Recently, Carey et al. demonstrated the feasibility of approximating the overall cfDNA fragment length distribution using a Bayesian mixture model comprising 12 truncated Gaussian (normal) distributions^20^. Building upon this work, van’t Erve et al. further developed DELFI-TF, a random forest regression–based approach for quantifying ctDNA fraction using cfDNA size characteristics. DELFI-TF takes the areas under the truncated Gaussian distributions as input features, which contribute ~32% to the model performance, with the genome-wide short-to-long ratio profile contributing ~13%^21^.

Although numerous methods have been developed to leverage the fragment length features of cfDNA^16–21^, their utility is restricted by a limited understanding of the biological mechanisms of cfDNA degradation and its association with apparent fragmentation patterns. This limitation may lead to the arbitrary selection of fragment sizes that are putatively the most informative in different studies. In addition, cfDNA size profiles can vary due to pre-analytical factors such as storage conditions^22^, DNA library preparation protocols^23^, and sequencing platforms^24^. This variability limits the utility of cfDNA fragment length information and reduces the generalizability of bespoke algorithms across different studies, cohorts, and cancer types. Consequently, there is a risk of overfitting when applying algorithms that rely on confined sections of cfDNA size profiles spanning limited size ranges.

Here, we propose a generalizable size model for cfDNA molecules that deconvolutes fragment length distributions into a series of Cauchy–Lorentz distributions (hereafter referred to as Lorentzian distributions). This model is broadly applicable to cfDNA from various body fluids, tissues of origin, and pathophysiological conditions. Using deconvolution analysis, the model offers a renewed perspective on cfDNA fragment length distributions and introduces an analytical framework for exploring cfDNA size profiles, revealing underlying biological processes involved in cfDNA fragmentation. Importantly, our size model uncovers ctDNA-associated fragmentation signatures and demonstrates its potential to enhance cancer detection by focusing on tumor-derived fragmentomic alterations through decoupling from immune cell–mediated fragmentation signals.

## Results

### Modeling cfDNA size profiles using the Lorentzian distribution

**Fig. 1:**
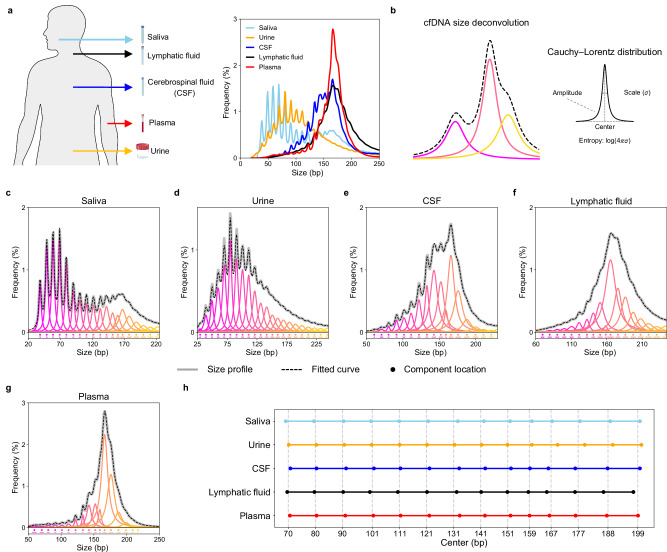
Cell-free DNA (cfDNA) size deconvolution analysis across multiple bodily fluids. **a** Size profiles of pooled cfDNA from saliva (27.1 million fragments from two samples; light blue line), urine (149.9 million fragments from 18 samples; yellow line), cerebrospinal fluid (CSF) (76.6 million fragments from 13 samples; blue line), lymphatic fluid (906.6 million fragments from 28 samples; black line), and plasma samples (497.1 million fragments from 19 samples; red line). **b** Principle of cfDNA size profile deconvolution analysis. Left, a multimodal distribution (dashed line) can be deconvoluted into three Lorentzian distributions (components). Curve height is represented by the sum of the heights of the individual components. Right, a Lorentzian distribution is defined by three parameters: center (location), scale (width), and amplitude (area). The entropy of a Lorentzian distribution is a function of its scale parameter, defined as the natural logarithm of the product of 4$\pi$ and its scale. Size deconvolution analysis results of cfDNA from **c** saliva (19 components), **d** urine (22 components), **e** CSF (18 components), **f** lymphatic fluid (18 components), and **g** plasma (17 components). Each component has three degrees of freedom (center, amplitude and scale). The maximum scale value was set to 9 bp, with no other optimization constraint applied in the size deconvolution analysis. The original size profiles are shown as gray lines, Lorentzian components as colored peaks, and the fitted curves as black dashed lines closely overlapping the gray profiles. Lower panels display the center (dot) and scale (horizontal line) parameters of each component. **h** Distribution of component center parameters of cfDNA from saliva, urine, CSF, lymphatic fluid, and plasma. Source data are provided as a Source Data file.

As shown in Fig. 1a, cfDNA fragment length distributions vary across bodily fluids. The median lengths decrease from lymphatic fluid (171 bp) to plasma (167 bp), CSF (154 bp), saliva (106 bp) and urine (104 bp). We hypothesized that, despite the diverse fragment length distributions, cfDNA molecules from different bodily fluids share intrinsic properties shaped primarily by nucleosome structures. Based on this assumption, we developed a unified model of cfDNA fragment length distributions. This model employs a series of Lorentzian distributions with a ~10 bp periodicity to capture the characteristic oscillatory peaks associated with the nucleosomal 10 bp footprint. To implement this, we developed a cfDNA size deconvolution analysis, in which size profiles are decomposed into multiple overlapping peaks (referred to as components). Each component is defined by three parameters: center (location), amplitude (area), and scale (width) (Fig. 1b; “Methods”).

We applied size deconvolution analysis to cfDNA from saliva (27.1 million fragments; Fig. 1c), urine (149.9 million fragments; Fig. 1d), CSF (76.6 million fragments; Fig. 1e), lymphatic fluid (906.6 million fragments; Fig. 1f), and plasma (497.1 million fragments; Fig. 1g). Remarkably, the size profiles of cfDNA from all five bodily fluids could be deconvoluted into multiple Lorentzian components that are regularly distributed across the entire size range and exhibit progressively changing amplitudes. Of note, the biochemical environments of bodily fluids differ in factors such as pH, ionic composition, and salt levels. In addition, DNA nuclease activities vary considerably among bodily fluids, e.g., DNASE1L3 shows the highest activity in plasma, while DNASE1 displays predominant activity in urine^10,25^. Given these differences, cfDNA fragment size profiles from all five types of bodily fluids were modeled exclusively using Lorentzian distributions, substantiating our hypothesis.

We also tested whether the cfDNA size profiles could be modeled by other mathematical distributions, such as the Gaussian distribution^20^ and the Student’s *t*-distribution. As shown in Supplementary Fig. S1, in the same parameter settings, the Lorentzian distribution was uniquely able to yield components with regular periodicity and gradually changing amplitudes and scales. More importantly, the Lorentzian model produced the highest coefficients of determination in all five bodily fluids (all *R*^2^ > 0.99), accompanied by the lowest absolute fitting residuals (Supplementary Fig. S2), outperforming both the Gaussian and Student’s *t* distributions. These results confirm that the Lorentzian distribution provides the most accurate and reliable approximation of cfDNA fragment length distributions.

cfDNA molecules in various bodily fluids are derived from diverse tissues, e.g., plasma cfDNA is primarily of hematopoietic origin^26^, urinary cfDNA is predominantly derived from the urinary tract^27^, CSF cfDNA is mostly derived from neuronal tissue^28^, and salivary cfDNA mainly originates from the oral cavity^29^. The feasibility of applying our size deconvolution analysis to cfDNA from these bodily fluids suggests that our cfDNA size model may be applicable to cfDNA derived from all tissue types in bodily fluids under homeostatic conditions.

Previous xenograft studies have shown that human cfDNA in mouse plasma exhibits a more pronounced 10-bp oscillation and consists of multiple consecutive paired peaks with similar widths^16,30,31^, as also illustrated in Supplementary Fig. S3a. To further investigate cfDNA fragmentation patterns, we applied size deconvolution analysis to xenografted human cfDNA. As shown in Supplementary Fig. S3b, c, the components centered at paired locations (such as ~60 and ~70 bp; ~80 and ~90 bp; ~101 and ~111 bp; ~121 and ~131 bp; as well as ~141 and ~151 bp) showed close scale values. These paired-scale components indicate a stepwise fragmentation process of sub-nucleosomal cfDNA, further demonstrating that the deconvolution analysis can reveal more detailed insights into cfDNA size profiles.

### 159 bp demarcates between intra- and inter-nucleosomal cfDNA

**Fig. 2:**
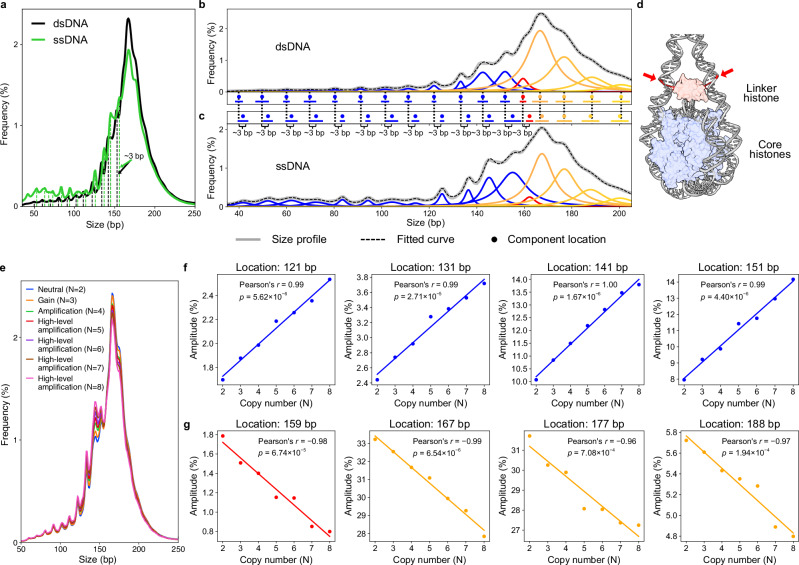
Size deconvolution analysis of single-stranded cfDNA, double-stranded cfDNA, and cfDNA from genomic regions with varying copy-numbers. **a** Size profiles of double-stranded cfDNA (dsDNA; 16.9 million fragments; black line) and single-stranded cfDNA (ssDNA; 27.9 million fragments; green line). Vertical dashed lines indicate the apparent x-axis positions of minor peaks. Size deconvolution analysis of **b** dsDNA and **c** ssDNA. Deconvoluted components below 159 bp were colored blue, the component at ~159 bp was colored red, and those above 159 bp were colored yellow. The original size profiles are shown as gray lines, and the fitted curves are shown as black dashed lines. **d** Illustration of a nucleosome with DNA (gray) wrapped around core histones (light blue) and bound to linker histone H1 (light red) (PDB: 5NL0). The red arrows indicate two ends of a stretch of DNA spanning 159 bp, centered at the nucleosome dyad. **e** Size profiles of cfDNA molecules from different genomic regions, including copy-number neutral (*N* = 2; 48.7 million fragments), tumor-associated gain (*N* = 3; 46.9 million fragments), amplification (*N* = 4; 7.3 million fragments), and high-level amplification regions (*N* = 5 to 8; 9.6, 3.5, 3.9, 2.0 million fragments, respectively). **f**, **g** Changes in amplitude parameters of Lorentzian components at ~121 bp to ~151 bp (blue), ~159 bp (red), and ~167 bp to ~188 bp (yellow) across different tumor copy number statuses (*N* = 2 to 8). Correlations were evaluated using Pearson’s *r* and a two-sided Student’s *t* test without adjustments. Source data are provided as a Source Data file.

As shown in Fig. 1h, the centers of all deconvoluted components appear at ~10 bp intervals, except for the component located at ~159 bp, which is ~8 bp downstream of the preceding 151-bp component and ~8 bp upstream of the following 167-bp component. To determine whether the component at ~159 bp reflects a biologically meaningful nucleosomal structure or is merely a mathematical artifact, we applied our deconvolution method to plasma cfDNA subjected to a single-stranded library preparation protocol (ssDNA)^32^. Both double-stranded cfDNA (dsDNA) and ssDNA molecules exhibit a major peak at ~167 bp^8,32^. Additionally, ssDNA has been reported to show ~3 bp offsets in the minor peaks below ~150 bp^8^, which were also illustrated in Fig. 2a. As shown in Fig. 2b, c, cfDNA size deconvolution analysis provided holistic views of fragment length distributions for both dsDNA (16.9 million fragments) and ssDNA (27.9 million fragments). This analysis revealed a 159-bp component between the minor peaks and the major peak, and more components above the 167-bp major peak at ~177, ~188, and ~199 bp. In ssDNA (Fig. 2c), all components at and below ~159 bp exhibited ~3 bp rightward offsets compared with their counterparts in dsDNA (Fig. 2b), suggesting a shared mechanism of nucleosomal protection against enzymatic nuclease digestion in short cfDNA fragments. By contrast, all components above ~159 bp (at ~167, ~177, ~188 and ~199 bp) showed no offset between ssDNA and dsDNA (Fig. 2b, c), indicating distinct fragmentation mechanisms between long (> 159 bp) and short (≤ 159 bp) cfDNA. Together, these results suggest that the component centered at ~159 bp may be associated with a biologically meaningful nucleosomal structure.

cfDNA fragments shorter than 150 bp, exhibiting a ~10 bp periodicity, are thought to mainly originate from nucleosomal core particle^9^. Therefore, we postulate that the 159-bp fragment length might consist of a single base pair at the nucleosome dyad flanked by two 79-bp stretches of DNA (1 + 79 + 79 bp). As illustrated by the three-dimensional structure in Fig. 2d, both ends of the 159-bp DNA segment complexed with nucleosome are tightly bound to linker histone H1^12^. This aligns with our observation that the components below ~159 bp show a ~3 bp offset between ssDNA and dsDNA, potentially reflecting protection conferred by the nucleosome structures against nuclease degradation. Furthermore, the lower amplitude of the 159-bp component compared to the preceding components (e.g., at ~151 bp and ~141 bp) may indicate a weaker protective effect against nuclease digestion conferred by linker histone H1 binding than by core histones wrapping (Fig. 2d).

To further explore the biology of the 159-bp component, we assessed its roles in ctDNA by analyzing plasma cfDNA from a patient with stage IV breast cancer^33^. Since ctDNA is expected to be enriched in genomic regions with tumor-associated copy number gains^4^, we compared cfDNA molecules from copy-number neutral regions (*N* = 2; length: 677.0 Mb; 48.7 million fragments), with those from regions with tumor-associated copy number gain (*N* = 3; length: 673.0 Mb; 46.9 million fragments), amplification (*N* = 4; length: 90.5 Mb; 7.3 million fragments), and high-level amplifications (*N* = 5, 6, 7, and 8; lengths: 115.5, 41.0, 44.5, and 22.0 Mb; fragment counts: 9.6, 3.5, 3.9, and 2.0 million, respectively). As shown in Fig. 2e, cfDNA fragments derived from genomic regions with higher levels of copy number gains exhibit a progressive increase in short fragments, reflecting increased ctDNA fractions^16^. We next performed size deconvolution analysis on cfDNA fragments from regions with varying tumor copy numbers and examined how the component parameters changed with respect to increasing ctDNA fraction. In agreement with the enrichment of short DNA fragments in ctDNA^1,2^, the amplitudes of components at ~121, ~131, ~141, and ~151 bp showed significant positive correlations with tumor copy numbers (Pearson’s *r* ≥ 0.99, *p* < 0.0001; Fig. 2f). Strikingly, component amplitudes showed opposing trends demarcated by the 159-bp component. In contrast, the amplitudes of components at ~159, ~167, ~177, and ~188 bp showed strong negative correlations (Pearson’s *r* ≤ −0.96, *p* < 0.001; Fig. 2g). Bidirectional changes in the scale parameters also highlighted the demarcating role of 159-bp component (Supplementary Fig. S4). These results demonstrate that the characteristic shortening of ctDNA molecules can be captured by the inverse amplitude changes below and above the 159-bp component. Together, the analyses comparing ssDNA and dsDNA, as well as ctDNA across a range of concentrations, may reveal 159 bp as a fragment length feature associated with the pivot point between intra- and inter-nucleosomal cfDNA.

### Increased intra-nucleosomal fragmentation entropy as a characteristic of ctDNA

To evaluate whether altered amplitude and scale parameters could be used for cancer detection, we classified deconvoluted components below 159 bp as intra-nucleosomal and those at or above 159 bp as inter-nucleosomal. Given that the entropy of a Lorentzian distribution only depends on its scale (Fig. 1b), we converted the scale parameter to an entropy value. We defined amplitude and entropy ratios between the intra- and inter-nucleosomal components to quantify ctDNA–related changes in fragment length distributions (“Methods”). Briefly, the ratios were calculated as the average amplitude or entropy of the intra-nucleosomal components (< 159 bp) divided by the corresponding average value of the inter-nucleosomal components (≥ 159 bp), representing normalized scores for quantifying overall cfDNA fragmentation status.

**Fig. 3:**
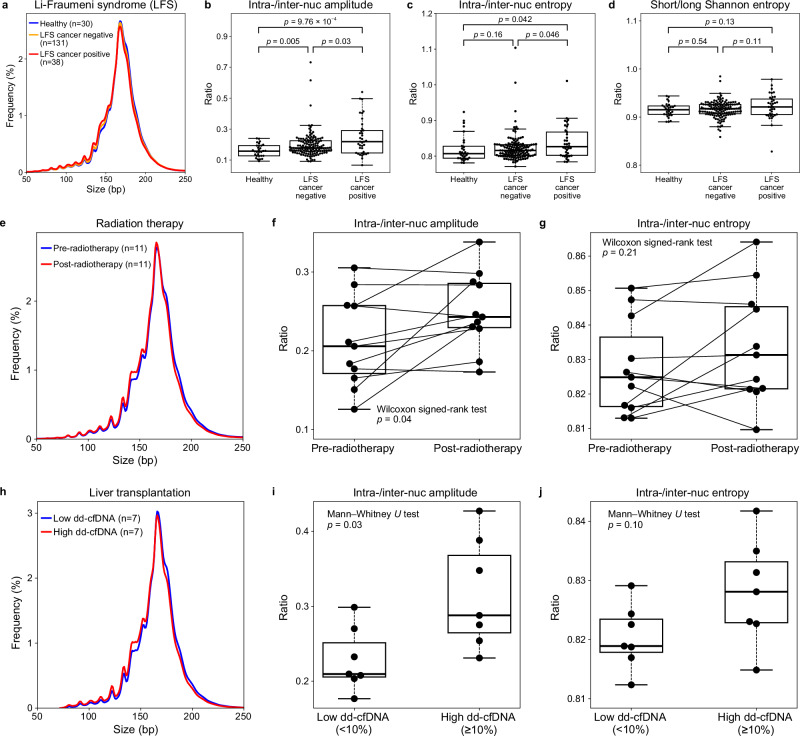
Size deconvolution analysis of plasma cfDNA from patients with Li-Fraumeni syndrome, radiotherapy-treated patients, and liver transplantation recipients. **a** Size profiles of pooled plasma cfDNA of healthy donors (*n* = 30; 1171.3 million fragments; blue line), patients carrying germline *TP53* variants (Li-Fraumeni syndrome, LFS) without cancer (*n* = 131; 3601.0 million fragments; yellow line), and LFS patients with active cancer (*n* = 38; 1019.8 million fragments; red line). Boxplot of intra-/inter-nucleosomal (intra-/inter-nuc) **b** amplitude ratios and **c** entropy ratios in healthy individuals (*n* = 30) and LFS patients with (*n* = 38) and without (*n* = 131) cancer. **d** Boxplot of the Shannon entropy ratios between short (100–150 bp) and long fragments (151–220 bp) in healthy individuals (*n* = 30) and LFS patients with (*n* = 38) and without (*n* = 131) cancer. In each boxplot, the central line represents the median, the box denotes the interquartile range (IQR; 25% to 75% percentiles), and the whiskers extend to the most extreme values within 1.5 times IQR, with all individual data points shown. The statistical tests in (**b**–**d**) were all two-sided Mann–Whitney *U* tests. **e** Size profiles of pooled plasma cfDNA of patients pre- (*n* = 11; 84.9 million fragments; blue line) and post-radiotherapy (*n* = 11; 64.9 million fragments; red line). Boxplot of **f** intra-/inter-nuc amplitude ratios and **g** entropy ratios in patients pre- (*n* = 11) and post-radiotherapy (*n* = 11), with statistical significance assessed by two-sided Wilcoxon signed-rank tests. The central line, box, and whiskers of each boxplot are defined as described above. **h** Size profiles of pooled plasma cfDNA from liver transplantation patients with low donor-derived cfDNA (dd-cfDNA) frequency (< 10%; *n* = 7; 149.0 million fragments; blue line) and high dd-cfDNA frequency (≥ 10%; *n* = 7; 146.2 million fragments; red line). Boxplots of **i** intra-/inter-nucleosomal amplitude ratios and **j** entropy ratios in patients with low (< 10%; *n* = 7) and high (≥ 10%; *n* = 7) level dd-cfDNA, with two-sided Mann–Whitney *U* tests applied. The central line, box, and whiskers of each boxplot are defined as described above. Source data are provided as a Source Data file.

We next assess the intra- and inter-nucleosomal amplitude and entropy changes in plasma of patients with Li-Fraumeni syndrome (LFS). Wong et al. previously showed that plasma cfDNA from pathogenic germline *TP53* mutation carriers (LFS patients) exhibits an enrichment of short DNA molecules resembling those of cancer patients, even in LFS patients without cancer^24^ (also shown in Fig. 3a). To investigate whether cfDNA shortening patterns differ between cancer-negative *TP53*-carriers and cancer patients, we applied size deconvolution analysis to plasma cfDNA of individuals without cancer and with no known genomic alterations in *TP53* (healthy; *n* = 30), individuals with LFS and no known cancer present (LFS patients without cancer; *n* = 131), and individuals with LFS and a cancer diagnosis (LFS patients with cancer; *n* = 38), with a median coverage of 1.6x (range: 0.07 to 3.0x)^24^. As a result, cancer-positive LFS patients exhibited significant increases in both intra- to inter-nucleosomal amplitude ratios (median: 0.22, range: 0.07 to 0.54; *p* = 9.76 × 10^−4^, Mann–Whitney *U *test; Fig. 3b) and entropy ratios (median: 0.83, range: 0.78 to 1.01; *p* = 0.042, Mann–Whitney *U *test; Fig. 3c) compared with healthy individuals (median amplitude ratio: 0.16, range: 0.09 to 0.24; and median entropy ratio: 0.81, range: 0.78 to 0.92), demonstrating that ctDNA molecules exhibit relatively enhanced intra-nucleosomal protection (higher amplitudes) and increased intra-nucleosomal fragmentation entropy (broader scales). It indicates that tumor-derived ctDNA is generated, at least in part, through a fragmentation process distinct from that of normal cfDNA.

Notably, in LFS patients without cancer, although the intra-/inter-nucleosomal amplitude ratio (median: 0.18; range: 0.09 to 0.73) was significantly higher than that of healthy subjects (*p* = 0.005, Mann–Whitney *U *test; Fig. 3b), no significant difference was observed in their intra-/inter-nucleosomal entropy ratio (median: 0.82; range: 0.77 to 1.10; *p* = 0.16, Mann–Whitney *U *test; Fig. 3c). This demonstrates that non-malignant cfDNA shortening does not contribute to an increase in entropy, whereas increased intra-nucleosomal fragmentation entropy is associated with tumor-derived ctDNA.

Previously, Esfahani et al. demonstrated that Shannon entropy of cfDNA fragment length distributions in gene promoter regions correlates with gene expression^34^. In light of this, we examined whether the Shannon entropy of short (100–150 bp) fragments, long (151–220 bp) fragments, or their ratio (short-to-long Shannon entropy ratio), could align with our fragmentation entropy computed from the scale parameter of the Lorentzian component. However, our analysis revealed a noticeable but not statistically significant difference in the Shannon entropy of short fragments (*p* = 0.07, Kruskal–Wallis test), and no significant differences in long fragments (*p* = 0.53, Kruskal–Wallis test) or short-to-long Shannon entropy ratio (*p *= 0.20, Kruskal–Wallis test) among the healthy individuals and the LFS patients with and without cancer (Fig. 3d, Supplementary Fig. S5).

We reasoned that the Shannon entropy measures the randomness of cfDNA fragment length distributions within a given range but does not account for the sequential pattern of fragment lengths. For example, two profiles with fragment abundances of 1–2–1–2–1 and 1–1–1–2–2 have identical Shannon entropy values, yet when analyzed using the size deconvolution method, they will result in different peak parameters with different entropies. In contrast, subtle sequential perturbations can be captured by Lorentzian component parameters in size deconvolution analysis, thereby enabling more sensitive quantification of fragmentation entropy.

### Phagocytosis increases the proportion of intra-nucleosomal cfDNA

To further investigate cfDNA size shortening, we analyzed fragment length distributions in patients undergoing radiotherapy. Paired plasma samples were obtained from eleven patients with stage I-III breast cancer, collected before (pre-) and after (post-) a first fraction of radiotherapy^35^. Post-radiotherapy blood samples were collected within 2.5 h after completion of the fraction. As shown in Fig. 3e, cfDNA from patients who received radiotherapy exhibited an enrichment of short fragments compared to those of the pre-treatment plasma samples. We applied cfDNA size deconvolution analysis and calculated intra-/inter-nucleosomal amplitude and entropy ratios between the pre- and post-radiotherapy plasma samples (median coverage: 0.9x; range: 0.2 to 3.7x). As shown in Fig. 3f, intra-/inter-nucleosomal amplitude ratio increased significantly after radiotherapy (*p* = 0.04, Wilcoxon signed-rank test; pre-radiotherapy median: 0.21; range: 0.13 to 0.31; post-radiotherapy median: 0.24, range: 0.17 to 0.34). On the other hand, the entropy ratio showed no significant difference (*p* = 0.21, Wilcoxon signed-rank test) between the pre-radiotherapy (median: 0.824, range: 0.813 to 0.850) and the post-radiotherapy plasma samples (median: 0.831, range: 0.809 to 0.864) (Fig. 3g). This contrasting pattern between the amplitude ratios (Fig. 3f) and entropy ratios (Fig. 3g) is reminiscent of that observed between healthy individuals and cancer-negative LFS patients (Fig. 3b, c), therefore may indicate shared fragmentation mechanisms responsible for non-tumor-related cfDNA shortening, which give rise to a higher proportion of intra-nucleosomal cfDNA but do not increase fragmentation entropy.

Radiation can induce both apoptotic and necrotic cell death, affecting both normal and cancer cells^36,37^. However, it has been reported that in patients receiving radiation therapy, even though the level of cell death increases substantially, total plasma cfDNA concentration in the peripheral blood does not rise significantly^38,39^. This apparent discrepancy may be explained, at least in part, by the role of phagocytosis^39^. After engulfing and digesting dead or dying cells, phagocytes may release both the digested DNA and their own DNA into circulation^40,41^. Therefore, we hypothesized that the enrichment of short DNA molecules in plasma samples of cancer-negative LFS patients (Fig. 3a), and post-radiotherapy patients (Fig. 3e), showing an increased intra-/inter-nucleosomal amplitude ratio (Fig. 3b, f) but without significant elevation in the entropy ratio (Fig. 3c, g), may not originate from DNA directly released by apoptotic or necrotic cells. Instead, it may be associated with intensified phagocytic activity.

### Constant cfDNA fragmentation entropy in organ transplantation

To validate our hypothesis that phagocytosis-associated cfDNA shortening does not significantly increase fragmentation entropy, we analyzed plasma samples from organ transplant recipients^42^. Monitoring donor-derived DNA (dd-cfDNA) in recipient plasma has proven feasible for detecting graft rejection, as graft injury or dysfunction leads to increased release of dd-cfDNA into the circulation^1^. Ng et al. reported that the short-to-long size ratio reflects dd-cfDNA levels in plasma of liver transplant recipients, enabling detection of early graft damage and rejection^43,44^.

We applied size deconvolution analysis to 14 plasma samples of liver transplant recipients, with a median dd-cfDNA fraction of 7.7% (range: 1.8% to 39.9%) and median coverage of 1.1x (range: 0.9 to 1.4x)^42^. Fragment length information was obtained from FinaleDB^45^. Based on dd-cfDNA fractions, samples were divided into low (< 10%; *n* = 7) and high (≥ 10%; *n* = 7) groups. As shown in Fig. 3h, recipient plasma cfDNA with high levels of dd-cfDNA exhibited shorter fragment length in comparison with the low dd-cfDNA group. The high dd-cfDNA group also showed significantly increased intra-/inter-nucleosomal amplitude ratios (median: 0.29; range: 0.23 to 0.43) compared with the low dd-cfDNA group (median: 0.21; range: 0.18 to 0.30; *p* = 0.03, Mann–Whitney *U *test) (Fig. 3i). However, there was no significant difference in the intra-/inter-nucleosomal entropy ratio between the high (median: 0.828; range: 0.815 to 0.841) and the low dd-cfDNA groups (median: 0.819; range: 0.812 to 0.829; *p* = 0.10, Mann–Whitney *U *test) (Fig. 3j), indicating a distinct fragmentation pattern that produces short non-malignant cfDNA without fragmentation entropy alteration, in contrast to ctDNA shortening.

### Differentiating benign disease and cancer using cfDNA size deconvolution

We hypothesized that the intra-/inter-nucleosomal entropy ratio, without being confounded by phagocytosis-mediated DNA fragmentation signals, could enable more accurate detection of tumor-derived ctDNA molecules. If correct, the intra-/inter-nucleosomal entropy ratio should demonstrate enhanced diagnostic performance in differentiating between patients with benign disease and cancer. To test this hypothesis, we analyzed plasma cfDNA samples from 48 patients with benign gastric disease and 50 patients with gastric cancer (median coverage: 1.1x, range 0.1 to 2.4x)^46^.

To better evaluate the utility of intra-/inter-nucleosomal amplitude and entropy ratios for cancer detection, we developed a refined size deconvolution algorithm for shallow sequencing data. It applies L2 regularization on component scales to mitigate scale overfitting, thereby preventing overestimation of fragmentation entropy (“Methods”). We further enhanced the robustness of this algorithm by incorporating constraints on the scale parameters, namely, requiring those paired-scale intra-nucleosomal components to have equal values and restricting inter-nucleosomal components above 167 bp to share the same scale value (“Methods”).

We performed cfDNA size deconvolution analysis and calculated intra-/inter-nucleosomal amplitude and entropy ratios for each individual. In addition, we assessed the proportion of short fragments (< 150 bp) and the short (100–150 bp) to long (151–220 bp) size ratio in the overall plasma cfDNA size profile of each plasma cfDNA sample (Supplementary Fig. S6). To avoid overfitting and data leakage when comparing the cancer detection performance of these metrics, we evaluated them directly without employing machine learning algorithms or performing cross-validation.

**Fig. 4:**
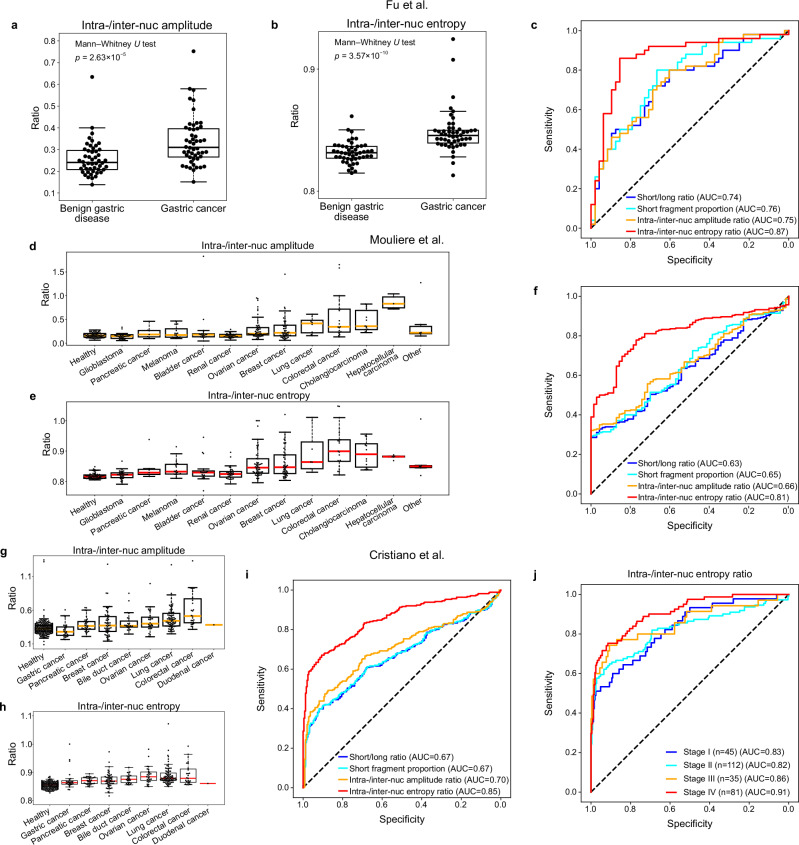
cfDNA size deconvolution analysis for cancer detection. Boxplots of intra-/inter-nuc **a** amplitude ratios, and **b** entropy ratios in plasma cfDNA of patients with benign gastric disease (*n* = 48) and gastric cancer (*n* = 50), with two-sided Mann–Whitney *U *tests applied. In each boxplot, the central line represents the median, the box denotes the interquartile range (IQR; 25% to 75% percentiles), and the whiskers extend to the most extreme values within 1.5 times IQR, with all individual data points shown. **c** ROC curves for the identification of gastric cancer (*n* = 50) from benign gastric disease (*n* = 48) using different metrics. Boxplots of intra-/inter-nuc **d** amplitude ratios, and **e** entropy ratios in plasma cfDNA of 70 healthy controls and 280 patients with various cancer types, including glioblastoma (*n* = 34), pancreatic cancer (*n* = 7), melanoma (*n* = 21), bladder cancer (*n* = 19), renal cancer (*n* = 33), ovarian cancer (*n* = 59), breast cancer (*n* = 53), lung cancer (*n* = 8), colorectal cancer (*n* = 21), cholangiocarcinoma (*n* = 14), hepatocellular carcinoma (*n* = 5), and other cancer types (*n* = 6), with central line, box, and whiskers of each boxplot defined as described above. **f** ROC curves for the differentiation between patients with (*n* = 280) and without (*n* = 70) cancer using different metrics. Boxplots of **g** intra-/inter-nuc amplitude ratios and **h** entropy ratios in plasma cfDNA of 247 healthy individuals and 291 patients with various cancer types, including gastric cancer (*n* = 27), pancreatic cancer (*n* = 34), breast cancer (*n* = 54), bile duct cancer (*n* = 25), ovarian cancer (*n* = 28), lung cancer (*n* = 95), colorectal cancer (*n* = 27), and duodenal cancer (*n* = 1), with central line, box, and whiskers of each boxplot defined as described above. **i** ROC curves for the differentiation between patients with (*n* = 291) and without (*n* = 247) cancer using different metrics. **j** ROC curves for the differentiation between patients across different tumor stages using intra-/inter-nuc entropy ratio. Source data are provided as a Source Data file.

Supporting our hypothesis, the intra-/inter-nucleosomal entropy ratio attained the highest area under the ROC curve (AUC) value of 0.87 for distinguishing patients with cancer from those with benign disease, with 24% sensitivity at 95% specificity (Fig. 4b, c). This AUC value is higher than those of the intra-/inter-nucleosomal amplitude ratio (AUC = 0.75; 24% sensitivity at 95% specificity; *p* = 0.03, DeLong test; Fig. 4a, c) and the short-to-long ratio (AUC = 0.74; 20% sensitivity at 95% specificity; *p =* 0.02, DeLong test; Fig. 4c). The entropy ratio showed a higher, though not significant, AUC than the short fragment proportion (AUC = 0.76; 26% sensitivity at 95% specificity; *p* = 0.05, DeLong test; Fig. 4c).

### Multi-cancer detection using cfDNA size deconvolution

We next attempted to apply size deconvolution analysis to plasma cfDNA of patients with various cancer types to evaluate its potential for multi-cancer detection. cfDNA size profiles from plasma samples of healthy donors (*n* = 70) and patients with various late-stage cancer types (*n* = 280) were obtained from a previous study^16^, with a median coverage of 0.5x (range: 0.002 to 1.5x). Remarkably, the intra-/inter-nucleosomal entropy ratio achieved the highest AUC value of 0.81 in differentiating patients with and without cancer, with 49% sensitivity at 95% specificity (Fig. 4e, f). The entropy ratio demonstrates higher performance than the intra-/inter-nucleosomal amplitude ratio (AUC = 0.66; 35% sensitivity at 95% specificity; *p* = 2.54 × 10^−14^, DeLong test; Fig. 4d, f) and the two conventional fragment-length–based methods (short fragment proportion: AUC = 0.65; 31% sensitivity at 95% specificity; *p* = 1.19 × 10^−12^, DeLong test; and short-to-long ratio: AUC = 0.63; 33% sensitivity at 95% specificity; *p* = 2.90 × 10^−16^, DeLong test; Fig. 4f, Supplementary Fig. S7).

We further validated our cfDNA size deconvolution analysis on an independent cohort from the DELFI study^17^, consisting of 247 plasma samples from healthy donors and 291 plasma samples from cancer patients across eight cancer types (stage I: *n* = 45; stage II: *n* = 112; stage III: *n* = 35; stage IV: *n* = 81; and unknown stage: *n* = 3), with a median coverage of 2.3x (range: 1.0 to 12.8x). Fragment length information was obtained from FinaleDB^45^. As shown in Fig. 4h, i, the intra-/inter-nucleosomal entropy ratio reached the highest AUC of 0.85 with 60% sensitivity at 95% specificity. This value was significantly higher than those of the other three metrics (short-to-long ratio: AUC = 0.67; 33% sensitivity at 95% specificity; *p* = 6.69 × 10^−20^, DeLong test; short fragment proportion: AUC = 0.67; 33% sensitivity at 95% specificity; *p* = 1.09 × 10^−19^, DeLong test; and intra-/inter-nucleosomal amplitude ratio AUC = 0.70; 38% sensitivity at 95% specificity; *p* = 7.90 × 10^−15^, DeLong test; Fig. 4g, i, Supplementary Fig. S8).

Next, we evaluated the performance of intra-/inter-nucleosomal entropy ratio for detecting cancer at different tumor stages. As shown in Fig. 4j, for identifying cancer patients with stages I, II, III, and IV diseases, it achieved sensitivities of 51%, 59%, 60%, and 68% at 95% specificity, with AUCs of 0.83, 0.82, 0.86 and 0.91, respectively. This demonstrates the potential of using the intra-/inter-nucleosomal entropy ratio in early-stage cancer detection. Together, the consistent performance of the entropy ratio across three cohorts (total *n* = 986) substantiates our hypothesis and demonstrates that cfDNA size deconvolution analysis can enhance cancer detection by targeting ctDNA-associated fragmentomic alterations.

## Discussion

In this study, we introduce a mathematical model that characterizes cfDNA fragment length distributions exclusively using the Lorentzian distribution. This approach demonstrates broad applicability to cfDNA derived from various cell types, across diverse physiological and pathological conditions, and in multiple bodily fluids. It enables investigation of the intrinsic fragmentation properties of cfDNA through the center, amplitude, and scale parameters of the Lorentzian distribution. The amplitude parameter of each Lorentzian component reflects the extent of protection conferred by the nucleosome structure against nuclease cleavage. In addition, the scale parameter corresponds to the entropy of the Lorentzian distribution, capturing cfDNA fragmentation entropy more sensitively than Shannon entropy^34^. Using deconvolutional analysis of cfDNA size profiles, we uncovered a component at ~159 bp, located between the apparent 10-bp periodic minor peaks and the 167-bp major peak. This 159-bp component may represent the boundary between intra-nucleosomal and inter-nucleosomal cfDNA fragments and therefore has importance for understanding and interpreting fragment size data, potentially paving the way for biomarker development.

In addition to quantitatively validating the suitability of the Lorentzian distribution for approximating cfDNA size profiles through *R*^*2*^ and residuals analysis, we identified two mathematical properties that further support the compatibility of the Lorentzian distribution for modeling fragment length distributions. First, a random variable following a Lorentzian distribution (e.g., the fragment length) can be expressed as the ratio of two Gaussian-distributed variables. Given that cfDNA molecules arise from the interplay between DNA nucleases cleavage^10,25^ and the protection by DNA-binding proteins^8^, this mathematical property might imply the underlying biological mechanism of cfDNA generation. Second, the Lorentzian distribution is a stable distribution^47^, meaning that a linear combination of two independent sets of Lorentzian-distributed variables results in a new set of variables that also follow the Lorentzian distribution. Because cfDNA consists of a mixture of DNA molecules released from various tissues, the overall cfDNA profile following a Lorentzian distribution implies that cfDNA from each constituent tissue also adheres to a Lorentzian distribution. Moreover, compared with algorithms that define 150 bp as the cutoff for short fragments^4,8,16–18,20,21,34^, the improved performance of the intra-/inter-nucleosomal amplitude ratio in multi-cancer detection demonstrates that Lorentzian-based size deconvolution is a more accurate approach for short cfDNA segmentation.

Through size deconvolution analysis, we elucidated that although phagocyte-induced cfDNA shortening increases the proportion of intra-nucleosomal cfDNA fragments, which is represented by amplitudes, it does not lead to a significant rise in fragmentation entropy. In contrast, tumor-relevant fragmentomic alterations induce markedly increased fragmentation entropy, as indicated by broader scales in intra-nucleosomal ctDNA molecules. These findings suggest distinct fragmentation pathways for tumor-associated cancer cell death and ctDNA fragmentation versus phagocyte-mediated DNA clearance during immunological processes. Further investigation is warranted to explore biological pathways and molecular mechanisms underlying these observations.

The intra-/inter-nucleosomal entropy ratio outperformed other fragment-length–based methods for multi-cancer detection, highlighting the potential of using deconvolution analysis of cfDNA fragment size for identifying ctDNA-associated fragmentomic features to enhance cancer diagnosis. Recently, Curtis et al. demonstrated that fragmentation patterns in ctDNA are reminiscent of those observed in non-tumor cfDNA from patients with autoimmune and vascular diseases^48^, highlighting several pathological contexts as potential confounders for cancer diagnosis when applying cfDNA fragmentomic analyses. Thus, integrating inflammatory status or vascular disease conditions into diagnostic models may help adjust for false-positive cfDNA fragmentomic signals.

In summary, this proof-of-concept study presents a unified analytical framework for investigating cfDNA fragment length distributions. The size deconvolution approach may help reveal information about nucleosomal structures associated with cfDNA degradation and provide preliminary insights into the mechanisms of cfDNA fragmentation and clearance. Our size model may also enable distinction between ctDNA-associated fragmentomic alterations and phagocyte-mediated fragmentation signatures, which could help differentiate early-stage tumors from premalignant lesions and aid in minimally invasive cancer diagnostics.

## Methods

### Study design

Paired-end whole-genome sequencing data were generated from pre- and post-radiotherapy plasma samples of 11 breast cancer patients in the Neo-RT cohort using SureSelect XT HS2 library preparation kit (Agilent)^35^. The Neo-RT (NCT03818100) trial was conducted in accordance with Good Clinical Practice guidelines and the Declaration of Helsinki. It received a favorable opinion from the Cambridge South Research Ethics Committee on 29/06/2017 (17/EE/0176).

Furthermore, we obtained paired-end cfDNA sequencing data from previous studies. These datasets include cfDNA from: 2 salivary samples (NEB Ultra II, New England Biolabs)^15^; 13 CSF samples (ThruPLEX Plasma-Seq, Rubicon Genomics)^13^; 18 healthy urinary cfDNA samples (ThruPLEX Plasma-Seq, Rubicon Genomics)^14^; 1 plasma cfDNA from a breast cancer patient (SureSelect XT HS Agilent)^33^; 19 healthy plasma cfDNA samples (SureSelect XT HS and XT HS2, Agilent)^23^; 28 lymphatic fluid cfDNA samples (TruSight Oncology 500, Illumina)^49^; a single-stranded plasma cfDNA sample (SRSLY PicoPlus DNA NGS Library Preparation Base Kit, Claret Bioscience)^32^, and a double-stranded plasma cfDNA sample (NEB Ultra II, New England Biolabs)^32^; 48 plasma cfDNA samples from patients with benign gastric disease^46^, and 50 plasma cfDNA samples of patients with gastric cancer^46^; and 350 plasma DNA samples from healthy individuals and cancer patients (ThruPLEX Plasma-Seq and Tag-Seq, Rubicon Genomics)^13^. We also obtained fragment length information from 14 plasma cfDNA samples from patients with liver transplantation (KAPA Library Preparation Kit, Kapa Biosystems)^42^; and 538 plasma DNA samples of healthy donors and cancer patients in the DELFI cohort (NEBNext DNA Library Prep Kit, New England Biolabs) from FinaleDB^45^. Size profiles of 199 plasma DNA samples of healthy donors and LFS patients were obtained from Wong et al. (KAPA Hyper Prep Kit, Kapa Biosystems)^24^. Additionally, two xenograft mouse plasma cfDNA samples were obtained from Sauer et al. (ThruPLEX Tag-Seq; Takara Bio)^30^.

### Statistics and reproducibility

Correlations between variables were calculated using Pearson’s correlation coefficient implemented using the SciPy Python module (version 1.17.0). Hypothesis testing was performed using non-parametric tests, including the Mann–Whitney *U *test (for two-group comparisons), the Wilcoxon signed-rank test (for paired samples comparisons), and the Kruskal–Wallis test (for multi-group comparisons). All statistical tests were two-sided, and a type I error rate of 5% was used to determine statistical significance. ROC curves and AUC values analyses were performed using the Scikit-learn Python module (version 1.7.0). No data were excluded from the analyses.

### cfDNA library preparation and sequencing

Peripheral whole blood from patients with breast cancer from the Neo-RT cohort^35^ was collected into EDTA tubes and processed within 1 h of venipuncture using a two-step centrifugation protocol. Samples were first centrifuged at 1600 *g* for 10 min to separate plasma from the buffy coat, followed by a second centrifugation of the plasma supernatant at 16,000 *g* for 10 min to remove residual cellular debris. cfDNA was extracted from 4 mL of plasma using the QIAamp Circulating Nucleic Acid Kit (Qiagen). Extracted cfDNA was quantified using the TapeStation cfDNA assay (Agilent) and stored at −80 °C prior to library preparation. Approximately 5 ng of cfDNA was used for library preparation. Libraries were generated according to the manufacturer’s instructions using the SureSelect XT HS2 kit (Agilent) and sequenced on an Illumina NovaSeq 6000 platform with 150-bp paired-end reads.

### Analysis of sequencing data

Adapter trimming was performed using the Agilent Genomics NextGen Toolkit (AGeNT). Paired-end cfDNA fragments were aligned to the human reference genome (GRCh38) using BWA-MEM (v0.7.18). PCR duplicates in the aligned reads were identified and excluded by using MarkDuplicates (Picard v3.2.0), followed by sorting and indexing with SAMtools (v1.20). The lengths of the cfDNA molecules were extracted from fragments with mapping qualities greater than 30 using the Python module pysam (v0.22.1).

### Analysis of cfDNA in xenograft mouse model

Paired-end cfDNA fragments from mouse plasma were simultaneously aligned to the human (GRCh38) and mouse (GRCm38) genomes using BWA-MEM (v0.7.18). PCR duplicate removal followed the same procedure as described above. Only DNA molecules uniquely aligned to the human genome with a mapping quality greater than 30 were retained. The lengths of the human cfDNA molecules were extracted using the Python module pysam (v0.22.1).

### Analysis of cfDNA in genomic regions with copy number aberrations

Plasma cfDNA molecules from a patient with breast cancer were subjected to copy number analysis using ichorCNA (v0.2.0). Genomic regions with copy-number neutral (*N* = 2), gain (*N* = 3), and amplification (*N* = 4 to 8) were identified from the "Corrected_Copy_Number" column in the segmentation file output by ichorCNA. Genomic regions with subclonal copy number changes (subclone.status = 1 in ichorCNA output) were excluded. cfDNA molecules mapping to these regions were then aggregated into categories for cfDNA size deconvolution analysis.

### cfDNA size deconvolution analysis

The number of cfDNA molecules of each size was first converted from absolute counts to relative frequencies as percentages. We hypothesize that the cfDNA fragment length distribution can be modeled as a sum of Lorentzian distributions, as represented by the formula below:

1 $$f(x)={\sum }_{i=1}^{n}\frac{{A}_{i}}{\pi }\left[\frac{{\sigma }_{i}}{{(x-{\mu }_{i})}^{2}+{{\sigma }_{i}}^{2}}\right]$$

where $x$ represents the fragment size, and $n$ denotes the number of Lorentzian distributions. Each distribution is defined by three parameters: the center ($\mu$), the scale ($\sigma$), and the amplitude ($A$). We utilized a non-linear curve fitting algorithm to deconvolute cfDNA size profiles, using an in-house Python script based on LMFIT module (v1.3.2). After deconvolution, approximate values for the three parameters of each peak (referred to as a component) were obtained. In analysis, the number of Lorentzian components ($n$) may vary according to apparent fragment length frequency patterns.

### cfDNA size deconvolution analysis with constraints and L2 regularization

To ensure robust size deconvolution analysis in shallow sequencing data, which may exhibit irregular, non-smooth fragment length distribution, we further optimized the generic size deconvolution method described above. To enhance robustness and reduce the degrees of freedom during the size deconvolution analysis, components at ~60 and ~70 bp, ~80 and ~90 bp, ~101 and ~111 bp, ~121 and ~131 bp, as well as ~141 and ~151 bp were constrained to have paired scale values (if applicable). Additionally, for components at ~167, ~177, ~188, ~199, ~210, and ~221 bp or above, scales were also restricted to share a common value (if applicable). Since component entropy depends only on its scale, to prevent overfitting of entropy, we applied L2 regularization on the sum of all component scales. Each scale was constrained between 2 bp and 8 bp.

### Analysis of intra-/inter-nucleosome amplitude and entropy ratios

After cfDNA size deconvolution analysis, the resulting components located less than 159 bp were defined as intra-nucleosomal components and components located equal to and above 159 bp were defined as inter-nucleosomal components. An intra-/inter-nucleosome amplitude ratio was defined as the average amplitude of all intra-nucleosomal components divided by that of inter-nucleosomal components. The intra-/inter-nucleosome entropy ratio was defined as the average entropy of all intra-nucleosomal components divided by that of inter-nucleosomal components. The entropy ($h$) of a Lorentzian component is calculated as:

2 $$h=\log (4\pi \sigma )$$

where $\sigma$ represents the scale parameter, and $\pi$ is a constant representing the ratio of a circle's circumference to its diameter.

### Reporting summary

Further information on research design is available in the Nature Portfolio Reporting Summary linked to this article.

## Supplementary information

Supplementary Information

Reporting Summary

Transparent Peer Review file

## Source data

Source Data

## Data Availability

The shallow WGS data of the Neo-RT cohort generated in this study have been deposited in the European Genome-Phenome Archive (EGA) (https://ega-archive.org) under accession code EGAD50000002238. Applicants will be asked to complete an information sheet providing a brief summary of the project for which the data will be used. All requests for access will be reviewed within 4–6 weeks by the Office of Translation and Impact at the Cancer Research UK Cambridge Institute to perform standard due diligence checks. Enquiries should be directed to the contact person, Sarah McGuire (email: OTI@cruk.cam.ac.uk). Raw sequencing data are available from EGA under the accession numbers EGAD00001004425^13^, EGAS00001003530^14^, EGAS00001008051^23^, EGAD00001006293^33^, EGAD00001004590^42^ and EGAD00001008650^30^; National Center for Biotechnology Information (NCBI) (https://www.ncbi.nlm.nih.gov/sra) with the accession numbers PRJNA999038^15^, PRJNA1032008^49^ and PRJNA1031581^46^; and FinaleDB^45^. Source data are provided with this paper.

## Code availability

The source code of the cfDNA fragment size deconvolution approach can be found in the Rosenfeld Group GitHub, https://github.com/nrlab-CRUK/cfDNA_size_deconvolution.
