# Supplementary material for: Cell-free DNA size deconvolution resolves nucleosomal origins and reveals tumor-associated fragmentomic alterations
Source: Nat Commun. 2026 May 8;17:6226. doi: 10.1038/s41467-026-72925-4 (PMC13369908; doi:10.1038/s41467-026-72925-4)
Supplement: Supplementary file 1 — Supplementary Information [file 41467_2026_72925_MOESM1_ESM.pdf]

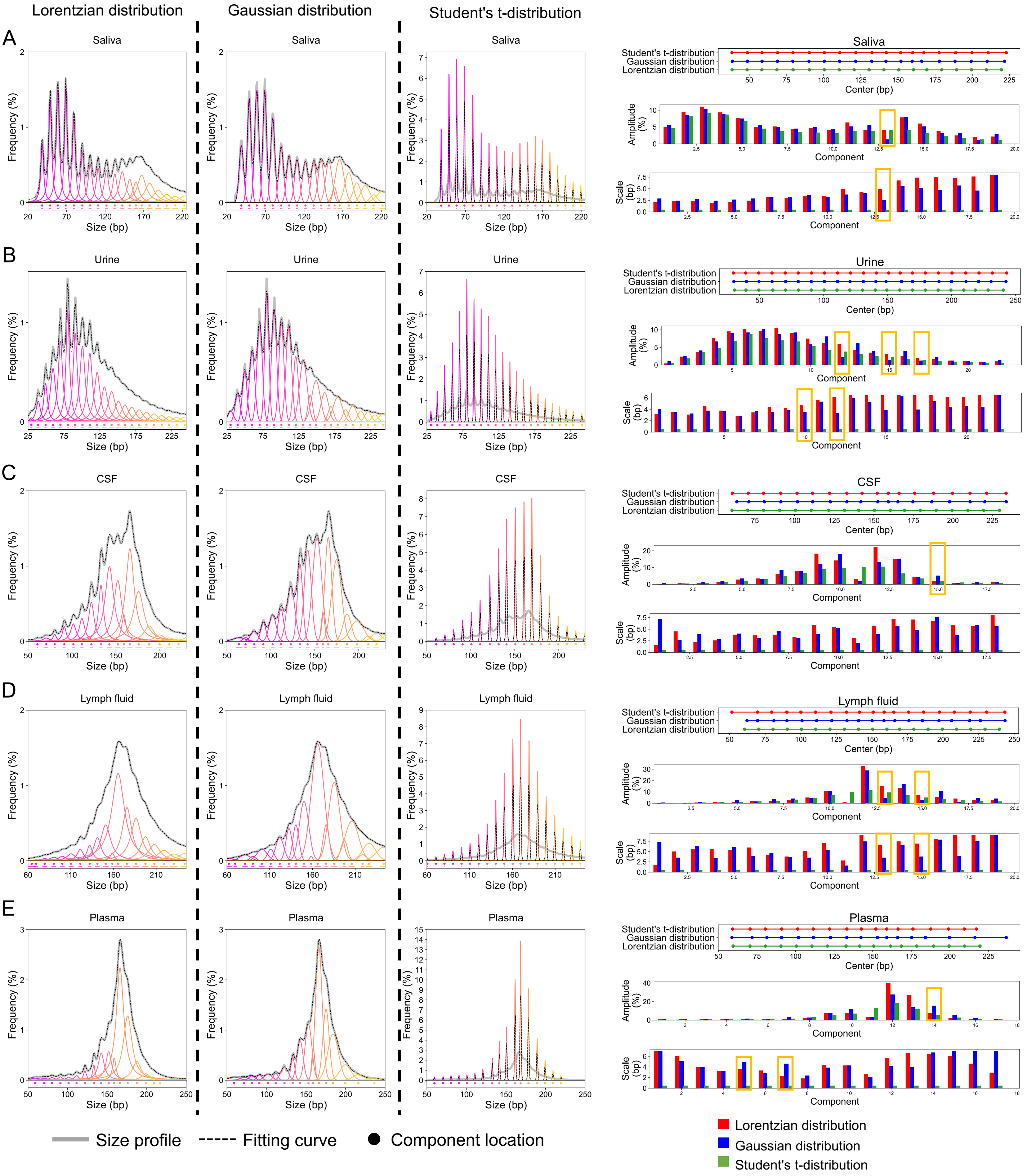

Lorentzian distribution
Gaussian distribution
Student's t-distribution
A
B
C
D
E
Saliva
Urine
CSF
Lymph fluid
Plasma
Frequency (%)
Size (bp)
Center (bp)
Amplitude (%)
Scale (bp)
Component
Size profile
Fitting curve
Component location
Lorentzian distribution
Gaussian distribution
Student's t-distribution

**Fig S1: Comparison of fitting results from different mathematical models in cfDNA size deconvolution analysis.** Size deconvolution analysis results of cfDNA from (**A**) saliva (27.1 million fragments from two samples; 19 peaks), (**B**) urine (149.9 million fragments from 18 samples; 22 peaks), (**C**) cerebrospinal fluid (CSF) (76.6 million fragments from 13 samples; 18 peaks), (**D**) lymphatic (lymph) fluid (906.6 million fragments from 28 samples; 18 peaks), and (**E**) plasma samples (497.1 million fragments from 19 samples; 17 peaks). Fitting results using the Lorentzian distribution are shown in the first column, Gaussian distribution in the second column, and Student's *t*-distribution (with degrees of freedom = 5) in the third column. All models use the same number of peaks, with each peak parameterized by three degrees of freedom (center, amplitude, and scale). Detailed information about component centers, amplitudes, and scales is shown in the fourth column, with irregular parameter values highlighted in yellow frames. Source data are provided as a Source Data file.

| | Bodily fluid | Distribution | $R^2$ |
|---|---|---|---|
| A | Saliva | Lorentzian | **0.99628036** |
| | | Gaussian | 0.98037246 |
| | | Student's t | -2.58702270 |
| B | Urine | Lorentzian | **0.99624745** |
| | | Gaussian | 0.99193350 |
| | | Student's t | 0.24397542 |
| C | CSF | Lorentzian | **0.99952503** |
| | | Gaussian | 0.99911664 |
| | | Student's t | 0.26789946 |
| D | Lymph fluid | Lorentzian | **0.99997845** |
| | | Gaussian | 0.99996673 |
| | | Student's t | 0.18350547 |
| E | Plasma | Lorentzian | **0.99978503** |
| | | Gaussian | 0.99973512 |
| | | Student's t | -0.53752270 |

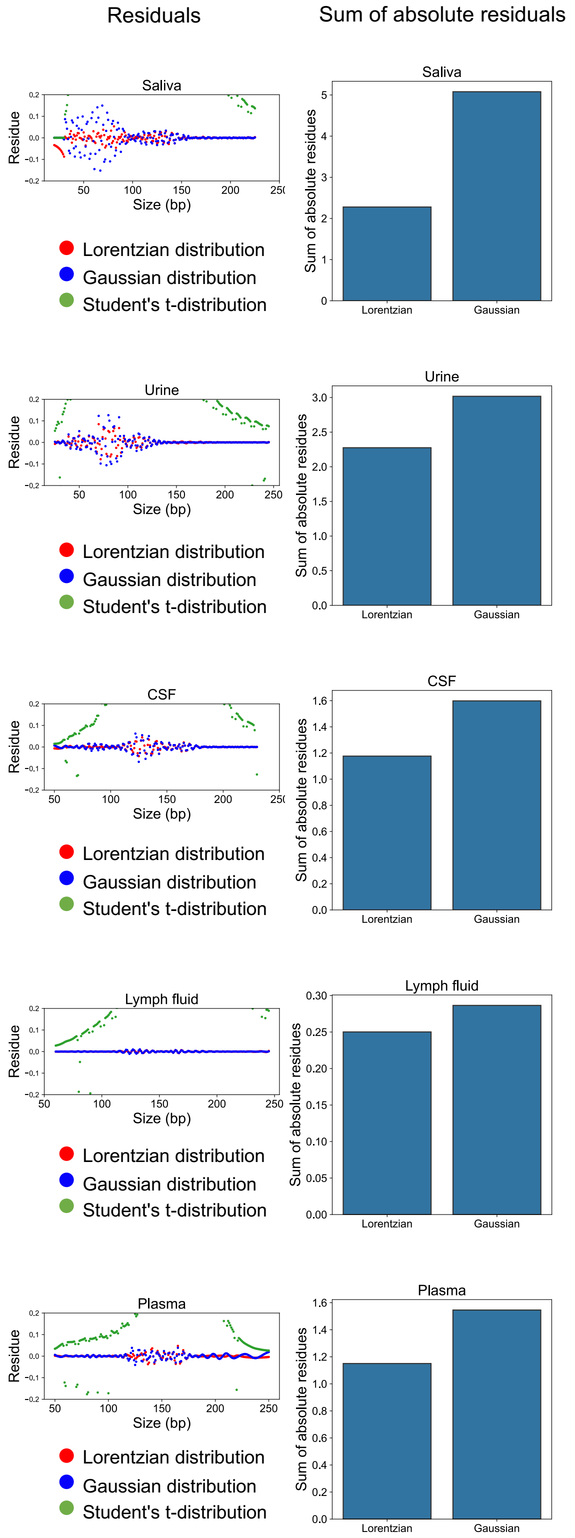

**Fig S2: Comparison of fitting statistics from different mathematical models in cfDNA size deconvolution analysis.** Size deconvolution analysis results of cfDNA from (**A**) saliva, (**B**) urine, (**C**) cerebrospinal fluid (CSF), (**D**) lymphatic (lymph) fluid, and (**E**) plasma samples using Lorentzian, Gaussian, and Student's *t* functions (same analyses as those in Fig S1). R-squared, or the coefficient of determination ($R^2$) values, are shown in the first column table. An $R^2$ value of 1 indicates a perfect fit, and values closer to 1 indicate better model performance. The $R^2$ values closest to 1 are shown in bold for each bodily fluid analysis. The size-banded fitting residuals for each base pair (i.e., the difference between the original size profile and the sum of deconvoluted components) are shown in the second column (range: −0.2 to 0.2). The sum of the absolute values of all residuals is shown in the third column. Source data are provided as a Source Data file.

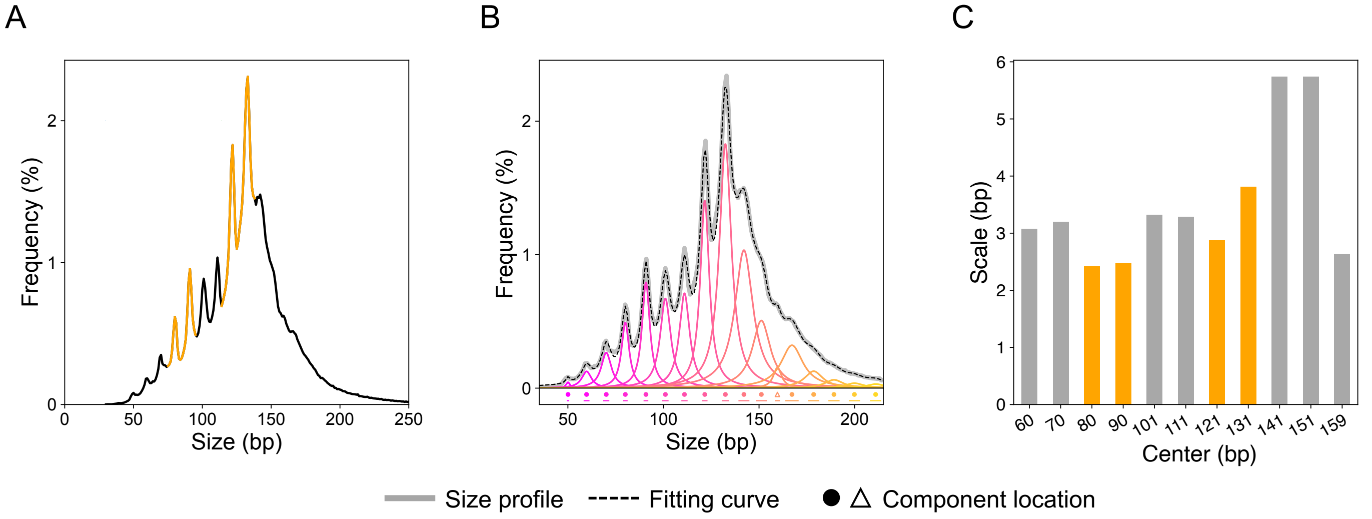


**Fig S3: Size deconvolution analysis of human cfDNA from a xenograft mouse model.** (**A**) Size profile of human cfDNA from xenograft mouse model (1.3 million fragments), with yellow lines indicating paired peaks displaying narrower widths compared to neighboring peaks. (**B**) Size deconvolution analysis of human cfDNA from the xenograft model. A triangle in lower panel indicates component at ~159 bp. (**C**) Bar plot of the components scales of xenografted human cfDNA. Source data are provided as a Source Data file.

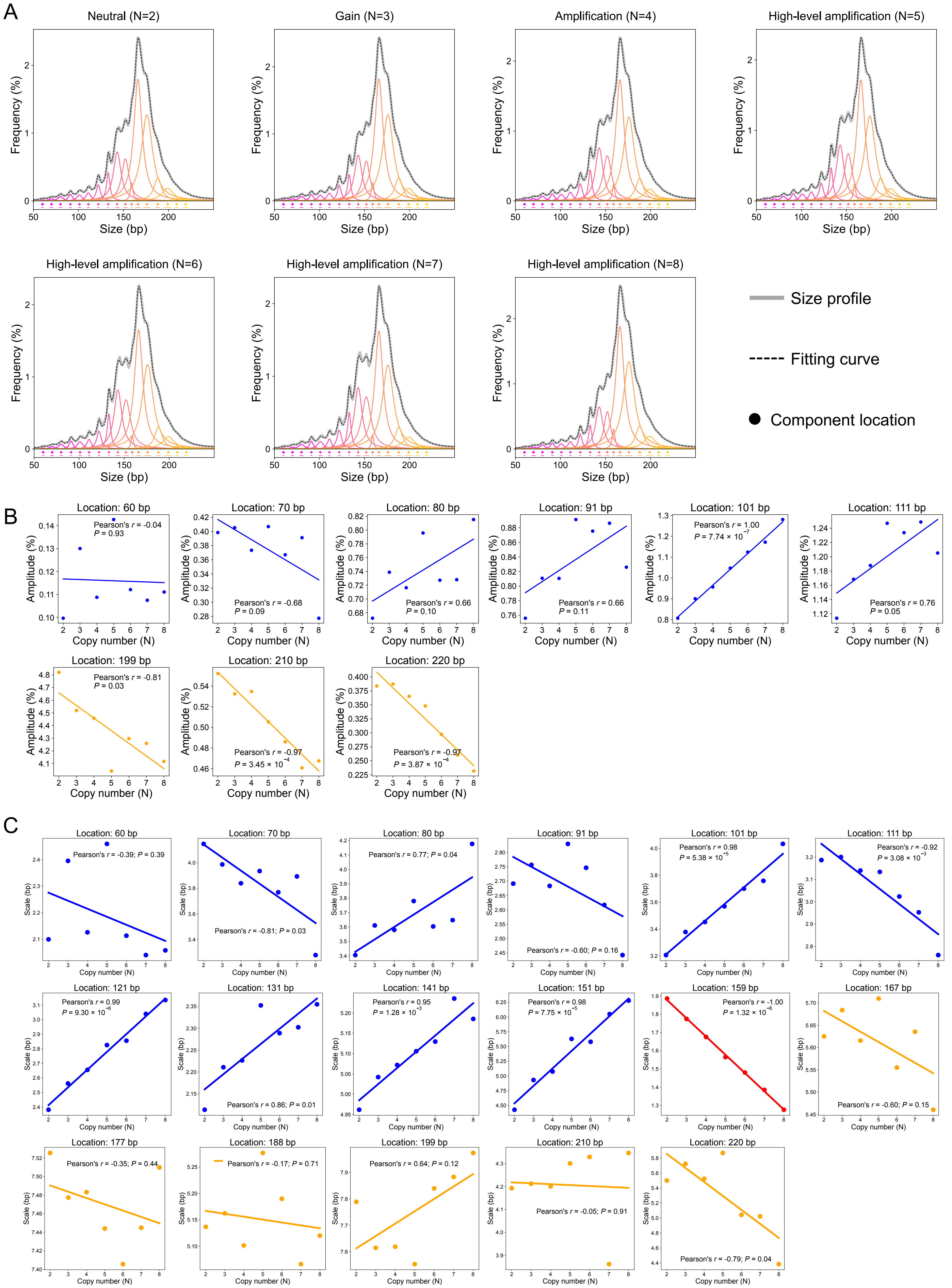
A
Neutral (N=2)
Gain (N=3)
Amplification (N=4)
High-level amplification (N=5)
High-level amplification (N=6)
High-level amplification (N=7)
High-level amplification (N=8)
Frequency (%)
Size (bp)
Size profile
Fitting curve
Component location
B
Location: 60 bp
Pearson's r = -0.04
P = 0.93
Location: 70 bp
Pearson's r = -0.68
P = 0.09
Location: 80 bp
Pearson's r = 0.66
P = 0.10
Location: 91 bp
Pearson's r = 0.66
P = 0.11
Location: 101 bp
Pearson's r = 1.00
P = 7.74 × 10^-7
Location: 111 bp
Pearson's r = 0.76
P = 0.05
Location: 199 bp
Pearson's r = -0.81
P = 0.03
Location: 210 bp
Pearson's r = -0.97
P = 3.45 × 10^-4
Location: 220 bp
Pearson's r = -0.97
P = 3.87 × 10^-4
Amplitude (%)
Copy number (N)
C
Location: 60 bp
Pearson's r = -0.39; P = 0.39
Location: 70 bp
Pearson's r = -0.81; P = 0.03
Location: 80 bp
Pearson's r = 0.77; P = 0.04
Location: 91 bp
Pearson's r = -0.60; P = 0.16
Location: 101 bp
Pearson's r = 0.98
P = 5.38 × 10^-5
Location: 111 bp
Pearson's r = -0.92
P = 3.08 × 10^-3
Location: 121 bp
Pearson's r = 0.99
P = 9.30 × 10^-6
Location: 131 bp
Pearson's r = 0.86; P = 0.01
Location: 141 bp
Pearson's r = 0.95
P = 1.28 × 10^-3
Location: 151 bp
Pearson's r = 0.98
P = 7.75 × 10^-5
Location: 159 bp
Pearson's r = -1.00
P = 1.32 × 10^-8
Location: 167 bp
Pearson's r = -0.60; P = 0.15
Location: 177 bp
Pearson's r = -0.35; P = 0.44
Location: 188 bp
Pearson's r = -0.17; P = 0.71
Location: 199 bp
Pearson's r = 0.64; P = 0.12
Location: 210 bp
Pearson's r = -0.05; P = 0.91
Location: 220 bp
Pearson's r = -0.79; P = 0.04
Scale (bp)
Copy number (N)

**Fig S4: Size deconvolution analysis of cfDNA derived from different tumor-associated copy number regions.** (**A**) Size deconvolution analysis results of cfDNA from different genomic regions, including copy number neutral (N = 2; 48.7 million fragments), tumor-associated gain (N = 3; 46.9 million fragments), amplification (N = 4; 7.3 million fragments), and high-level amplification regions (N = 5 to 8; 9.6, 3.5, 3.9, 2.0 million fragments, respectively). (**B**) Correlation plots of tumor-associated copy number versus the amplitudes of deconvoluted components located at ~60 to ~111 bp (blue lines and dots) and ~199 to ~220 bp (yellow lines and dots). (**C**) Correlation plots of tumor-associated copy number versus scales of deconvoluted components located at ~60 to ~151 bp (blue lines and dots), 159 bp (red line and dots), and ~167 to ~220 bp (yellow lines and dots). Source data are provided as a Source Data file.

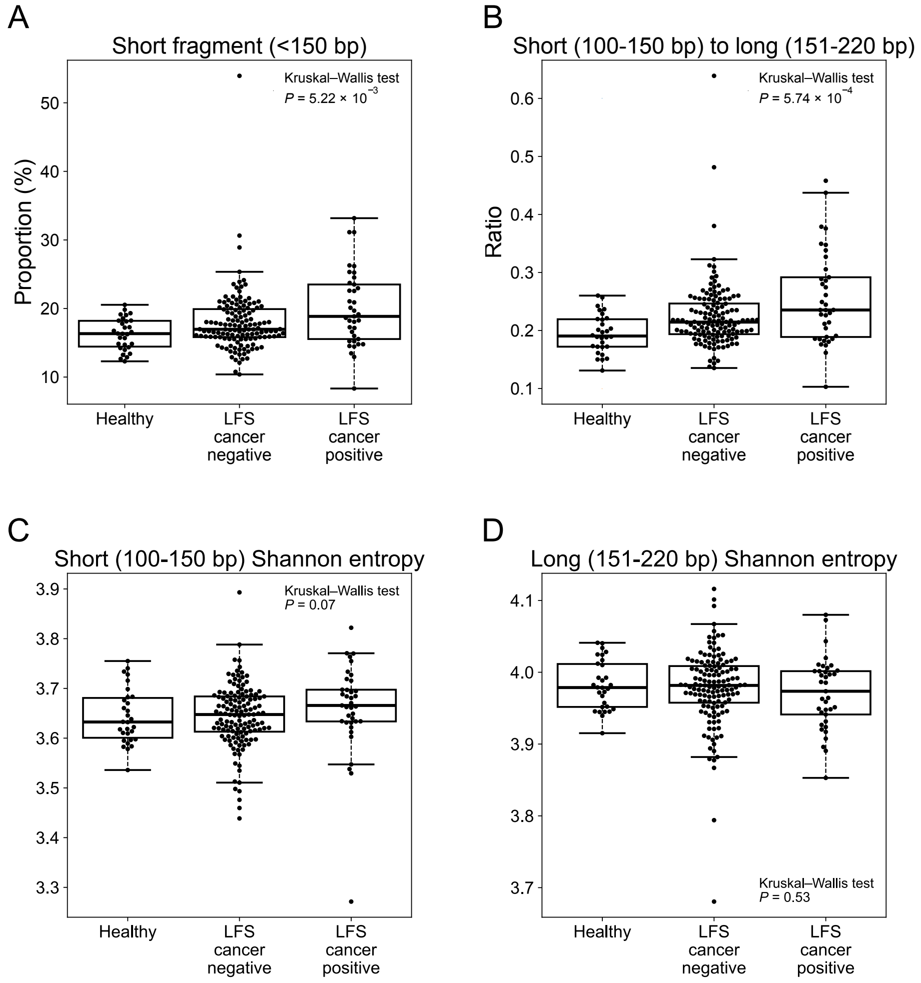


**Fig S5: Comparison of different fragment-length–based metrics in patients with and without LFS.** Boxplot of (**A**) short fragment (< 150 bp) proportion and (**B**) short (100−150 bp)-to-long (151−220 bp) size ratio, (**C**) short (100−150 bp) fragments Shannon entropy, and (**D**) long (151−220 bp) fragments Shannon entropy in healthy individuals (n = 30), LFS patients without cancer (n = 131), and LFS patients with active cancer (n = 38). In each boxplot, the central line represents the median, the box denotes the interquartile range (IQR; 25% to 75% percentiles), and the whiskers extend to the most extreme values within 1.5 times IQR, with all individual data points shown. Source data are provided as a Source Data file.

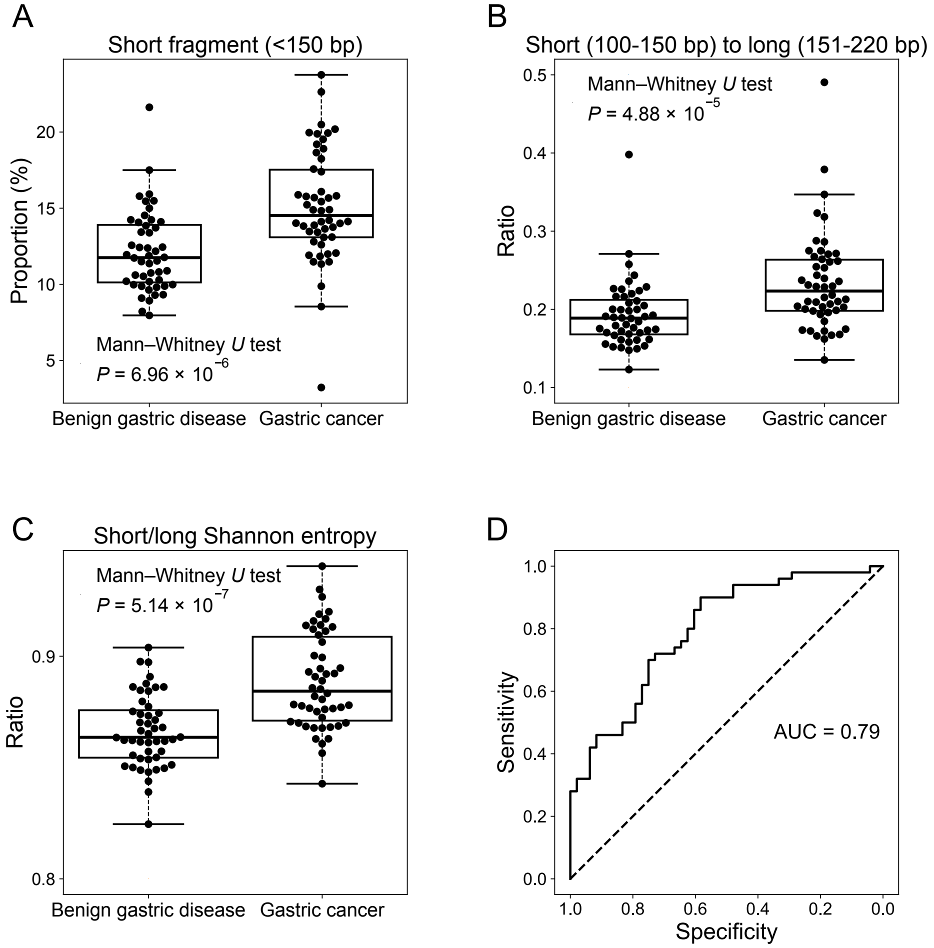


**Fig S6: Cancer detection in dataset from Fu et al.** Boxplots of (**A**) short fragment (< 150 bp) proportions, (**B**) short (100−150 bp)-to-long (151−220 bp) size ratios, and (**C**) short (100−150 bp)-to-long (151−220 bp) DNA Shannon entropies in overall plasma cfDNA of 48 patients with benign gastric disease and 50 patients with gastric cancer. In each boxplot, the central line represents the median, the box denotes the interquartile range (IQR; 25% to 75% percentiles), and the whiskers extend to the most extreme values within 1.5 times IQR, with all individual data points shown. (**D**) ROC curve differentiating between patients with (n = 50) and without (n = 48) cancer using short-to-long size Shannon entropy ratio. Source data are provided as a Source Data file.

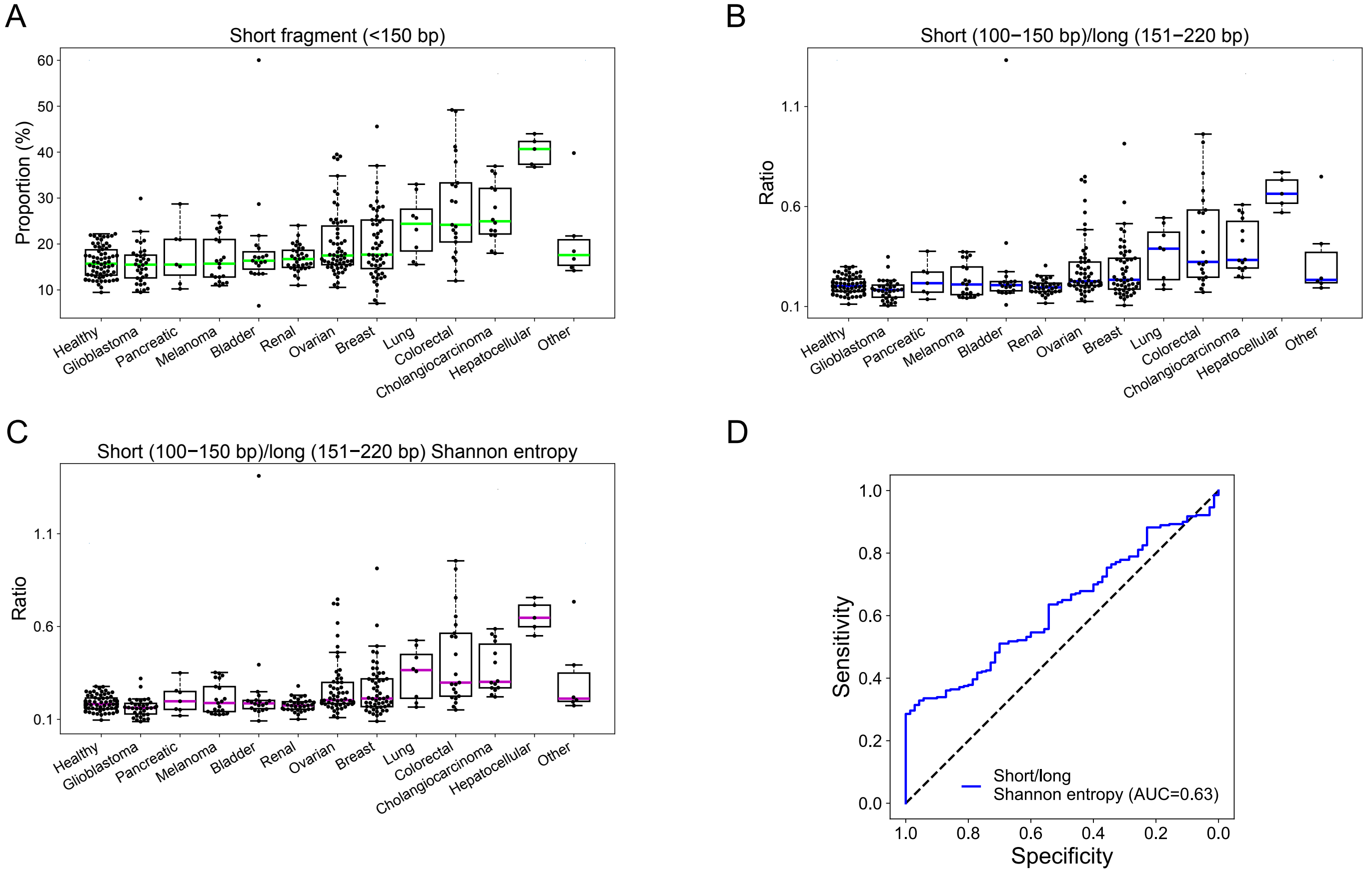


**Fig S7: Cancer detection in  from dataset Mouliere et al.** Boxplots of (**A**) short fragment (< 150 bp) proportions, (**B**) short (100−150 bp)-to-long (151−220 bp) size ratios, and (**C**) short (100−150 bp)-to-long (151−220 bp) DNA Shannon entropies in overall cfDNA of 70 healthy controls and 280 patients with various cancer types, including glioblastoma (n = 34), pancreatic cancer (n = 7), melanoma (n = 21), bladder cancer (n = 19), renal cancer (n = 33), ovarian cancer (n = 59), breast cancer (n = 53), lung cancer (n = 8), colorectal cancer (n = 21), cholangiocarcinoma (n = 14), hepatocellular carcinoma (n = 5), and other cancer types (n = 6). In each boxplot, the central line represents the median, the box denotes the interquartile range (IQR; 25% to 75% percentiles), and the whiskers extend to the most extreme values within 1.5 times IQR, with all individual data points shown. (**D**) ROC curve differentiating between patients with (n = 280) and without (n = 66) cancer using short-to-long size Shannon entropy ratio. Source data are provided as a Source Data file.

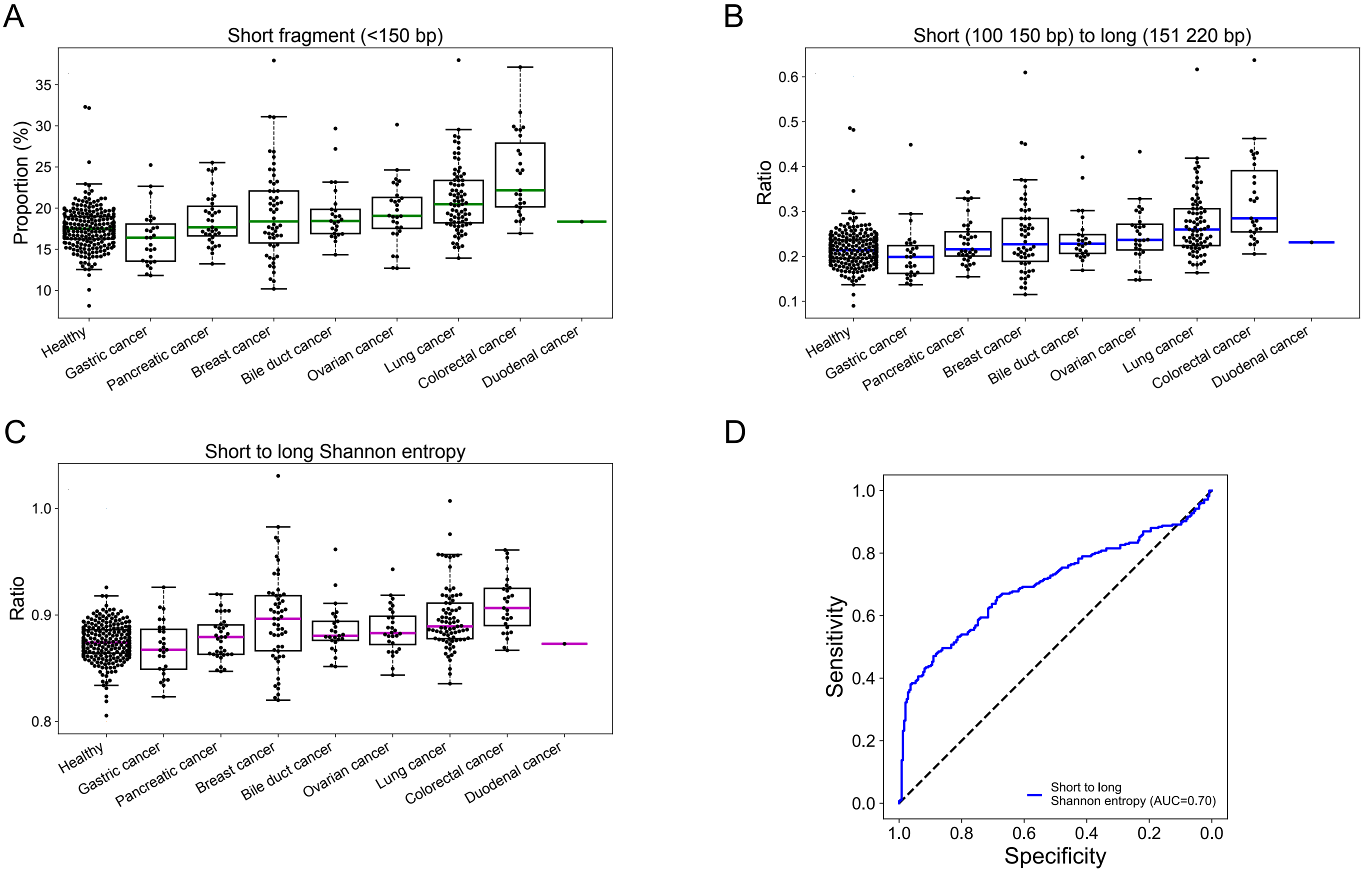


**Fig S8: Cancer detection in the DELFI dataset.** Boxplots of (**A**) short fragment (< 150 bp) proportions, (**B**) short (100−150 bp)-to-long (151−220 bp) size ratios, and (**C**) short (100−150 bp)-to-long (151−220 bp) DNA Shannon entropies in overall plasma cfDNA of 247 healthy controls and 291 patients with various cancer types, including gastric cancer (n = 27), pancreatic cancer (n = 34), breast cancer (n = 54), bile duct cancer (n = 25), ovarian cancer (n = 28), lung cancer (n = 95), colorectal cancer (n = 27), and duodenal cancer (n = 1). In each boxplot, the central line represents the median, the box denotes the interquartile range (IQR; 25% to 75% percentiles), and the whiskers extend to the most extreme values within 1.5 times IQR, with all individual data points shown. (**D**) ROC curve differentiating between patients with (n = 291) and without (n = 247) cancer using short-to-long size Shannon entropy ratio. Source data are provided as a Source Data file.
