# Supplementary material for: Cell-free DNA size deconvolution resolves nucleosomal origins and reveals tumor-associated fragmentomic alterations
Source: Nat Commun. 2026 May 8;17:6226. doi: 10.1038/s41467-026-72925-4 (PMC13369908; doi:10.1038/s41467-026-72925-4)
Supplement: Supplementary file 2 — Reporting Summary [file 41467_2026_72925_MOESM2_ESM.pdf]

nature portfolio

Corresponding author(s): Ze Zhou, Nitzan Rosenfeld, Hui Zhao

Last updated by author(s): Mar 7, 2026

Reporting Summary

Nature Portfolio wishes to improve the reproducibility of the work that we publish. This form provides structure for consistency and transparency in reporting. For further information on Nature Portfolio policies, see our Editorial Policies and the Editorial Policy Checklist.

Statistics

For all statistical analyses, confirm that the following items are present in the figure legend, table legend, main text, or Methods section.

n/a	Confirmed	
☐	☒	The exact sample size (*n*) for each experimental group/condition, given as a discrete number and unit of measurement
☐	☒	A statement on whether measurements were taken from distinct samples or whether the same sample was measured repeatedly
☐	☒	The statistical test(s) used AND whether they are one- or two-sided<br>*Only common tests should be described solely by name; describe more complex techniques in the Methods section.*
☒	☐	A description of all covariates tested
☒	☐	A description of any assumptions or corrections, such as tests of normality and adjustment for multiple comparisons
☐	☒	A full description of the statistical parameters including central tendency (e.g. means) or other basic estimates (e.g. regression coefficient) AND variation (e.g. standard deviation) or associated estimates of uncertainty (e.g. confidence intervals)
☐	☒	For null hypothesis testing, the test statistic (e.g. *F*, *t*, *r*) with confidence intervals, effect sizes, degrees of freedom and *P* value noted<br>*Give P values as exact values whenever suitable.*
☒	☐	For Bayesian analysis, information on the choice of priors and Markov chain Monte Carlo settings
☒	☐	For hierarchical and complex designs, identification of the appropriate level for tests and full reporting of outcomes
☐	☒	Estimates of effect sizes (e.g. Cohen's *d*, Pearson's *r*), indicating how they were calculated

Our web collection on statistics for biologists contains articles on many of the points above.

Software and code

Policy information about availability of computer code

Data collection	For NGS data collection, we used EGA download client, pyEGA3, and NCBI SRA Toolkit, sra-tools.
Data analysis	For NGS data analysis, we used BWA (v0.7.18) for sequence alignment, SAMtools (v1.20) for processing, and Picard (v3.2.0) for marking duplicate reads. We developed in-house Python (v3.12) scripts to extract DNA fragment lengths and perform cfDNA size profile deconvolution analysis.

For manuscripts utilizing custom algorithms or software that are central to the research but not yet described in published literature, software must be made available to editors and reviewers. We strongly encourage code deposition in a community repository (e.g. GitHub). See the Nature Portfolio guidelines for submitting code & software for further information.

Data

Policy information about availability of data

All manuscripts must include a data availability statement. This statement should provide the following information, where applicable:

- Accession codes, unique identifiers, or web links for publicly available datasets
- A description of any restrictions on data availability
- For clinical datasets or third party data, please ensure that the statement adheres to our policy

Raw sequencing data could be accessed in European Genome-Phenome Archive (EGA) (https://www.ega-archive.org) with the accession numbers EGAD50000002238, EGAD00001004425, EGAS00001003530, EGAD00001006293, EGAD00001008650, EGAS00001008051 and EGAS00001008331, and National

Center for Biotechnology Information (NCBI) (https://www.ncbi.nlm.nih.gov/sra), with the accession numbers PRJNA999038, PRJNA1032008 and PRJNA1031581. All other data and results are available in the main text or the supplementary materials.

Research involving human participants, their data, or biological material

Policy information about studies with human participants or human data. See also policy information about sex, gender (identity/presentation), and sexual orientation and race, ethnicity and racism.

Reporting on sex and gender	Both male and female patients were eligible for enrollment in this study. For breast and ovarian cancer cohorts, only female patients were included, as these represent sex-specific cancer types. Analyses stratified by sex were not conducted.
Reporting on race, ethnicity, or other socially relevant groupings	This study included human plasma samples collected by multiple groups across diverse geographic regions. No analyses based on race, ethnicity, or other socially relevant groupings are presented in this manuscript.
Population characteristics	The population characteristics has been previously reported in different cohorts: Mouliere et al. Sci Transl Med. 2018, Wong et al. Nat Commun. 2024, Sun et al. Genome Res. 2019, Fu et al. Gastric Cancer. 2024, Cristiano et al. Nature. 2019, and Lightowlers et al. Clin Oncol (R Coll Radiol). 2025.
Recruitment	The recruitment policy have been previously describtbed in Lightowlers et al. Clin Oncol (R Coll Radiol). 2025. The trial (Neo-RT) is a single-arm feasibility study of neoadjuvant radiotherapy and endocrine therapy in women with ER+, HER2-breast cancer that is grade 1 or 2, with palpable size equal to or greater than 20mm.
Ethics oversight	Ethical approval has also been reported previously (Lightowlers et al., Clin Oncol [R Coll Radiol], 2025). The trial (Neo-RT) was conducted in accordance with Good Clinical Practice guidelines and the Declaration of Helsinki. It received a favourable opinion from the Cambridge South Research Ethics Committee on 29/06/2017 (17/EE/0176).

Note that full information on the approval of the study protocol must also be provided in the manuscript.

Field-specific reporting

Please select the one below that is the best fit for your research. If you are not sure, read the appropriate sections before making your selection.

☒ Life sciences ☐ Behavioural & social sciences ☐ Ecological, evolutionary & environmental sciences

For a reference copy of the document with all sections, see nature.com/documents/nr-reporting-summary-flat.pdf

Life sciences study design

All studies must disclose on these points even when the disclosure is negative.

Sample size	This study tested 4 cohorts for cancer detection. The first one that obtained from Wong et al. Nat Commun. 2024, consists of 30 plasma samples from healthy donors, 131 plasma samples from patients with pathogenic germline TP53 mutations (Li-Fraumeni syndrome, LFS) but without cancer, and 38 plasma samples from LFS patients with active cancer. The second cohort that obtained from Mouliere et al. Sci Transl Med. 2018, consists of plasma cfDNA from 66 healthy controls and 276 patients with various cancer types. The third one was obtained from Fu et al. Gastric Cancer. 2024, consists of plasma cfDNA from 48 patients with benign gastric disease and 50 patients with gastric cancer. The Delfi cohort was from Cristiano et al. Nature. 2019 and obtained from FinaleDB, consists of 247 plasma cfDNA samples of healthy donors and 291 samples of cancer patients.
Data exclusions	No sample was excluded in the analyses.
Replication	We demonstrated the reproducibility of the cfDNA size profile deconvolution analysis by applying it across muliple different bodily fluids and samples. Consistent results were observed across all samples.
Randomization	This study included one clincical trial. As described in Lightowlers et al., Clin Oncol [R Coll Radiol], 2025, Neo-RT trial is a non-randomised, single-arm feasibility study of neoadjuvant radiotherapy and endocrine therapy in women with early stage breast cancer. Besides, patients randomization were previously described in Mouliere et al 2018, Wong et al 2024.
Blinding	Blinding between participants and investigators was applied in the cancer detection analysis at the patient level. Additionally, an independent validation cohort was assessed in a blinded manner using size-based metrics to ensure unbiased evaluation of different algorithms.

Reporting for specific materials, systems and methods

We require information from authors about some types of materials, experimental systems and methods used in many studies. Here, indicate whether each material, system or method listed is relevant to your study. If you are not sure if a list item applies to your research, read the appropriate section before selecting a response.

Materials & experimental systems

n/a	Involved in the study	
☒	☐	Antibodies
☒	☐	Eukaryotic cell lines
☒	☐	Palaeontology and archaeology
☐	☒	Animals and other organisms
☐	☒	Clinical data
☒	☐	Dual use research of concern
☒	☐	Plants

Methods

n/a	Involved in the study	
☒	☐	ChIP-seq
☒	☐	Flow cytometry
☒	☐	MRI-based neuroimaging

Animals and other research organisms

Policy information about studies involving animals; ARRIVE guidelines recommended for reporting animal research, and Sex and Gender in Research

Laboratory animals	No animal experiment was include.
Wild animals	No wild animals included in this study.
Reporting on sex	In the ovarian cancer xenograft mouse model, only female mice were included due to the sex-specific nature of the disease.
Field-collected samples	No field-collected samples were involved.
Ethics oversight	The Neo-RT (NCT03818100) trail was conducted in accordance with Good Clinical Practice guidelines and the Declaration of Helsinki. It received a favorable opinion from the Cambridge South Research Ethics Committee on 29/06/2017 (17/EE/0176).

Note that full information on the approval of the study protocol must also be provided in the manuscript.

Clinical data

Policy information about clinical studies

All manuscripts should comply with the ICMJE guidelines for publication of clinical research and a completed CONSORT checklist must be included with all submissions.

Clinical trial registration	NCT03818100
Study protocol	The Neo-RT study (Lightowlers et al., Clin Oncol [R Coll Radiol], 2025) is a feasibility study evaluating pre-operative breast intensity-modulated radiotherapy (IMRT) in patients receiving neo-adjuvant hormonal treatment for breast cancer, with the primary aim of assessing whether IMRT combined with endocrine therapy could facilitate breast-conserving surgery. The study also explored potential reductions in mastectomy rates, predictive immunohistochemical, molecular, and imaging biomarkers of response, and modulation of immune infiltrates in tumors following radiotherapy. IMRT was delivered to the whole breast (plus nodal regions if indicated) as 40 Gy in 15 fractions over 3 weeks with a tumor boost if needed, 26 Gy in 5 fractions over 1 week with or without a sequential boost, or 28.5–30 Gy in 5 fractions over 5 weeks for patients with significant comorbidities. Hormonal treatment (Letrozole 2.5 mg or Tamoxifen ± GnRH for pre-menopausal women) began after IMRT and continued for 20 weeks. MRI was performed at week 4 of endocrine therapy and pre-operatively, with ultrasound as an alternative according to local practice. Following treatment, patients will be followed up for 5 years, including standard clinical follow-up per local protocol, annual mammographical follow-up, clinical assessment of late radiotherapy toxicity, annual completion of Breast-Q and post-radiotherapy PROMs questionnaires, and annual optoacoustic imaging.
Data collection	Blood samples were collected twice during radiotherapy and once pre-surgery for translational analyses.
Outcomes	The Neo-RT trial outcomes were previously described in Lightowlers et al., Clin Oncol [R Coll Radiol], 2025, it validated the feasible of delivering neoadjuvant radiotherapy and endocrine therapy for oestrogen receptor-positive breast cancers.

Plants

Seed stocks	NA
Novel plant genotypes	NA
Authentication	NA
