# Supplementary material for: Cell-free DNA size deconvolution resolves nucleosomal origins and reveals tumor-associated fragmentomic alterations
Source: Nat Commun. 2026 May 8;17:6226. doi: 10.1038/s41467-026-72925-4 (PMC13369908; doi:10.1038/s41467-026-72925-4)
Supplement: Supplementary file 3 — Transparent Peer Review file [file 41467_2026_72925_MOESM3_ESM.pdf]

nature portfolio

Peer Review File

Cell-free DNA size deconvolution resolves nucleosomal origins and reveals tumor-associated fragmentomic alterations

Corresponding Author: Dr Hui Zhao

This file contains all reviewer reports in order by version, followed by all author rebuttals in order by version.

Version 0:

Reviewer comments:

Reviewer #1

(Remarks to the Author)
In this manuscript, Zhou and colleagues present a mathematical framework for modeling cfDNA fragment length distributions using mixtures of Cauchy–Lorentz distributions. Applied to saliva, urine, CSF, and plasma samples, this model identifies periodic peaks spaced by approximately 10 bp, except for a distinct 159 bp peak whose amplitude is consistent across all fluid types. The authors propose that this peak reflects a boundary between intra- and inter-nucleosomal cfDNA fragments, which forms the basis for subsequent analyses. The model is later simplified (without sufficient methodological detail) to enable more robust fitting in additional sample types.
While this approach offers new perspectives, the work is limited in terms of novelty, validation, and demonstrated clinical utility. The absence of robust validation in larger cohorts and a lack of clear practical application lead the reviewer to not recommend publication in Nature Communications at this time.
Major comments:
1. The authors have overlooked a recent relevant study by the Velculescu Lab (van 't Erve et al., Nature Communications, 2024), which also utilizes mixtures of length distributions for tumor fraction estimation. The claimed superiority of the Cauchy over Gaussian models for cfDNA size density is not adequately substantiated and greatly limits the novelty of the work.
2. The underlying data for Figure 1 are not described. Key details such as the number of samples, sequencing coverage, and other relevant parameters are missing, hindering interpretation and reproducibility.
3. The fitting procedure for Figure 1 needs clarification. How many degrees of freedom were employed, and were optimization constraints applied?
4. The identification of the 159 bp peak appears arbitrary. Optimization solutions may not be globally optimal, and it is unclear whether 159 bp is genuinely superior to the widely adopted 150 bp threshold for "short" fragments, which has long been a standard in the cfDNA field [Snyder et al., Cell 2016; Jiang et al., PNAS 2015; Sun et al., PNAS 2015]. For the 159 bp component, the amplitude does not appear to carry more biologically, or clinically relevant information compared to previous work, but the scale parameter may be somewhat interesting; however, the reason for this is unclear and requires further justification. Overall, the section on "ctDNA exhibits increased amplitudes and scales of intra-nucleosomal components" does not seem to convey new insights given the existing literature, unless a specific novel aspect can be clarified.
5. The scale parameter of a Cauchy distribution proportionally increases its entropy. Previous work (Esfahani et al., Nat Biotech 2022) showed that entropy at gene promoters is correlated with expression. The reviewer suggests specifically testing whether intra-nucleosomal scale parameters at promoters correlate with expression—potentially more than mixed or inter-nucleosomal components—or determining if the intra-nucleosomal entropy (via the scale parameter) at promoters carries more information about the tumor compartment than the normal background.
6. The cancer detection section is poorly structured. Details regarding the classification model (type), featurization process (global versus bin-wise fitting similar to Cristiano et al., Nature 2019), and validation strategy (cross-validation versus label-free scoring) are unclear. Additionally, the reported AUC (0.72) is unimpressive given that this cohort is predominantly late stage.
Minor comments:
1. Figure 4d is not convincing; claims of enrichment should be accompanied by appropriate statistical testing.
2. Important methodological details, such as coverage, patient stage, and p-values, are missing throughout the text.

(Remarks on code availability)

Reviewer #2

(Remarks to the Author)
The authors present a Lorentzian model to decompose cfDNA fragment length distributions into multiple periodic components. By parameterizing each component with three variables: center, amplitude, and scale, the model identifies a transition point at ~159 bp that demarcates intra- from inter-nucleosomal cfDNA. The intra-/inter-nucleosomal amplitude ratio and scale ratio are proposed as new fragmentomic metrics, modestly distinguishing cancer-derived cfDNA from healthy samples (AUC ≈ 0.72). Overall, the work is mathematically elegant and offers a unifying parametric framework for cfDNA fragmentomics. However, the biological interpretation and quantitative validation of the model's superiority remain limited, and the translational significance is not yet fully established.
major comments:
1. The introduction of the Lorentz distribution as a fitting function for cfDNA fragment size is conceptually appealing, providing an analytical representation of the periodic fragment peaks that reflect nucleosomal structure. However, the biological interpretation remains largely descriptive. The proposed Intra-/Inter-nucleosomal amplitude ratio essentially restates known fragmentomic features, the short/long fragment proportion under a new mathematical form. The 159 bp cutoff, while derived from model fitting, lacks independent biochemical validation (e.g., MNase-seq). Additional evidence such as enzymatic digestion experiments would be helpful to substantiate this demarcation as a genuine biological boundary rather than a mathematical guess.
2. The inclusion of multiple body fluids and datasets demonstrates model generalizability, yet the biological message is diluted by overemphasis on mathematical fitting. The manuscript would benefit from clearer discussion on what new underlying biological mechanistic insights the model provides.
3. If the main contribution of this paper is to establish a mathematically justified "fit-in-one" framework that better represents cfDNA size distributions across fluids, this claim requires quantitative validation. The authors should include quantitative model comparison metrics, such as adjusted R^2, residual plots comparing Lorentz vs. Gaussian or log-normal fits, etc. Demonstrating that the Lorentz model quantitatively outperforms Gaussian or exponential alternatives would transform this work from a visually convincing model to a quantitatively validated one.
minor comments:
1. Prior studies would take 166bp as the modal cfDNA size of plasma, do you have any biological explanation for that using your hypothesis?
2. line 167-170, please define the "higher and narrower than neighboring peaks", the sentence here is confusing.
3. Figure 3d, the intra-/inter- amplitude ratio in y-axis is from 2-8, corresponding to copy number 2-8. If I understand correctly, averaged intra- amplitude shall be the mean amplitude from 81bp-152bp, averaged inter- amplitude shall be the mean amplitude from 159bp-221bp. The ratio shall not be as shown in y-axis.
4. line 342 shall be "combination" rather than "combiation"

(Remarks on code availability)

Version 1:

Reviewer comments:

Reviewer #1

(Remarks to the Author)

(Remarks on code availability)
The authors have addressed my comments thoroughly. However, several results still do not convincingly demonstrate that the more complex mathematical model yields meaningful new biological insight or a clear translational advantage. For example, Figure R3 is underwhelming: the Lorentzian model appears only marginally better than the Gaussian model.

Reviewer #2

(Remarks to the Author)
Thanks for the rebuttal and additional analyses. Overall, the revisions addressed my prior concerns. The authors now provide clearer methodological details and a more coherent biological interpretation. I think it's good to go.

(Remarks on code availability)
I'm not familiar with coding. I focused more on wet lab.

Open Access This Peer Review File is licensed under a Creative Commons Attribution 4.0 International License, which permits use, sharing, adaptation, distribution and reproduction in any medium or format, as long as you give appropriate credit to the original author(s) and the source, provide a link to the Creative Commons license, and indicate if changes were made.
In cases where reviewers are anonymous, credit should be given to 'Anonymous Referee' and the source.
The images or other third party material in this Peer Review File are included in the article's Creative Commons license, unless indicated otherwise in a credit line to the material. If material is not included in the article's Creative Commons license and your intended use is not permitted by statutory regulation or exceeds the permitted use, you will need to obtain permission directly from the copyright holder.
To view a copy of this license, visit https://creativecommons.org/licenses/by/4.0/

We sincerely appreciate the reviewers' valuable criticism and constructive comments, which have guided us to refine our research objectives, reconsider the study design, and comprehensively revise and strengthen the manuscript. Our detailed, point-by-point responses to the comments are provided below.

RESPONSE TO REVIEWERS' COMMENTS

Reviewer #1 (Remarks to the Author):

In this manuscript, Zhou and colleagues present a mathematical framework for modeling cfDNA fragment length distributions using mixtures of Cauchy–Lorentz distributions. Applied to saliva, urine, CSF, and plasma samples, this model identifies periodic peaks spaced by approximately 10 bp, except for a distinct 159 bp peak whose amplitude is consistent across all fluid types. The authors propose that this peak reflects a boundary between intra- and inter-nucleosomal cfDNA fragments, which forms the basis for subsequent analyses. The model is later simplified (without sufficient methodological detail) to enable more robust fitting in additional sample types.

While this approach offers new perspectives, the work is limited in terms of novelty, validation, and demonstrated clinical utility. The absence of robust validation in larger cohorts and a lack of clear practical application lead the reviewer to not recommend publication in Nature Communications at this time.

We are deeply grateful for the reviewers' thoughtful comments. In accordance with the suggestions, we enhanced the logical coherence of the narrative, performed comprehensive validation and demonstrated markedly improved detection performance in two independent large multi-cancer cohorts.

Major comments:

1. The authors have overlooked a recent relevant study by the Velculescu Lab (van 't Erve et al., Nature Communications, 2024), which also utilizes mixtures of length distributions for tumor fraction estimation. The claimed superiority of the Cauchy over Gaussian models for cfDNA size density is not adequately substantiated and greatly limits the novelty of the work.

Response: We thank the Reviewer for highlighting this important work. van't Erve et al. utilize a random forest model to predict Mutant Allele Frequency (MAF) based on a total of 16 features. These include mixture model weights (11 features), chromosomal arm-level aneuploidy scores (PA-score), maximum absolute z-scores (2 features), and principal components of short-to-long fragment length ratios (2 features). The mixture model approach was described in more detail by Carey et al. in a 2021 poster (Carey et al. *Journal of Clinical Oncology*, 2021), where they approximated fragment length distributions using a mixture of 12 truncated normal distributions (Figure R1). Their work, utilizing modified Gaussian distributions, also clearly demonstrates that the Gaussian distribution is not able to properly model the cfDNA size profile.

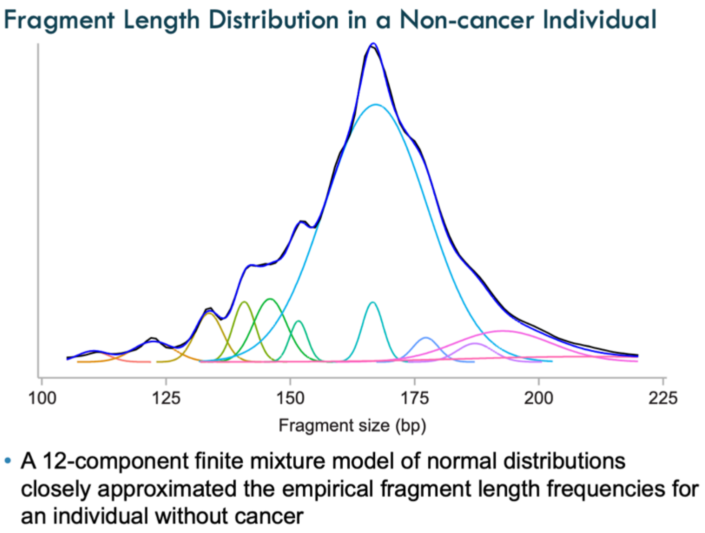

Figure R1. cfDNA size deconvolution using truncated Gaussian distributions (snapshot from Carey *et al.* Journal of Clinical Oncology 2021).

In the present work, our method employs the Cauchy–Lorentz distribution without any truncation or modification of the standard distribution, enabling us to more clearly capture cfDNA biology related to nucleosomal structures and tumor-specific fragmentation patterns. To prove superiority of the Cauchy over Gaussian models, also following Reviewer #2 comment #3, we have included additional analyses comparing Lorentzian, Gaussian, and Student's t-distribution models for cfDNA size deconvolution across five different bodily fluids. As shown in Figure R2, we applied cfDNA size deconvolution analysis using Lorentzian distribution, Gaussian distribution, and Student's t-distribution to cfDNA from saliva (Figure R2A), urine (Figure R2B), CSF (Figure R2C), lymphatic fluid (Figure R2D), and plasma (Figure R2E) samples. All models were run with the same parameter settings with same number of components. Both the Lorentzian (Figure R2 column 1) and Gaussian distributions (Figure R2 column 2) produced reliable fitting results, while the Student's t-distribution yielded unreliable fits. Its components exceeded the original size profiles and produced large residuals (Figure R2 column 3). The Lorentzian components yielded coefficients of determination (R^2; range: 0 to 1) approaching 1 in all five bodily fluids (all $R^2 > 0.99$), exceeding those of both the Gaussian and Student's *t* distributions (Figure R3 column 1). By evaluating size-banded fitting residuals (Figure R3 column 2) and the sum of absolute residuals (Figure R3 column 3), we demonstrate that the residuals from the Lorentzian model exhibited lower fluctuation strength and had lower sum of absolute residuals across all five bodily fluids compared to the Gaussian model. In particular, we included a new body fluid, lymphatic fluid (Figure R2D and 3D), in which the cfDNA length distribution forms a smoother curve without distinct sharp peaks. The Lorentzian model still showed the lowest sum of absolute residuals when modelling this distribution. Together, these results highlight the superiority of the Lorentzian distribution over the other models. We have included these result in Supplementary Fig. S1 and S2, and include van't Erve et al. and Carey et al. work in the introduction section.

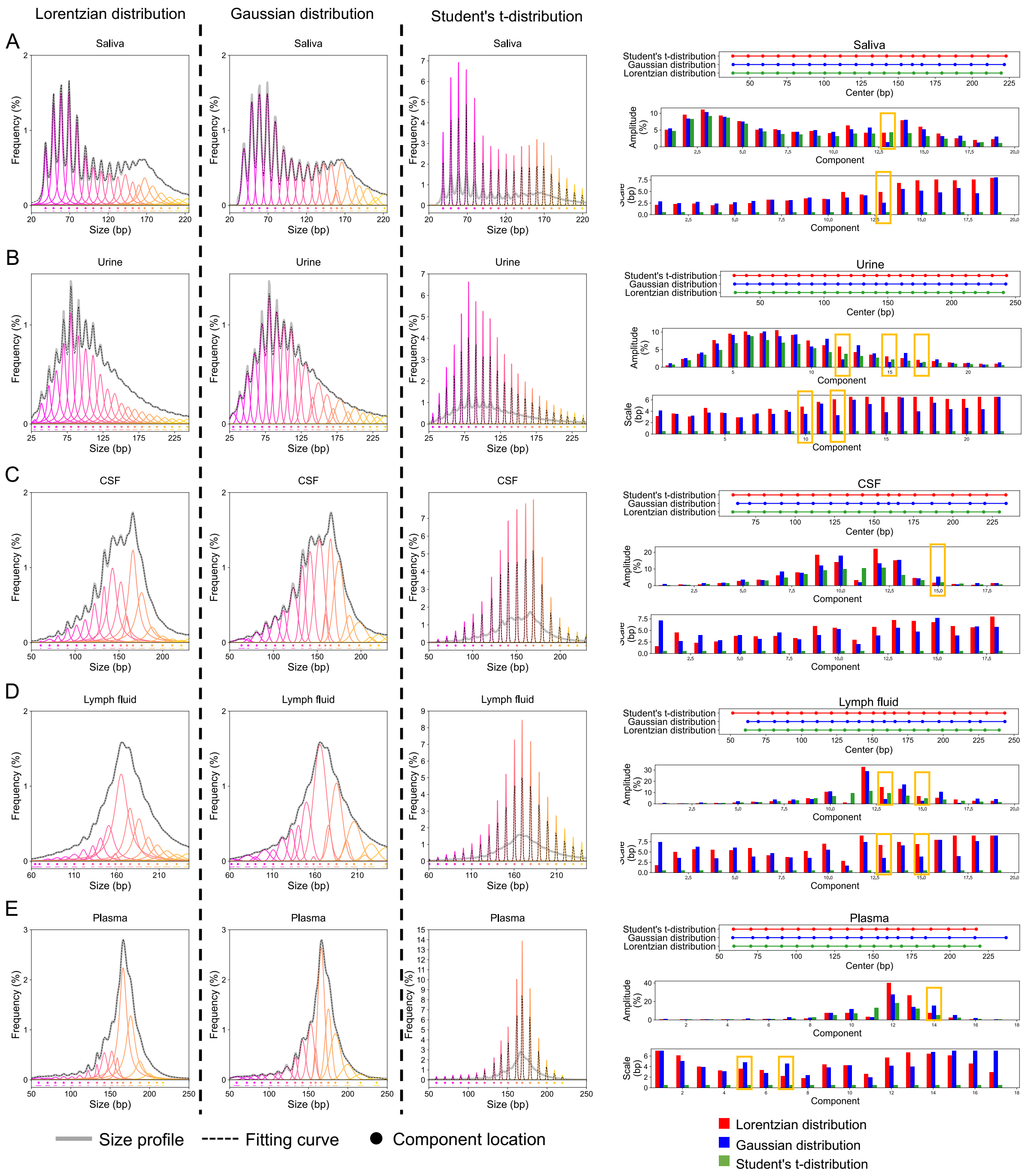

Figure R2. Comparison of fitting results from different mathematical models in cfDNA size deconvolution analysis. Size deconvolution analysis results of cfDNA from (**A**) saliva (27.1 million fragments from two samples; 19 peaks), (**B**) urine (149.9 million fragments from 18 samples; 22 peaks), (**C**) cerebrospinal fluid (CSF) (76.6 million fragments from 13 samples; 18 peaks), (**D**) lymphatic (lymph) fluid (906.6 million fragments from 28 samples; 18 peaks), and (**E**) plasma samples (497.1 million fragments from 19 samples; 17 peaks). Fitting results using the Lorentzian distribution are shown in the first column, Gaussian distribution in the second column, and Student's *t*-distribution (with degrees of freedom = 5) in the third column. All models use the same number of peaks, with each peak parameterized by three degrees of freedom (center, amplitude, and scale). Detailed information about component centers, amplitudes, and scales is shown in the fourth column, with irregular parameter values highlighted in yellow frames.

	Bodily fluid	Distribution	R^2
A	Saliva	Lorentzian	**0.99628036**
		Gaussian	0.98037246
		Student's t	-2.58702270
B	Urine	Lorentzian	**0.99624745**
		Gaussian	0.99193350
		Student's t	0.24397542
C	CSF	Lorentzian	**0.99952503**
		Gaussian	0.99911664
		Student's t	0.26789946
D	Lymph fluid	Lorentzian	**0.99997845**
		Gaussian	0.99996673
		Student's t	0.18350547
E	Plasma	Lorentzian	**0.99978503**
		Gaussian	0.99973512
		Student's t	-0.53752270

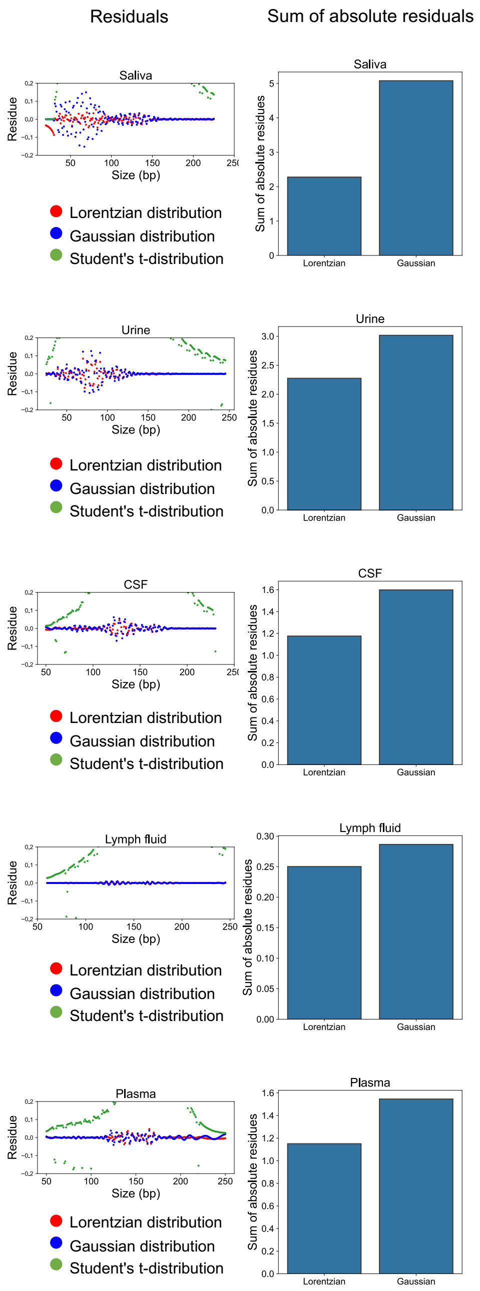

Figure R3: Comparison of fitting statistics from different mathematical models in cfDNA size deconvolution analysis. Size deconvolution analysis results of cfDNA from (**A**) saliva, (**B**) urine, (**C**) cerebrospinal fluid (CSF), (**D**) lymphatic (lymph) fluid, and (**E**) plasma samples using Lorentzian, Gaussian and student's *t* functions (same analyses as these of Supplementary Figure 1). R-squared, or the coefficient of determination (R^2) values, are shown in the first column table. An R^2 value of 1 indicates a perfect fit, and values closer to 1 indicate better model performance. The R^2 values closest to 1 is shown in bold for each bodily fluid analysis. The size-banded fitting residuals for each base pair (difference between the original size profile and the sum of deconvoluted components) are shown in the second column (range: -0.2 to 0.2). The sum of the absolute values of all residuals is shown in the third column.

2. The underlying data for Figure 1 are not described. Key details such as the number of samples, sequencing coverage, and other relevant parameters are missing, hindering interpretation and reproducibility.
Response: We thank the reviewer for the comment. We have now included more detail information on the number of samples and number of fragments for pooling samples; and added coverage statistics for all samples in all cohorts used in the size deconvolution analyses. The raw data of the size profiles from multiple bodily fluids (including additional lymphatic fluid cfDNA) presented in Figure 1 have also been added to Supplementary Table 1 to facilitate interpretation and ensure reproducibility. We also included a demonstration of size deconvolution analysis on both the Code Ocean website and GitHub (https://github.com/nrlab-CRUK/cfDNA_size_deconvolution).

3. The fitting procedure for Figure 1 needs clarification. How many degrees of freedom were employed, and were optimization constraints applied?
Response: We have now clarified the number of Lorentzian distributions applied in the deconvolution of cfDNA from different bodily fluids in Figure 1: (**C**) saliva (19 components), (**D**) urine (22 components), (**E**) CSF (18 components), (**F**) lymphatic fluid (18 components), and (**G**) plasma (17 components). Each component (peak) has three degrees of freedom (center, amplitude, and scale). In Figure 1, we pooled cfDNA from multiple bodily fluids to have smooth size profiles, e.g., saliva (27.1 million fragments), urine (149.9 million fragments), CSF (76.6 million fragments), lymphatic fluid (906.6 million fragments), and plasma samples (497.1 million fragments). For these high-depth samples, we did not apply any optimization constraints; we only restricted the maximum scale value below 9 bp and minimum scale value above 2 bp.

For sWGS data, we required those paired-scale intra-nucleosomal components have equal values, and restricting inter-nucleosomal components equal to or more than 167 bp to share the same scale value. In addition, guided by the Reviewer #1's suggestion #5, recognizing the importance of entropy, we applied L2 regularization on all component scales to prevent overfitting of fragmentation entropy.

4. The identification of the 159 bp peak appears arbitrary. Optimization solutions may not be globally optimal, and it is unclear whether 159 bp is genuinely superior to the widely adopted 150 bp threshold for "short" fragments, which has long been a standard in the cfDNA field [Snyder et al., Cell 2016; Jiang et al., PNAS 2015; Sun et al., PNAS 2015]. For the 159 bp component, the amplitude does not appear to carry more biologically, or clinically relevant information compared to previous work, but the scale parameter may be somewhat interesting; however, the reason for this is unclear and requires further justification. Overall, the section on "ctDNA exhibits increased amplitudes and scales of intra-nucleosomal components" does not seem to convey new insights given the existing literature, unless a specific novel aspect can be clarified.
Response: We thank the reviewer for the critical comment. We have added a new section titled "*159 bp serves as a demarcation between intra- and inter-nucleosomal cfDNA*" to the main text to clarify and reinforce the identification of the 159-bp peak as the boundary between intra- and inter-nucleosomal cfDNA. Also according to the Reviewer #2 comment #1, we now included a comparison between single-stranded cfDNA and conventional double-stranded cfDNA size profiles and further used the difference between ctDNA and cfDNA to demonstrate the biological significance of the 159-bp peak.

To determine whether the component at ~159 bp reflects a biologically meaningful nucleosomal structure or merely a mathematical artifact, we applied our method to plasma cfDNA prepared using single-stranded library protocol (ssDNA). Similar to double-stranded cfDNA (dsDNA), ssDNA exhibits a major peak at ~167 bp. Moreover, ssDNA has been reported to show ~3 bp offsets in the minor peaks of the fragment length distribution, which were also observed in Figure R4A. Our size deconvolution analysis provides a holistic view of fragment lengths for both dsDNA (Figure R4B; 16.9 million fragments) and ssDNA (Figure R4C; 27.9 million fragments) and allowed us to identify a previously unseen 159-bp component positioned between the minor (< ~150 bp) and major peaks (at ~167 bp), as well as additional components above the major peak at ~177 bp, ~188 bp, ~199 bp, and ~210 bp. In ssDNA (Figure R4C), we observed that all components below 159 bp exhibited a ~3 bp rightward offset compared to those of dsDNA (Figure R4B), suggesting a shared nucleosomal protection effect against enzymatic DNase digestion in short (< 159 bp) cfDNA fragments. By contrast, all components above 159 bp showed no obvious offset between ssDNA and dsDNA (Figure R4B and C), indicating a distinct fragmentation mechanism compared to that of short (< 159 bp) cfDNA. Together, these results suggest that the component centered at ~159 bp may be associated with biologically meaningful nucleosomal structures.

cfDNA fragments shorter than 150 bp, exhibiting a ~10 bp periodicity in size distributions, are thought to mainly originate from nucleosomal core particle. We hypothesized that the 159-bp DNA fragment might consist of 1 bp at the nucleosome dyad flanked by two 79 bp stretches of DNA (1+79+79 bp). As illustrated in the three-dimensional nucleosome structure (Figure R4D), both ends of the 79 bp segments flanking the dyad are tightly bound to linker histone H1. This aligns with our observation that the components below 159 bp show ~3 bp offsets between ssDNA and dsDNA, potentially reflecting protection conferred by nucleosome structures. Furthermore, the lower amplitude of the 159-bp component compared to the preceding component may indicate weaker protection strength conferred by linker histone H1 binding relative to core histones wrapping (Figure R4B and C).

To further explore this hypothesis, we analyzed ctDNA from a patient with stage IV breast cancer. As ctDNA is expected to be enriched in genomic regions showing tumor-associated copy number gains, we compared cfDNA molecules mapped to copy-number neutral regions (N = 2; 48.7 million fragments), regions with copy number gain (N = 3; 46.9 million fragments), amplification regions (N = 4; 7.3 million fragments), and high-level amplification regions (N = 5, 6, 7, and 8; fragments: 9.6, 3.5, 3.9, and 2.0 million, respectively). As shown in Figure R4E, cfDNA fragments derived from genomic regions with higher levels of tumor copy number gain, exhibit a progressive increase in short fragments, reflecting the increased ctDNA fraction. We next performed size deconvolution analysis on cfDNA from regions with varying copy numbers. Supporting our hypothesis (Figure R4F), components amplitudes showed opposing trends across the 159-bp boundary. With increasing tumor-associated copy number, the amplitudes of components at ~121 bp, ~131 bp, ~141 bp, and ~151 bp showed significant positive correlations (Pearson's $r \geqslant 0.99$, $P < 0.001$), whereas the amplitudes of components at ~159 bp, ~167 bp, ~177 bp, and ~188 bp showed strong negative correlations (Pearson's $r \leqslant -0.96$, $P < 0.001$), demonstrating altered fragmentation patterns in ctDNA shifting between intra- and inter-nucleosomal DNA. Together, these observations in ssDNA and ctDNA may reveal 159 bp as a previously unrecognized fragment length feature associated with the pivot point between intra- and inter-nucleosomal cfDNA fragments.

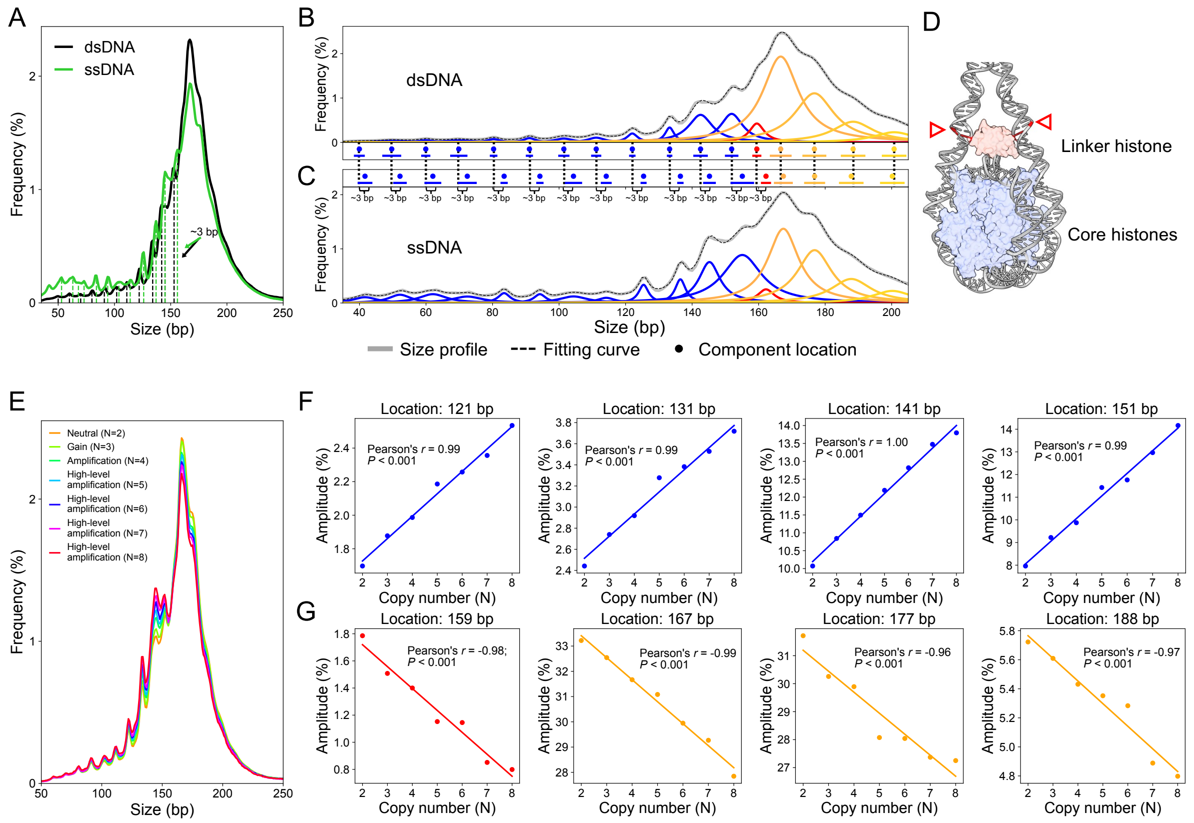

Figure R4. (**A**) Size profiles of double-stranded cfDNA (dsDNA; 16.9 million fragments) and single-stranded cfDNA (ssDNA; 27.9 million fragments). Size deconvolution analysis of (**B**) dsDNA and (**C**) ssDNA. (**D**) Illustration of a nucleosome with DNA wrapped around core histones (blue) and bound to linker histone H1 (red) (PDB: 5NL0). The red triangles indicate the two ends of a stretch of DNA spanning 159 nt, centered at the nucleosome dyad. (**E**) Size profiles of cfDNA molecules from different genomic regions, including tumor-associated copy number neutral (N = 2; 48.7 million fragments), gain (N = 3; 46.9 million fragments), amplification (N = 4; 7.3 million fragments), and high-level amplification regions (N = 5 to 8; 9.6, 3.5, 3.9, 2.0 million fragments, respectively). (**F, G**) Changes in amplitude parameters across tumor copy number status (N = 2 to 8) of Lorentzian components at ~121 bp to ~151 bp (blue), ~159 bp (red), and ~167 bp to ~188 bp (yellow)

5. The scale parameter of a Cauchy distribution proportionally increases its entropy. Previous work (Esfahani et al., Nat Biotech 2022) showed that entropy at gene promoters is correlated with expression. The reviewer suggests specifically testing whether intra-nucleosomal scale parameters at promoters correlate with expression—potentially more than mixed or inter-nucleosomal components—or determining if the intra-nucleosomal entropy (via the scale parameter) at promoters carries more information about the tumor compartment than the normal background.

Response: We are grateful to the reviewer for the insightful comment. Based on the reviewer's suggestion, we recognized that the entropy of a Lorentzian distribution is a function of its scale parameter, defined as the natural logarithm of the product of 4π and the scale, i.e., log(4πσ). Therefore, we conceptually interpret changes in the scale parameter as changes in cfDNA fragmentation entropy. Accordingly, we have refined the intra- to inter-nucleosomal scale ratio into an intra- to inter-nucleosomal entropy ratio in our analysis.

Based on the reviewer's interest in whether intra-nucleosomal entropy (via the scale parameter) at promoters correlates with gene expression, we obtained fragment length data from the original study (Esfahani et al., Nat. Biotechnol. 2022) [3]. As shown in Figure R5A, we first replicated the authors' analyses of cfDNA WGS fragment length heatmaps, gene expression profiles (GEP) of blood leukocytes assessed by RNA-seq, and promoter fragmentation entropy (PFE) distributions across 1,832 TSS of meta-gene, with each group encompassing 10 genes with similar expression levels. As shown in Figure R5B, we replicated the PFE developed by Esfahani. It showed a significant correlation with GEP (Pearson's $r = 0.90$, $P < 0.001$).

In total, we obtained 196.59 million fragments from 1 kb regions flanking these TSS groups (n = 1,832) and applied size deconvolution analysis to each TSS group, with an average of ~0.1 million fragments per group. As shown in Figure R5C, the entropy of the inter-nucleosomal components showed a significant correlation with gene expression levels assessed by RNA-seq (Pearson's $r = 0.74$, $P < 0.001$). Although Pearson's r value is slightly lower than the PFE metric, the inter-nucleosomal entropy nevertheless demonstrated feasibility for assessing cfDNA fragmentation entropy and showed potential for inferring gene expression. We argue that our Lorentzian distribution is more suitable for analyzing nucleosome-associated cfDNA, whereas promoter regions, where increased TF binding produces different signal, may require a more tailored algorithm (e.g., PFE) for gene expression inference.

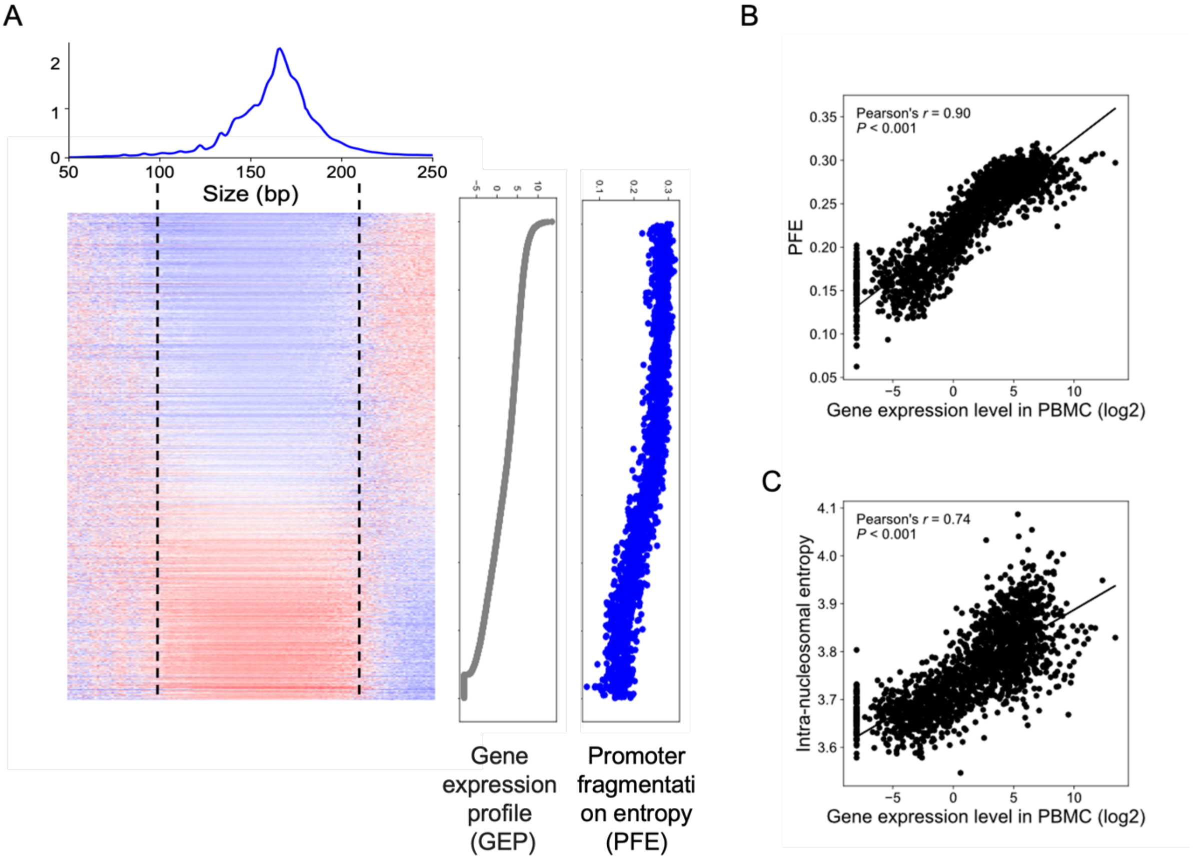

Figure R5. (**A**) Replicate analyses of plasma sample profile (blue line), heatmap capturing fragment density as ordered by gene expression profile (GEP) in blood leukocytes assessed by RNA-seq using TPM (grey line). Each row corresponds to one meta-gene encompassing the TSSs of ten genes when ranked by a reference PBMC expression vector. (**B**) Correlation

between gene expression profile (GEP) and promoter fragmentation entropy (PFE). (**C**) Correlation between GEP and inter-nucleosomal component entropy assessed by size deconvolution analysis.

We also included PFE in the analysis of the overall cfDNA fragment length distribution in Li-Fraumeni syndrome (LFS) patients. PFE showed no significant differences between healthy individuals and LFS patients with or without cancer in short fragments (Figure R6A; *P* = 0.08, Kruskal–Wallis test), long fragments (Figure R6B; *P* = 0.50, Kruskal–Wallis test), or their entropy ratio (Figure R6C; *P* = 0.20, Kruskal–Wallis test). However, intra-/inter-nucleosomal entropy ratio increase was considered as tumor specific fragmentation pattern and showed significant difference increase in LFS patients with cancer Figure R6D.

We reasoned that Shannon entropy measures the randomness of cfDNA fragment length distribution within a given range but does not account for the permutations of fragment lengths, which, however, can be captured by component parameters in the size deconvolution analysis. For example, two profiles with fragment abundances of 1–2–1–2–1 and 1–1–1–2–2 have identical Shannon entropy values, yet they would yield different peak parameters when analyzed by deconvolution.

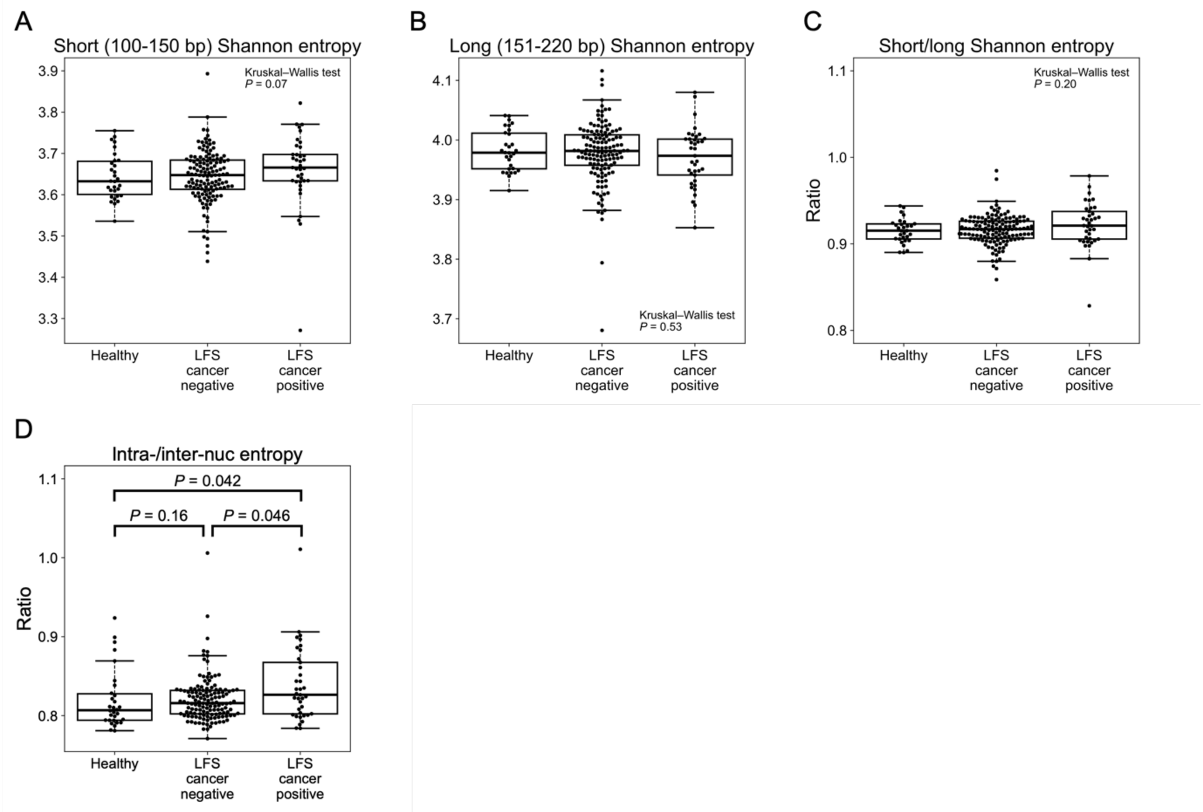

Figure R6: Boxplot of (**A**) short (100-150 bp) fragments Shannon entropy, (**B**) long (151-220 bp) fragments Shannon entropy and (**C**) their ratio in healthy individuals (n = 30), LFS patients without cancer (n = 131), and LFS patients with active cancer (n = 38). (**D**) Boxplot of intra-/inter-nucleosomal (intra-/inter-nuc) entropy ratios in healthy individuals (n = 30) and LFS patients with (n = 38) and without (n = 131) cancer.

6. The cancer detection section is poorly structured. Details regarding the classification model (type), featurization process (global versus bin-wise fitting similar to Cristiano et al., Nature 2019), and validation strategy (cross-validation versus label-free scoring) are unclear.

Additionally, the reported AUC (0.72) is unimpressive given that this cohort is predominantly late stage.

We thank the reviewer for the helpful comments. Based on the reviewer's suggestions, we have developed an intra-/inter-nucleosomal entropy ratio for cancer detection. To robustly apply size deconvolution analysis to sWGS, we developed an optimized size deconvolution algorithm. Briefly, we incorporate paired-scale constraints on intra-nucleosomal components and restrict all components above 167 bp to share the same scale value. Additionally, we apply L2 regularization across all component scales to prevent overfitting of fragmentation entropy.

We first obtained cfDNA fragment size profiles from healthy donors (n = 70) and patients with various late-stage cancer types (n = 280) from Mouliere et al. 2018, with a median coverage of 0.5x (range: 0.002 to 1.5x). We applied the size deconvolution analysis and calculated the intra-/inter-nucleosomal amplitude and entropy ratios for each individual. In addition, we examined the proportion of short fragments (<150 bp) and the short (100-150 bp) to long (151-220 bp) size ratio in overall plasma cfDNA size profile of each sample.

To objectively compare the ctDNA detection performance of these metrics, we did not apply machine learning algorithms and cross-validation to avoid overfitting and data leakage. Supporting our hypothesis, the intra-/inter-nucleosomal entropy ratio (Figure R7B) achieved the highest area under the ROC curve (AUC) value of 0.81 (Figure R7C) for differentiating patients with and without cancer, with 49% sensitivity at 95% specificity. The entropy ratio demonstrates significant superior performance over conventional fragment-length-based methods (short-to-long ratio: AUC = 0.63; $P < 0.001$, DeLong test; 31% sensitivity at 95% specificity; short fragment proportion: AUC = 0.65; $P < 0.001$, DeLong test; 33% sensitivity at 95% specificity; and intra-/inter-nucleosomal amplitude ratio: AUC = 0.66; $P < 0.001$, DeLong test; 33% sensitivity at 95% specificity).

Furthermore, we analyzed an additional independent cohort from the DELFI study (Cristiano et al. *Nature* 2019), consisting of 247 plasma cfDNA samples from healthy donors and 291 samples from cancer patients across eight cancer types (stage I: n = 45; stage II: n = 112; stage III: n = 35; stage IV: n = 81; and unknown stage: n = 3), with median coverage of 2.3x (range: 1.0 to 12.8x). As shown in Figure R7F, the intra-/inter-nucleosomal entropy ratio also achieved the highest AUC of 0.85 with 60% sensitivity at 95% specificity, significantly outperforming other metrics (short-to-long ratio: AUC = 0.67; $P < 0.001$, DeLong test; 33% sensitivity at 95% specificity; short fragment proportion: AUC = 0.67; $P < 0.001$, DeLong test; 33% sensitivity at 95% specificity; and intra-/inter-nucleosomal amplitude ratio AUC = 0.70; $P < 0.001$, DeLong test; 38 % sensitivity at 95% specificity).

We further analyzed the performance of the intra-/inter-nucleosomal entropy ratio for cancer detection across different tumor stages. As shown in Figure R7G, it achieved sensitivities of 51%, 59%, 60%, and 68% at 95% specificity (AUCs of 0.83, 0.82, 0.86 and 0.91) for identifying cancer patients at stages I, II, III, and IV, respectively, demonstrating potential in early-stage cancer detection. The superior performance of the intra-/inter-nucleosomal entropy ratio in the two large cohorts (totaling 888 samples) demonstrates that cfDNA size deconvolution analysis can enhance cancer detection by targeting ctDNA-specific fragmentation patterns.

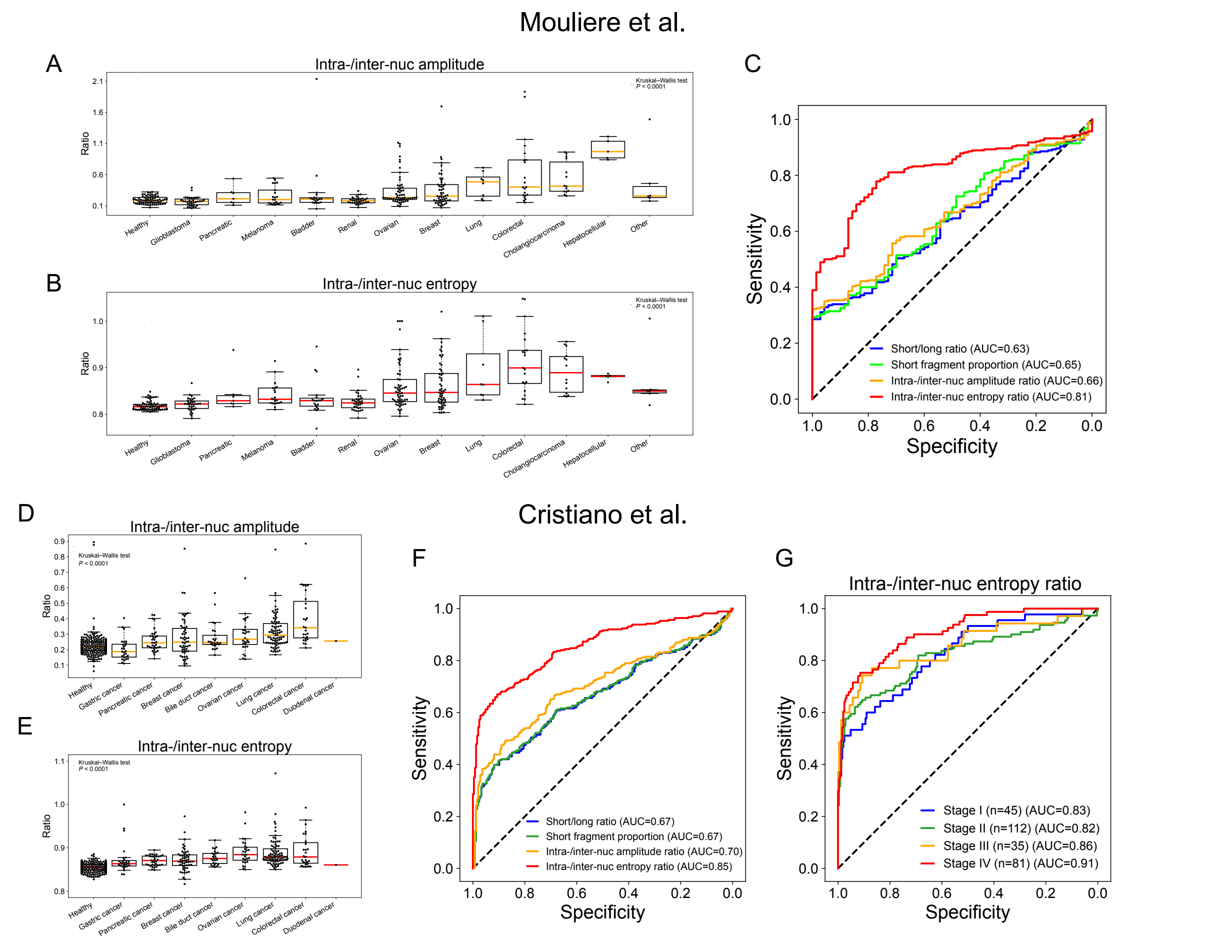

Figure R7. Boxplots of intra-/inter-nuc (**A**) amplitude ratios, and (**B**) entropy ratios in plasma cfDNA of 70 healthy controls and 280 patients with various cancer types, including glioblastoma (n = 34), pancreatic cancer (n = 7), melanoma (n = 21), bladder cancer (n = 19), renal cancer (n = 33), ovarian cancer (n = 59), breast cancer (n = 53), lung cancer (n = 8), colorectal cancer (n = 21), cholangiocarcinoma (n = 14), hepatocellular carcinoma (n = 5), and other cancer types (n = 6). (**C**) ROC curves for the differentiation between patients with (n = 280) and without (n = 70) cancer using different metrics. Boxplots of (**D**) intra-/inter-nuc amplitude ratios and (**E**) entropy ratios in plasma cfDNA of 247 healthy individuals and 291 patients with various cancer types, including gastric cancer (n = 27), pancreatic cancer (n = 34), breast cancer (n = 54), bile duct cancer (n = 25), ovarian cancer (n = 28), lung cancer (n = 95), colorectal cancer (n = 27), and duodenal cancer (n = 1). (**F**) ROC curves for the differentiation between patients with (n = 291) and without (n = 247) cancer using different metrics. (**G**) ROC curves for the differentiation between patients across different tumor stages using intra-/inter-nuc entropy ratio.

Minor comments:

7. Figure 4d is not convincing; claims of enrichment should be accompanied by appropriate statistical testing.

Response: We have included clearly labeled p-values for all statistical comparisons in all figures to enhance clarity in both main and supplementary figures.

8. Important methodological details, such as coverage, patient stage, and p-values, are missing throughout the text.

Response: We have incorporated detailed coverage data and patient stage information for each cohort, and provided p-values for all comparisons in the text and in figure caption.

Reviewer #2 (Remarks to the Author):

The authors present a Lorentzian model to decompose cfDNA fragment length distributions into multiple periodic components. By parameterizing each component with three variables: center, amplitude, and scale, the model identifies a transition point at ~159 bp that demarcates intra- from inter-nucleosomal cfDNA. The intra-/inter-nucleosomal amplitude ratio and scale ratio are proposed as new fragmentomic metrics, modestly distinguishing cancer-derived cfDNA from healthy samples (AUC ≈ 0.72). Overall, the work is mathematically elegant and offers a unifying parametric framework for cfDNA fragmentomics. However, the biological interpretation and quantitative validation of the model's superiority remain limited, and the translational significance is not yet fully established.
Response: We are deeply grateful to the reviewers for the constructive feedback. In response to the reviewer's comments, we have comprehensively and quantitatively validated the application of the Lorentzian model to cfDNA fragment length deconvolution and performed additional analyses to enhance the biological interpretation of nucleosomal architecture and ctDNA-specific fragmentation patterns revealed by size deconvolution.

major comments:
1. The introduction of the Lorentz distribution as a fitting function for cfDNA fragment size is conceptually appealing, providing an analytical representation of the periodic fragment peaks that reflect nucleosomal structure. However, the biological interpretation remains largely descriptive. The proposed Intra-/Inter-nucleosomal amplitude ratio essentially restates known fragmentomic features, the short/long fragment proportion under a new mathematical form. The 159 bp cutoff, while derived from model fitting, lacks independent biochemical validation (e.g., MNase-seq). Additional evidence such as enzymatic digestion experiments would be helpful to substantiate this demarcation as a genuine biological boundary rather than a mathematical guess.
Response: We thank the reviewer for the helpful feedback. We examined the fragment length distribution of Micrococcal Nuclease sequencing (MNase-seq) data from Chereji et al. (Genome Biol. 2019). However, as shown in Figure R8, the size profiles only showed weak 10-bp periodicity, likely due to the non-mammalian origin of micrococcal nuclease and the *in vitro* digestion environment. Therefore, instead of using MNase-seq data, we compared cfDNA prepared using a single-stranded library preparation kit (ssDNA). ssDNA exhibits unique properties in size profile, including a major peak at ~167 bp and minor peaks with ~3 bp offsets (Figure R4A). It therefore makes ssDNA an independent biochemical validation also under human cfDNA analysis framework. Please refer to our response to Reviewer #1 comment #4 response for ssDNA size deconvolution results.

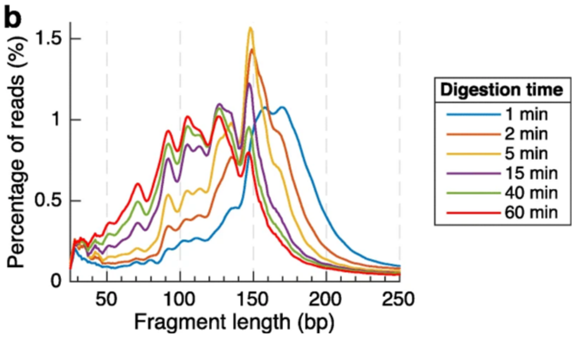

Figure. R7. Figure from Chereji et al. (*Genome Biol.* 2019). The length distributions of the mononucleosomal bands across increasing level of digestion.

2. The inclusion of multiple body fluids and datasets demonstrates model generalizability, yet the biological message is diluted by overemphasis on mathematical fitting. The manuscript would benefit from clearer discussion on what new underlying biological mechanistic insights the model provides.
Response: We thank the reviewer for the suggestion. Based on Reviewer #1 comment #5, we conceptually interpret the scale parameter as representing fragmentation entropy, since the entropy of a Lorentzian distribution is a function of its scale parameter. In addition, we summarize the amplitude parameter as reflecting protection strength against nuclease cleavage, which is conferred by the nucleosomal structure, in the discussion section.

We also noted Esfahani et al. demonstrated that Shannon entropy of cfDNA fragment length distribution in gene promoter regions correlates with gene expression. To clarify the advantages of component entropy over Shannon information entropy in quantification of cfDNA fragmentation entropy, we conducted a detailed comparison using cfDNA from Li-Fraumeni syndrome (LFS) patients. Please refer to our response to Reviewer #1 comment #5 response. This analysis is now included in the revised manuscript Figure 3, and we have revised the discussion section to more clearly convey the novel biological insights that size deconvolution analysis can provide.

3. If the main contribution of this paper is to establish a mathematically justified “fit-in-one” framework that better represents cfDNA size distributions across fluids, this claim requires quantitative validation. The authors should include quantitative model comparison metrics, such as adjusted R^2, residual plots comparing Lorentz vs. Gaussian or log-normal fits, etc. Demonstrating that the Lorentz model quantitatively outperforms Gaussian or exponential alternatives would transform this work from a visually convincing model to a quantitatively validated one.
Response: We thank the reviewer for the insightful comments. To provide solid quantitative validation of the Lorentzian-based fitting, we have included additional analyses comparing Lorentzian, Gaussian, and Student’s t-distribution models for cfDNA size deconvolution across five different bodily fluids, including a new lymphatic fluid cfDNA dataset. Given that the Student’s t-distribution with 1 degree of freedom (df = 1) corresponds to the Lorentzian distribution, and that it converges to the Gaussian distribution as df approaches infinity, the Student’s t-distribution with df = 5 provides a useful intermediate case with properties distinct from both the Lorentzian and Gaussian models. We used R^2 and residuals to reflect the degree of approximation. In all five bodily fluids, the Lorentzian model showed highest R^2 and lowest sum of absolute residuals, demonstrating the best fitting performance (Figure R2 and 3).

We also tested the log-normal distribution; however, due to its skewness (non-symmetric distribution), the lmfit Python module was not able to generate valid results (not shown). This also demonstrates that the components within the cfDNA size profile can only be represented by symmetric distributions. Please refer to our response to Reviewer #1 comment #1 for more explanation.

minor comments:
4. Prior studies would take 166bp as the modal cfDNA size of plasma, do you have any biological explanation for that using your hypothesis?
Response: We thank the reviewer for raising this important question. Both the 166 and 167 bp peak has been widely reported as the major peak in plasma cfDNA size distributions. In contrast, MNase-seq data identifies 147 bp as the core fragment length corresponding to DNA wrapped around histones (1 bp at the dyad plus 73 bp flanking each side) (Chereji Genome Biol., 2019); therefore, 167 bp was through to represent the DNA wrapping around core histones and an ~20 bp linker.

We hypothesized that the apparent shift in the overall size profile may result from the overlapping of the heavy tails of neighboring peaks. The 159-bp peak which is 8 bp left of 167 bp, may exert a stronger influence than the 177-bp peak (10 bp to the right). To test this, we simulated mixtures of two Lorentzian distributions with a major peak fixed at 167 bp and a minor peak sliding from 159 bp to 167 bp. As shown in Figure R9, we found that only peaks located more than or equal to 162 bp could alter the apparent peak of the summed curve, which is clearly far from 159 bp. Therefore, we conclude that the observed 166 bp modal size is not due to neighboring peaks but rather reflects a biologically meaningful feature that warrants further investigation. In our size deconvolution analysis, we allow the center parameter to be a floating-point number.

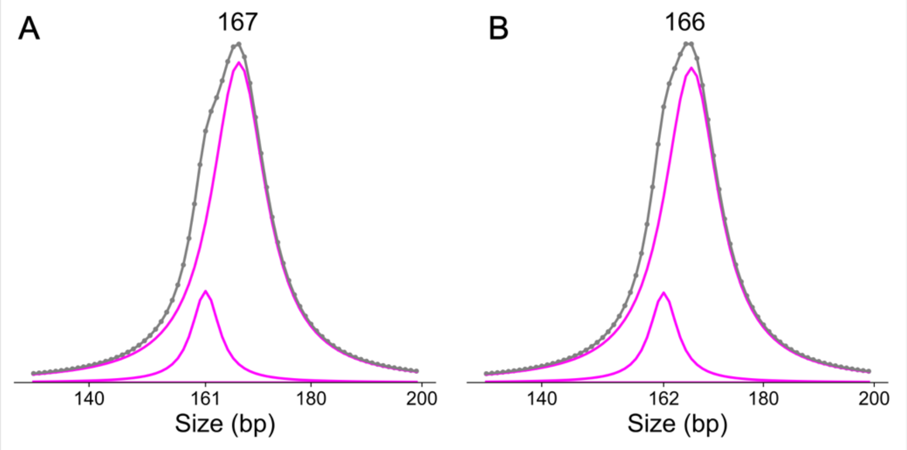

Figure R9. Simulation results of two Lorentzian distributions with locations at (**A**) 161 or (**B**) 162 bp, and 167 bp, scales of 3 bp and 6 bp, and amplitudes of 5% and 35%, respectively. The gray line indicates the sum of the two overlapping distributions, and the dots represent integer fragment sizes in base pairs.

5. line 167-170, please define the "higher and narrower than neighboring peaks", the sentence here is confusing.
Response: We have simplified the wording to “sharper than neighboring peaks” and clarified in the sentence.

6. Figure 3d, the intra-/inter- amplitude ratio in y-axis is from 2-8, corresponding to copy number 2-8. If I understand correctly, averaged intra- amplitude shall be the mean amplitude

from 81bp-152bp, averaged inter- amplitude shall be the mean amplitude from 159bp-221bp. The ratio shall not be as shown in y-axis.

Response: We thank the reviewer for the detailed evaluation of the figure. We have corrected the values and revised the figure to better demonstrate the fragmentation differences between cfDNA associated with linker and core histones.

7. line 342 shall be "combination" rather than "combiation"

Response: We thank the reviewer for thorough evaluation of our manuscript. We have corrected the typo.

We sincerely appreciate the reviewers' valuable criticism and constructive comments, which have guided us to refine our research objectives, reconsider the study design, and comprehensively revise and strengthen the manuscript. Our detailed, point-by-point responses to the comments are provided below.

RESPONSE TO REVIEWERS' COMMENTS

Reviewer #1 (Remarks to the Author):
In this manuscript, Zhou and colleagues present a mathematical framework for modeling cfDNA fragment length distributions using mixtures of Cauchy–Lorentz distributions. Applied to saliva, urine, CSF, and plasma samples, this model identifies periodic peaks spaced by approximately 10 bp, except for a distinct 159 bp peak whose amplitude is consistent across all fluid types. The authors propose that this peak reflects a boundary between intra- and inter-nucleosomal cfDNA fragments, which forms the basis for subsequent analyses. The model is later simplified (without sufficient methodological detail) to enable more robust fitting in additional sample types.
While this approach offers new perspectives, the work is limited in terms of novelty, validation, and demonstrated clinical utility. The absence of robust validation in larger cohorts and a lack of clear practical application lead the reviewer to not recommend publication in Nature Communications at this time.
We are deeply grateful for the reviewers' thoughtful comments. In accordance with the suggestions, we enhanced the logical coherence of the narrative, performed comprehensive validation and demonstrated markedly improved detection performance in two independent large multi-cancer cohorts.

Major comments:
1. The authors have overlooked a recent relevant study by the Velculescu Lab (van 't Erve et al., Nature Communications, 2024), which also utilizes mixtures of length distributions for tumor fraction estimation. The claimed superiority of the Cauchy over Gaussian models for cfDNA size density is not adequately substantiated and greatly limits the novelty of the work.
Response: We thank the Reviewer for highlighting this important work. van't Erve et al. utilize a random forest model to predict Mutant Allele Frequency (MAF) based on a total of 16 features. These include mixture model weights (11 features), chromosomal arm-level aneuploidy scores (PA-score), maximum absolute z-scores (2 features), and principal components of short-to-long fragment length ratios (2 features). The mixture model approach was described in more detail by Carey et al. in a 2021 poster (Carey et al. *Journal of Clinical Oncology*, 2021), where they approximated fragment length distributions using a mixture of 12 truncated normal distributions (Figure R1). Their work, utilizing modified Gaussian distributions, also clearly demonstrates that the Gaussian distribution is not able to properly model the cfDNA size profile.

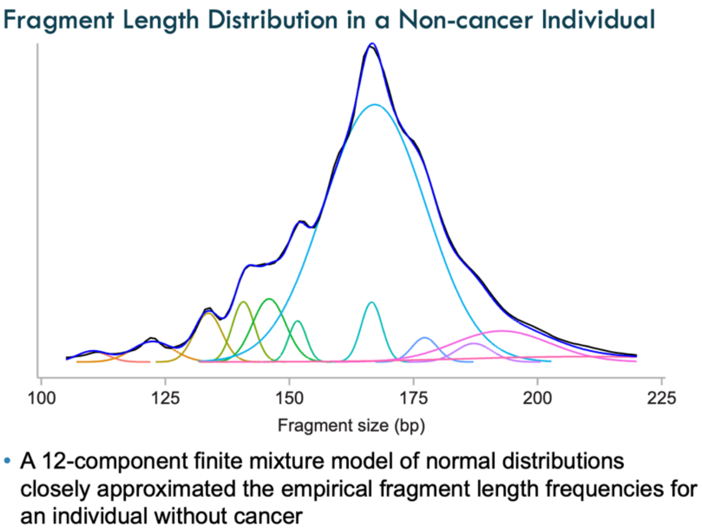

Figure R1. cfDNA size deconvolution using truncated Gaussian distributions (snapshot from Carey *et al*. Journal of Clinical Oncology 2021).

In the present work, our method employs the Cauchy–Lorentz distribution without any truncation or modification of the standard distribution, enabling us to more clearly capture cfDNA biology related to nucleosomal structures and tumor-specific fragmentation patterns. To prove superiority of the Cauchy over Gaussian models, also following Reviewer #2 comment #3, we have included additional analyses comparing Lorentzian, Gaussian, and Student's t-distribution models for cfDNA size deconvolution across five different bodily fluids. As shown in Figure R2, we applied cfDNA size deconvolution analysis using Lorentzian distribution, Gaussian distribution, and Student's t-distribution to cfDNA from saliva (Figure R2A), urine (Figure R2B), CSF (Figure R2C), lymphatic fluid (Figure R2D), and plasma (Figure R2E) samples. All models were run with the same parameter settings with same number of components. Both the Lorentzian (Figure R2 column 1) and Gaussian distributions (Figure R2 column 2) produced reliable fitting results, while the Student's t-distribution yielded unreliable fits. Its components exceeded the original size profiles and produced large residuals (Figure R2 column 3). The Lorentzian components yielded coefficients of determination (R^2; range: 0 to 1) approaching 1 in all five bodily fluids (all $R^2 > 0.99$), exceeding those of both the Gaussian and Student's *t* distributions (Figure R3 column 1). By evaluating size-banded fitting residuals (Figure R3 column 2) and the sum of absolute residuals (Figure R3 column 3), we demonstrate that the residuals from the Lorentzian model exhibited lower fluctuation strength and had lower sum of absolute residuals across all five bodily fluids compared to the Gaussian model. In particular, we included a new body fluid, lymphatic fluid (Figure R2D and 3D), in which the cfDNA length distribution forms a smoother curve without distinct sharp peaks. The Lorentzian model still showed the lowest sum of absolute residuals when modelling this distribution. Together, these results highlight the superiority of the Lorentzian distribution over the other models. We have included these result in Supplementary Fig. S1 and S2, and include van't Erve et al. and Carey et al. work in the introduction section.

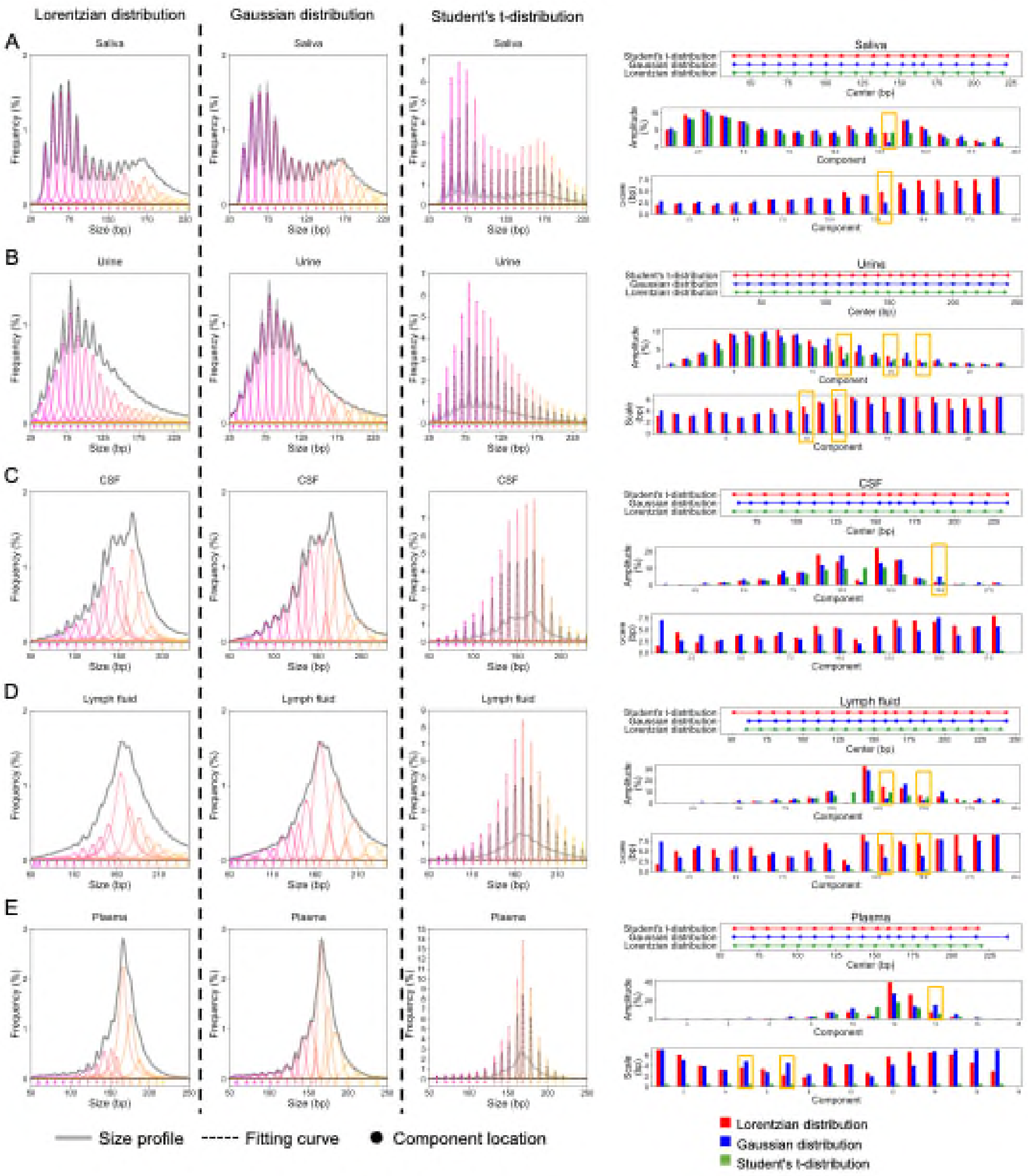

Figure R2. Comparison of fitting results from different mathematical models in cfDNA size deconvolution analysis. Size deconvolution analysis results of cfDNA from (**A**) saliva (27.1 million fragments from two samples; 19 peaks), (**B**) urine (149.9 million fragments from 18 samples; 22 peaks), (**C**) cerebrospinal fluid (CSF) (76.6 million fragments from 13 samples; 18 peaks), (**D**) lymphatic (lymph) fluid (906.6 million fragments from 28 samples; 18 peaks), and (**E**) plasma samples (497.1 million fragments from 19 samples; 17 peaks). Fitting results using the Lorentzian distribution are shown in the first column, Gaussian distribution in the second column, and Student's *t*-distribution (with degrees of freedom = 5) in the third column. All models use the same number of peaks, with each peak parameterized by three degrees of freedom (center, amplitude, and scale). Detailed information about component centers, amplitudes, and scales is shown in the fourth column, with irregular parameter values highlighted in yellow frames.

Bodily fluid	Distribution	R^2
Saliva	Lorentzian	**0.99628036**
	Gaussian	0.98037246
	Student's t	-2.58702270
Urine	Lorentzian	**0.99624745**
	Gaussian	0.99193350
	Student's t	0.24397542
CSF	Lorentzian	**0.99952503**
	Gaussian	0.99911664
	Student's t	0.26789946
Lymph fluid	Lorentzian	**0.99997845**
	Gaussian	0.99996673
	Student's t	0.18350547
Plasma	Lorentzian	**0.99978503**
	Gaussian	0.99973512
	Student's t	-0.53752270

A B C D E

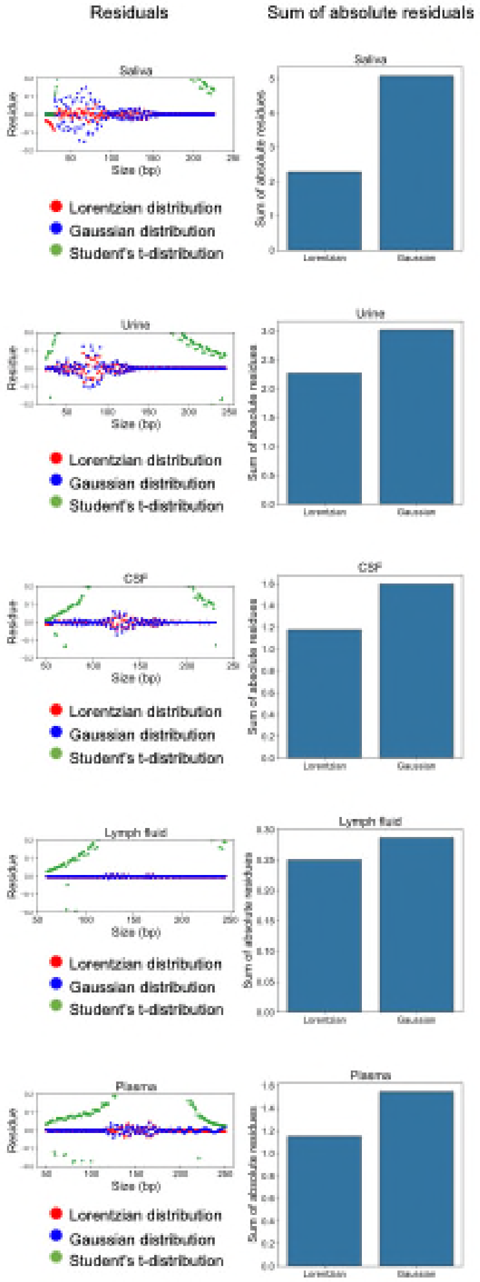

Figure R3: Comparison of fitting statistics from different mathematical models in cfDNA size deconvolution analysis. Size deconvolution analysis results of cfDNA from (**A**) saliva, (**B**) urine, (**C**) cerebrospinal fluid (CSF), (**D**) lymphatic (lymph) fluid, and (**E**) plasma samples using Lorentzian, Gaussian and student's *t* functions (same analyses as these of Supplementary Figure 1). R-squared, or the coefficient of determination (R^2) values, are shown in the first column table. An R^2 value of 1 indicates a perfect fit, and values closer to 1 indicate better model performance. The R^2 values closest to 1 is shown in bold for each bodily fluid analysis. The size-banded fitting residuals for each base pair (difference between the original size profile and the sum of deconvoluted components) are shown in the second column (range: -0.2 to 0.2). The sum of the absolute values of all residuals is shown in the third column.

2. The underlying data for Figure 1 are not described. Key details such as the number of samples, sequencing coverage, and other relevant parameters are missing, hindering interpretation and reproducibility.
Response: We thank the reviewer for the comment. We have now included more detail information on the number of samples and number of fragments for pooling samples; and added coverage statistics for all samples in all cohorts used in the size deconvolution analyses. The raw data of the size profiles from multiple bodily fluids (including additional lymphatic fluid cfDNA) presented in Figure 1 have also been added to Supplementary Table 1 to facilitate interpretation and ensure reproducibility. We also included a demonstration of size deconvolution analysis on both the Code Ocean website and GitHub (https://github.com/nrlab-CRUK/cfDNA_size_deconvolution).

3. The fitting procedure for Figure 1 needs clarification. How many degrees of freedom were employed, and were optimization constraints applied?
Response: We have now clarified the number of Lorentzian distributions applied in the deconvolution of cfDNA from different bodily fluids in Figure 1: (**C**) saliva (19 components), (**D**) urine (22 components), (**E**) CSF (18 components), (**F**) lymphatic fluid (18 components), and (**G**) plasma (17 components). Each component (peak) has three degrees of freedom (center, amplitude, and scale). In Figure 1, we pooled cfDNA from multiple bodily fluids to have smooth size profiles, e.g., saliva (27.1 million fragments), urine (149.9 million fragments), CSF (76.6 million fragments), lymphatic fluid (906.6 million fragments), and plasma samples (497.1 million fragments). For these high-depth samples, we did not apply any optimization constraints; we only restricted the maximum scale value below 9 bp and minimum scale value above 2 bp.

For sWGS data, we required those paired-scale intra-nucleosomal components have equal values, and restricting inter-nucleosomal components equal to or more than 167 bp to share the same scale value. In addition, guided by the Reviewer #1's suggestion #5, recognizing the importance of entropy, we applied L2 regularization on all component scales to prevent overfitting of fragmentation entropy.

4. The identification of the 159 bp peak appears arbitrary. Optimization solutions may not be globally optimal, and it is unclear whether 159 bp is genuinely superior to the widely adopted 150 bp threshold for "short" fragments, which has long been a standard in the cfDNA field [Snyder et al., Cell 2016; Jiang et al., PNAS 2015; Sun et al., PNAS 2015]. For the 159 bp component, the amplitude does not appear to carry more biologically, or clinically relevant information compared to previous work, but the scale parameter may be somewhat interesting; however, the reason for this is unclear and requires further justification. Overall, the section on "ctDNA exhibits increased amplitudes and scales of intra-nucleosomal components" does not seem to convey new insights given the existing literature, unless a specific novel aspect can be clarified.
Response: We thank the reviewer for the critical comment. We have added a new section titled "*159 bp serves as a demarcation between intra- and inter-nucleosomal cfDNA*" to the main text to clarify and reinforce the identification of the 159-bp peak as the boundary between intra- and inter-nucleosomal cfDNA. Also according to the Reviewer #2 comment #1, we now included a comparison between single-stranded cfDNA and conventional double-stranded cfDNA size profiles and further used the difference between ctDNA and cfDNA to demonstrate the biological significance of the 159-bp peak.

To determine whether the component at ~159 bp reflects a biologically meaningful nucleosomal structure or merely a mathematical artifact, we applied our method to plasma cfDNA prepared using single-stranded library protocol (ssDNA). Similar to double-stranded cfDNA (dsDNA), ssDNA exhibits a major peak at ~167 bp. Moreover, ssDNA has been reported to show ~3 bp offsets in the minor peaks of the fragment length distribution, which were also observed in Figure R4A. Our size deconvolution analysis provides a holistic view of fragment lengths for both dsDNA (Figure R4B; 16.9 million fragments) and ssDNA (Figure R4C; 27.9 million fragments) and allowed us to identify a previously unseen 159-bp component positioned between the minor (< ~150 bp) and major peaks (at ~167 bp), as well as additional components above the major peak at ~177 bp, ~188 bp, ~199 bp, and ~210 bp. In ssDNA (Figure R4C), we observed that all components below 159 bp exhibited a ~3 bp rightward offset compared to those of dsDNA (Figure R4B), suggesting a shared nucleosomal protection effect against enzymatic DNase digestion in short (< 159 bp) cfDNA fragments. By contrast, all components above 159 bp showed no obvious offset between ssDNA and dsDNA (Figure R4B and C), indicating a distinct fragmentation mechanism compared to that of short (< 159 bp) cfDNA. Together, these results suggest that the component centered at ~159 bp may be associated with biologically meaningful nucleosomal structures.

cfDNA fragments shorter than 150 bp, exhibiting a ~10 bp periodicity in size distributions, are thought to mainly originate from nucleosomal core particle. We hypothesized that the 159-bp DNA fragment might consist of 1 bp at the nucleosome dyad flanked by two 79 bp stretches of DNA (1+79+79 bp). As illustrated in the three-dimensional nucleosome structure (Figure R4D), both ends of the 79 bp segments flanking the dyad are tightly bound to linker histone H1. This aligns with our observation that the components below 159 bp show ~3 bp offsets between ssDNA and dsDNA, potentially reflecting protection conferred by nucleosome structures. Furthermore, the lower amplitude of the 159-bp component compared to the preceding component may indicate weaker protection strength conferred by linker histone H1 binding relative to core histones wrapping (Figure R4B and C).

To further explore this hypothesis, we analyzed ctDNA from a patient with stage IV breast cancer. As ctDNA is expected to be enriched in genomic regions showing tumor-associated copy number gains, we compared cfDNA molecules mapped to copy-number neutral regions (N = 2; 48.7 million fragments), regions with copy number gain (N = 3; 46.9 million fragments), amplification regions (N = 4; 7.3 million fragments), and high-level amplification regions (N = 5, 6, 7, and 8; fragments: 9.6, 3.5, 3.9, and 2.0 million, respectively). As shown in Figure R4E, cfDNA fragments derived from genomic regions with higher levels of tumor copy number gain, exhibit a progressive increase in short fragments, reflecting the increased ctDNA fraction. We next performed size deconvolution analysis on cfDNA from regions with varying copy numbers. Supporting our hypothesis (Figure R4F), components amplitudes showed opposing trends across the 159-bp boundary. With increasing tumor-associated copy number, the amplitudes of components at ~121 bp, ~131 bp, ~141 bp, and ~151 bp showed significant positive correlations (Pearson’s $r \geqslant 0.99$, $P < 0.001$), whereas the amplitudes of components at ~159 bp, ~167 bp, ~177 bp, and ~188 bp showed strong negative correlations (Pearson’s $r \leqslant -0.96$, $P < 0.001$), demonstrating altered fragmentation patterns in ctDNA shifting between intra- and inter-nucleosomal DNA. Together, these observations in ssDNA and ctDNA may reveal 159 bp as a previously unrecognized fragment length feature associated with the pivot point between intra- and inter-nucleosomal cfDNA fragments.

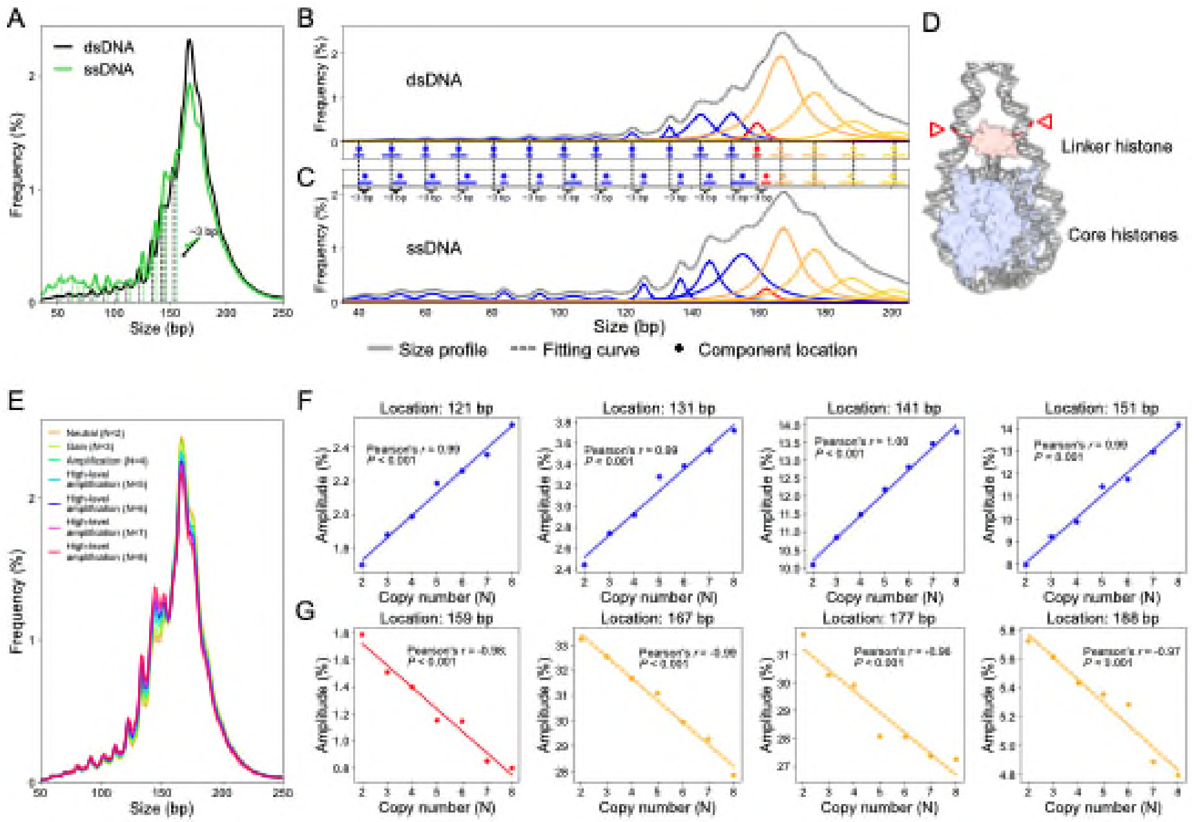

Figure R4. (**A**) Size profiles of double-stranded cfDNA (dsDNA; 16.9 million fragments) and single-stranded cfDNA (ssDNA; 27.9 million fragments). Size deconvolution analysis of (**B**) dsDNA and (**C**) ssDNA. (**D**) Illustration of a nucleosome with DNA wrapped around core histones (blue) and bound to linker histone H1 (red) (PDB: 5NL0). The red triangles indicate the two ends of a stretch of DNA spanning 159 nt, centered at the nucleosome dyad. (**E**) Size profiles of cfDNA molecules from different genomic regions, including tumor-associated copy number neutral (N = 2; 48.7 million fragments), gain (N = 3; 46.9 million fragments), amplification (N = 4; 7.3 million fragments), and high-level amplification regions (N = 5 to 8; 9.6, 3.5, 3.9, 2.0 million fragments, respectively). (**F, G**) Changes in amplitude parameters across tumor copy number status (N = 2 to 8) of Lorentzian components at ~121 bp to ~151 bp (blue), ~159 bp (red), and ~167 bp to ~188 bp (yellow)

5. The scale parameter of a Cauchy distribution proportionally increases its entropy. Previous work (Esfahani et al., Nat Biotech 2022) showed that entropy at gene promoters is correlated with expression. The reviewer suggests specifically testing whether intra-nucleosomal scale parameters at promoters correlate with expression—potentially more than mixed or inter-nucleosomal components—or determining if the intra-nucleosomal entropy (via the scale parameter) at promoters carries more information about the tumor compartment than the normal background.

Response: We are grateful to the reviewer for the insightful comment. Based on the reviewer's suggestion, we recognized that the entropy of a Lorentzian distribution is a function of its scale parameter, defined as the natural logarithm of the product of 4π and the scale, i.e., log(4πσ). Therefore, we conceptually interpret changes in the scale parameter as changes in cfDNA fragmentation entropy. Accordingly, we have refined the intra- to inter-nucleosomal scale ratio into an intra- to inter-nucleosomal entropy ratio in our analysis.

Based on the reviewer's interest in whether intra-nucleosomal entropy (via the scale parameter) at promoters correlates with gene expression, we obtained fragment length data from the original study (Esfahani et al., Nat. Biotechnol. 2022) [3]. As shown in Figure R5A, we first replicated the authors' analyses of cfDNA WGS fragment length heatmaps, gene expression profiles (GEP) of blood leukocytes assessed by RNA-seq, and promoter fragmentation entropy (PFE) distributions across 1,832 TSS of meta-gene, with each group encompassing 10 genes with similar expression levels. As shown in Figure R5B, we replicated the PFE developed by Esfahani. It showed a significant correlation with GEP (Pearson's $r = 0.90$, $P < 0.001$).

In total, we obtained 196.59 million fragments from 1 kb regions flanking these TSS groups (n = 1,832) and applied size deconvolution analysis to each TSS group, with an average of ~0.1 million fragments per group. As shown in Figure R5C, the entropy of the inter-nucleosomal components showed a significant correlation with gene expression levels assessed by RNA-seq (Pearson's $r = 0.74$, $P < 0.001$). Although Pearson's r value is slightly lower than the PFE metric, the inter-nucleosomal entropy nevertheless demonstrated feasibility for assessing cfDNA fragmentation entropy and showed potential for inferring gene expression. We argue that our Lorentzian distribution is more suitable for analyzing nucleosome-associated cfDNA, whereas promoter regions, where increased TF binding produces different signal, may require a more tailored algorithm (e.g., PFE) for gene expression inference.

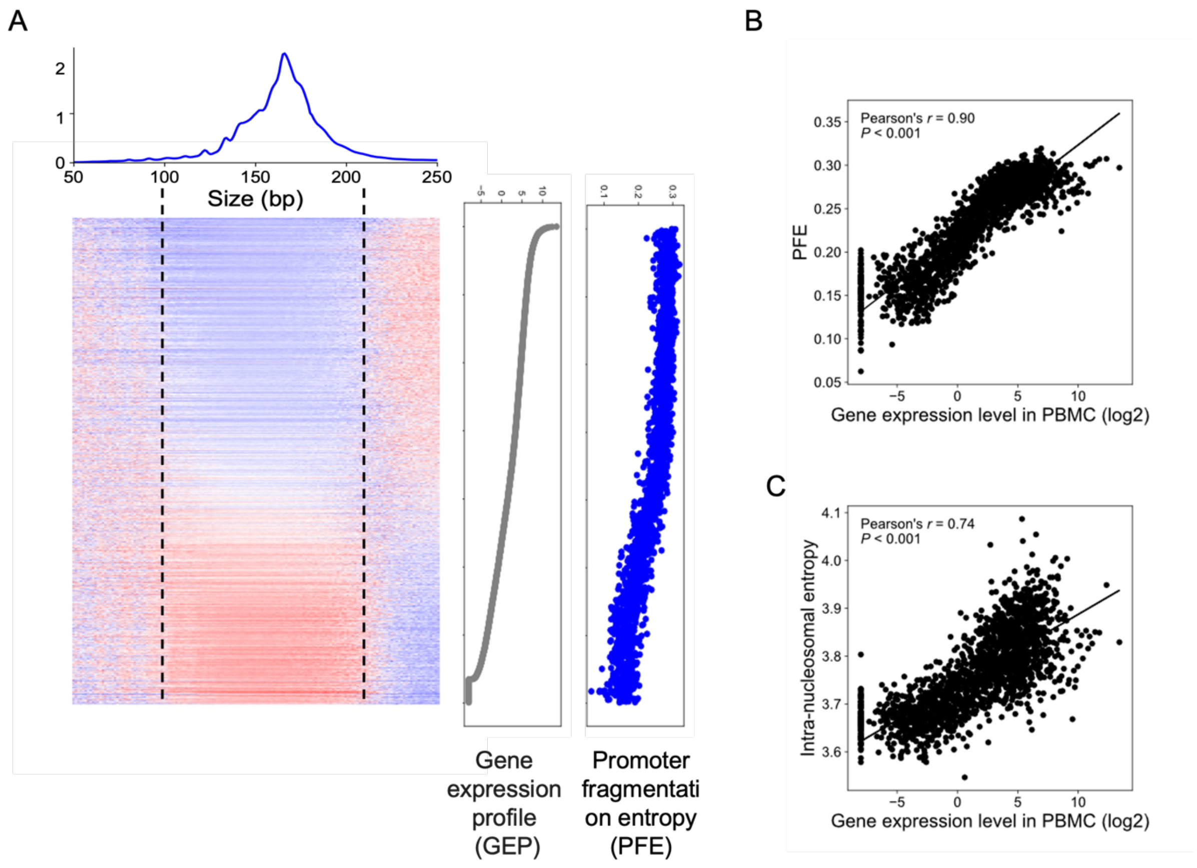

Figure R5. (**A**) Replicate analyses of plasma sample profile (blue line), heatmap capturing fragment density as ordered by gene expression profile (GEP) in blood leukocytes assessed by RNA-seq using TPM (grey line). Each row corresponds to one meta-gene encompassing the TSSs of ten genes when ranked by a reference PBMC expression vector. (**B**) Correlation

between gene expression profile (GEP) and promoter fragmentation entropy (PFE). (**C**) Correlation between GEP and inter-nucleosomal component entropy assessed by size deconvolution analysis.

We also included PFE in the analysis of the overall cfDNA fragment length distribution in Li-Fraumeni syndrome (LFS) patients. PFE showed no significant differences between healthy individuals and LFS patients with or without cancer in short fragments (Figure R6A; *P* = 0.08, Kruskal–Wallis test), long fragments (Figure R6B; *P* = 0.50, Kruskal–Wallis test), or their entropy ratio (Figure R6C; *P* = 0.20, Kruskal–Wallis test). However, intra-/inter-nucleosomal entropy ratio increase was considered as tumor specific fragmentation pattern and showed significant difference increase in LFS patients with cancer Figure R6D.

We reasoned that Shannon entropy measures the randomness of cfDNA fragment length distribution within a given range but does not account for the permutations of fragment lengths, which, however, can be captured by component parameters in the size deconvolution analysis. For example, two profiles with fragment abundances of 1–2–1–2–1 and 1–1–1–2–2 have identical Shannon entropy values, yet they would yield different peak parameters when analyzed by deconvolution.

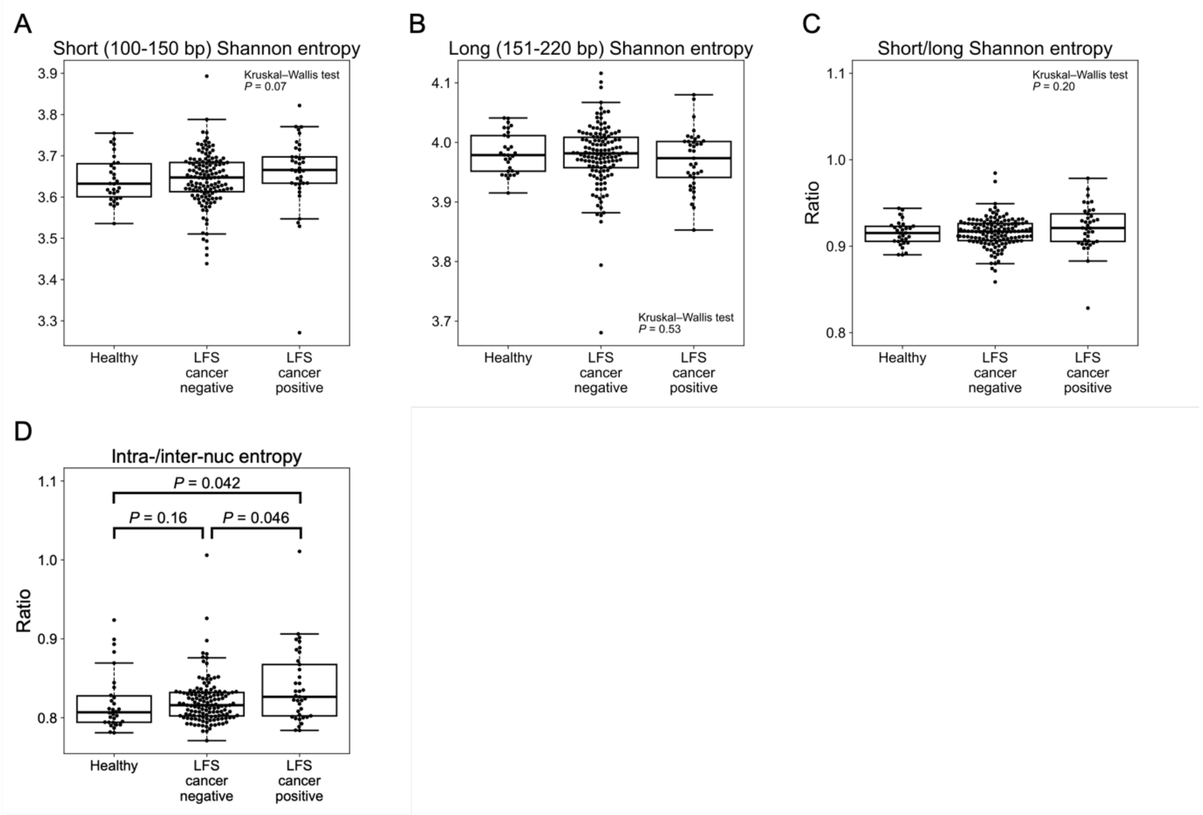

Figure R6: Boxplot of (**A**) short (100-150 bp) fragments Shannon entropy, (**B**) long (151-220 bp) fragments Shannon entropy and (**C**) their ratio in healthy individuals (n = 30), LFS patients without cancer (n = 131), and LFS patients with active cancer (n = 38). (**D**) Boxplot of intra-/inter-nucleosomal (intra-/inter-nuc) entropy ratios in healthy individuals (n = 30) and LFS patients with (n = 38) and without (n = 131) cancer.

6. The cancer detection section is poorly structured. Details regarding the classification model (type), featurization process (global versus bin-wise fitting similar to Cristiano et al., Nature 2019), and validation strategy (cross-validation versus label-free scoring) are unclear.

Additionally, the reported AUC (0.72) is unimpressive given that this cohort is predominantly late stage.
We thank the reviewer for the helpful comments. Based on the reviewer's suggestions, we have developed an intra-/inter-nucleosomal entropy ratio for cancer detection. To robustly apply size deconvolution analysis to sWGS, we developed an optimized size deconvolution algorithm. Briefly, we incorporate paired-scale constraints on intra-nucleosomal components and restrict all components above 167 bp to share the same scale value. Additionally, we apply L2 regularization across all component scales to prevent overfitting of fragmentation entropy.

We first obtained cfDNA fragment size profiles from healthy donors (n = 70) and patients with various late-stage cancer types (n = 280) from Mouliere et al. 2018, with a median coverage of 0.5x (range: 0.002 to 1.5x). We applied the size deconvolution analysis and calculated the intra-/inter-nucleosomal amplitude and entropy ratios for each individual. In addition, we examined the proportion of short fragments (<150 bp) and the short (100-150 bp) to long (151-220 bp) size ratio in overall plasma cfDNA size profile of each sample.

To objectively compare the ctDNA detection performance of these metrics, we did not apply machine learning algorithms and cross-validation to avoid overfitting and data leakage. Supporting our hypothesis, the intra-/inter-nucleosomal entropy ratio (Figure R7B) achieved the highest area under the ROC curve (AUC) value of 0.81 (Figure R7C) for differentiating patients with and without cancer, with 49% sensitivity at 95% specificity. The entropy ratio demonstrates significant superior performance over conventional fragment-length-based methods (short-to-long ratio: AUC = 0.63; $P < 0.001$, DeLong test; 31% sensitivity at 95% specificity; short fragment proportion: AUC = 0.65; $P < 0.001$, DeLong test; 33% sensitivity at 95% specificity; and intra-/inter-nucleosomal amplitude ratio: AUC = 0.66; $P < 0.001$, DeLong test; 33% sensitivity at 95% specificity).

Furthermore, we analyzed an additional independent cohort from the DELFI study (Cristiano et al. *Nature* 2019), consisting of 247 plasma cfDNA samples from healthy donors and 291 samples from cancer patients across eight cancer types (stage I: n = 45; stage II: n = 112; stage III: n = 35; stage IV: n = 81; and unknown stage: n = 3), with median coverage of 2.3x (range: 1.0 to 12.8x). As shown in Figure R7F, the intra-/inter-nucleosomal entropy ratio also achieved the highest AUC of 0.85 with 60% sensitivity at 95% specificity, significantly outperforming other metrics (short-to-long ratio: AUC = 0.67; $P < 0.001$, DeLong test; 33% sensitivity at 95% specificity; short fragment proportion: AUC = 0.67; $P < 0.001$, DeLong test; 33% sensitivity at 95% specificity; and intra-/inter-nucleosomal amplitude ratio AUC = 0.70; $P < 0.001$, DeLong test; 38 % sensitivity at 95% specificity).

We further analyzed the performance of the intra-/inter-nucleosomal entropy ratio for cancer detection across different tumor stages. As shown in Figure R7G, it achieved sensitivities of 51%, 59%, 60%, and 68% at 95% specificity (AUCs of 0.83, 0.82, 0.86 and 0.91) for identifying cancer patients at stages I, II, III, and IV, respectively, demonstrating potential in early-stage cancer detection. The superior performance of the intra-/inter-nucleosomal entropy ratio in the two large cohorts (totaling 888 samples) demonstrates that cfDNA size deconvolution analysis can enhance cancer detection by targeting ctDNA-specific fragmentation patterns.

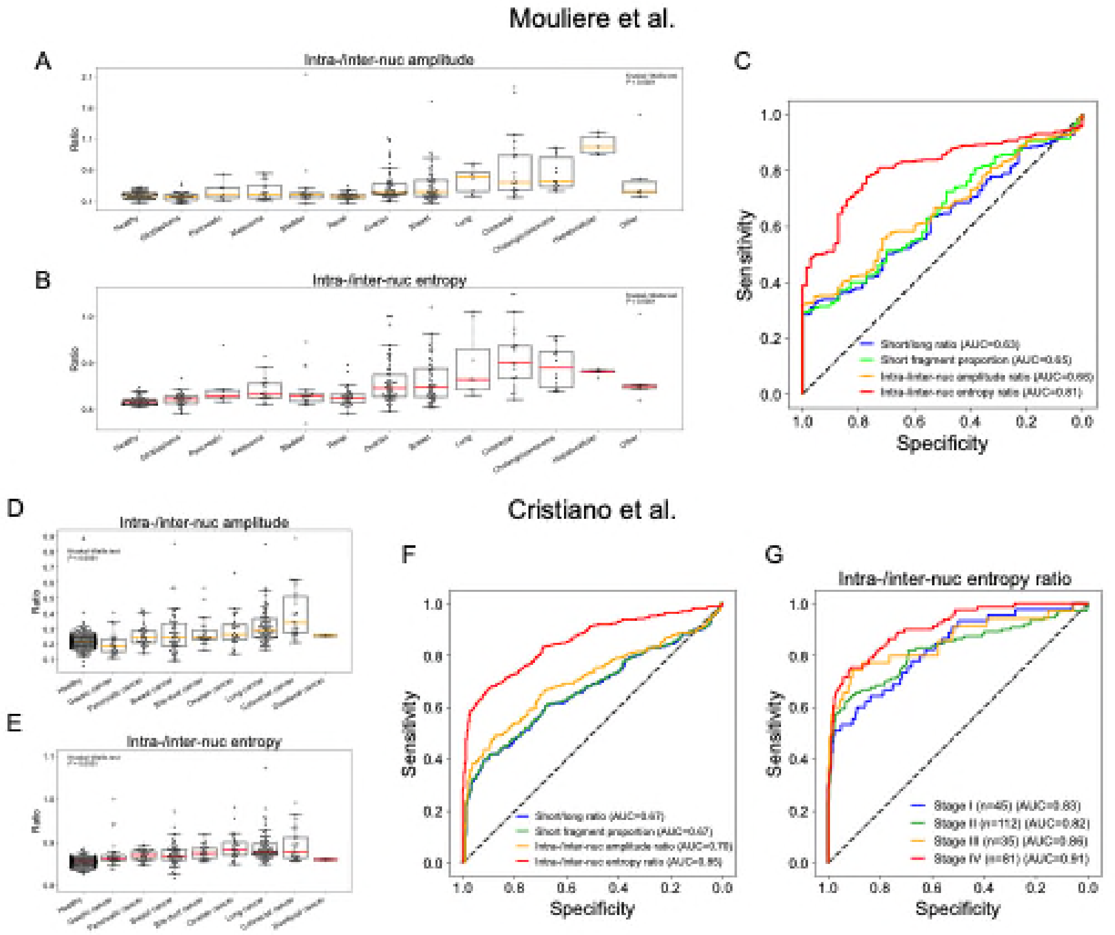

Figure R7. Boxplots of intra-/inter-nuc (**A**) amplitude ratios, and (**B**) entropy ratios in plasma cfDNA of 70 healthy controls and 280 patients with various cancer types, including glioblastoma (n = 34), pancreatic cancer (n = 7), melanoma (n = 21), bladder cancer (n = 19), renal cancer (n = 33), ovarian cancer (n = 59), breast cancer (n = 53), lung cancer (n = 8), colorectal cancer (n = 21), cholangiocarcinoma (n = 14), hepatocellular carcinoma (n = 5), and other cancer types (n = 6). (**C**) ROC curves for the differentiation between patients with (n = 280) and without (n = 70) cancer using different metrics. Boxplots of (**D**) intra-/inter-nuc amplitude ratios and (**E**) entropy ratios in plasma cfDNA of 247 healthy individuals and 291 patients with various cancer types, including gastric cancer (n = 27), pancreatic cancer (n = 34), breast cancer (n = 54), bile duct cancer (n = 25), ovarian cancer (n = 28), lung cancer (n = 95), colorectal cancer (n = 27), and duodenal cancer (n = 1). (**F**) ROC curves for the differentiation between patients with (n = 291) and without (n = 247) cancer using different metrics. (**G**) ROC curves for the differentiation between patients across different tumor stages using intra-/inter-nuc entropy ratio.

Minor comments:
7. Figure 4d is not convincing; claims of enrichment should be accompanied by appropriate statistical testing.
Response: We have included clearly labeled p-values for all statistical comparisons in all figures to enhance clarity in both main and supplementary figures.

8. Important methodological details, such as coverage, patient stage, and p-values, are missing throughout the text.

Response: We have incorporated detailed coverage data and patient stage information for each cohort, and provided p-values for all comparisons in the text and in figure caption.

Reviewer #2 (Remarks to the Author):

The authors present a Lorentzian model to decompose cfDNA fragment length distributions into multiple periodic components. By parameterizing each component with three variables: center, amplitude, and scale, the model identifies a transition point at ~159 bp that demarcates intra- from inter-nucleosomal cfDNA. The intra-/inter-nucleosomal amplitude ratio and scale ratio are proposed as new fragmentomic metrics, modestly distinguishing cancer-derived cfDNA from healthy samples (AUC ≈ 0.72). Overall, the work is mathematically elegant and offers a unifying parametric framework for cfDNA fragmentomics. However, the biological interpretation and quantitative validation of the model's superiority remain limited, and the translational significance is not yet fully established.
Response: We are deeply grateful to the reviewers for the constructive feedback. In response to the reviewer's comments, we have comprehensively and quantitatively validated the application of the Lorentzian model to cfDNA fragment length deconvolution and performed additional analyses to enhance the biological interpretation of nucleosomal architecture and ctDNA-specific fragmentation patterns revealed by size deconvolution.

major comments:
1. The introduction of the Lorentz distribution as a fitting function for cfDNA fragment size is conceptually appealing, providing an analytical representation of the periodic fragment peaks that reflect nucleosomal structure. However, the biological interpretation remains largely descriptive. The proposed Intra-/Inter-nucleosomal amplitude ratio essentially restates known fragmentomic features, the short/long fragment proportion under a new mathematical form. The 159 bp cutoff, while derived from model fitting, lacks independent biochemical validation (e.g., MNase-seq). Additional evidence such as enzymatic digestion experiments would be helpful to substantiate this demarcation as a genuine biological boundary rather than a mathematical guess.
Response: We thank the reviewer for the helpful feedback. We examined the fragment length distribution of Micrococcal Nuclease sequencing (MNase-seq) data from Chereji et al. (Genome Biol. 2019). However, as shown in Figure R8, the size profiles only showed weak 10-bp periodicity, likely due to the non-mammalian origin of micrococcal nuclease and the *in vitro* digestion environment. Therefore, instead of using MNase-seq data, we compared cfDNA prepared using a single-stranded library preparation kit (ssDNA). ssDNA exhibits unique properties in size profile, including a major peak at ~167 bp and minor peaks with ~3 bp offsets (Figure R4A). It therefore makes ssDNA an independent biochemical validation also under human cfDNA analysis framework. Please refer to our response to Reviewer #1 comment #4 response for ssDNA size deconvolution results.

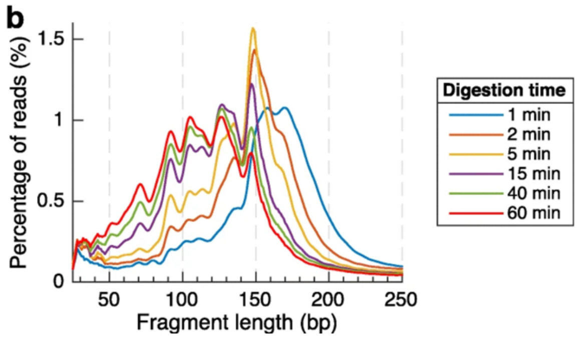

Figure. R7. Figure from Chereji et al. (*Genome Biol.* 2019). The length distributions of the mononucleosomal bands across increasing level of digestion.

2. The inclusion of multiple body fluids and datasets demonstrates model generalizability, yet the biological message is diluted by overemphasis on mathematical fitting. The manuscript would benefit from clearer discussion on what new underlying biological mechanistic insights the model provides.
Response: We thank the reviewer for the suggestion. Based on Reviewer #1 comment #5, we conceptually interpret the scale parameter as representing fragmentation entropy, since the entropy of a Lorentzian distribution is a function of its scale parameter. In addition, we summarize the amplitude parameter as reflecting protection strength against nuclease cleavage, which is conferred by the nucleosomal structure, in the discussion section.

We also noted Esfahani et al. demonstrated that Shannon entropy of cfDNA fragment length distribution in gene promoter regions correlates with gene expression. To clarify the advantages of component entropy over Shannon information entropy in quantification of cfDNA fragmentation entropy, we conducted a detailed comparison using cfDNA from Li-Fraumeni syndrome (LFS) patients. Please refer to our response to Reviewer #1 comment #5 response. This analysis is now included in the revised manuscript Figure 3, and we have revised the discussion section to more clearly convey the novel biological insights that size deconvolution analysis can provide.

3. If the main contribution of this paper is to establish a mathematically justified "fit-in-one" framework that better represents cfDNA size distributions across fluids, this claim requires quantitative validation. The authors should include quantitative model comparison metrics, such as adjusted R^2, residual plots comparing Lorentz vs. Gaussian or log-normal fits, etc. Demonstrating that the Lorentz model quantitatively outperforms Gaussian or exponential alternatives would transform this work from a visually convincing model to a quantitatively validated one.
Response: We thank the reviewer for the insightful comments. To provide solid quantitative validation of the Lorentzian-based fitting, we have included additional analyses comparing Lorentzian, Gaussian, and Student's t-distribution models for cfDNA size deconvolution across five different bodily fluids, including a new lymphatic fluid cfDNA dataset. Given that the Student's t-distribution with 1 degree of freedom (df = 1) corresponds to the Lorentzian distribution, and that it converges to the Gaussian distribution as df approaches infinity, the Student's t-distribution with df = 5 provides a useful intermediate case with properties distinct from both the Lorentzian and Gaussian models. We used R^2 and residuals to reflect the degree of approximation. In all five bodily fluids, the Lorentzian model showed highest R^2 and lowest sum of absolute residuals, demonstrating the best fitting performance (Figure R2 and 3).

We also tested the log-normal distribution; however, due to its skewness (non-symmetric distribution), the lmfit Python module was not able to generate valid results (not shown). This also demonstrates that the components within the cfDNA size profile can only be represented by symmetric distributions. Please refer to our response to Reviewer #1 comment #1 for more explanation.

minor comments:
4. Prior studies would take 166bp as the modal cfDNA size of plasma, do you have any biological explanation for that using your hypothesis?
Response: We thank the reviewer for raising this important question. Both the 166 and 167 bp peak has been widely reported as the major peak in plasma cfDNA size distributions. In contrast, MNase-seq data identifies 147 bp as the core fragment length corresponding to DNA wrapped around histones (1 bp at the dyad plus 73 bp flanking each side) (Chereji Genome Biol., 2019); therefore, 167 bp was through to represent the DNA wrapping around core histones and an ~20 bp linker.

We hypothesized that the apparent shift in the overall size profile may result from the overlapping of the heavy tails of neighboring peaks. The 159-bp peak which is 8 bp left of 167 bp, may exert a stronger influence than the 177-bp peak (10 bp to the right). To test this, we simulated mixtures of two Lorentzian distributions with a major peak fixed at 167 bp and a minor peak sliding from 159 bp to 167 bp. As shown in Figure R9, we found that only peaks located more than or equal to 162 bp could alter the apparent peak of the summed curve, which is clearly far from 159 bp. Therefore, we conclude that the observed 166 bp modal size is not due to neighboring peaks but rather reflects a biologically meaningful feature that warrants further investigation. In our size deconvolution analysis, we allow the center parameter to be a floating-point number.

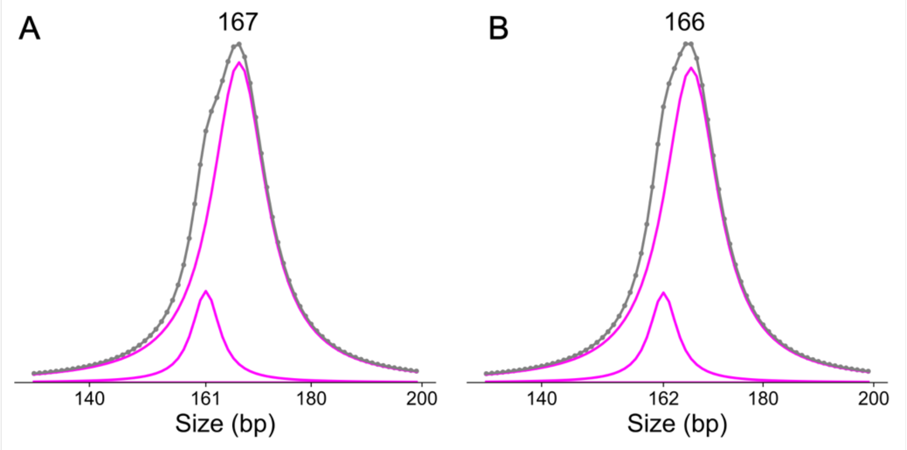

Figure R9. Simulation results of two Lorentzian distributions with locations at (**A**) 161 or (**B**) 162 bp, and 167 bp, scales of 3 bp and 6 bp, and amplitudes of 5% and 35%, respectively. The gray line indicates the sum of the two overlapping distributions, and the dots represent integer fragment sizes in base pairs.

5. line 167-170, please define the "higher and narrower than neighboring peaks", the sentence here is confusing.
Response: We have simplified the wording to “sharper than neighboring peaks” and clarified in the sentence.

6. Figure 3d, the intra-/inter- amplitude ratio in y-axis is from 2-8, corresponding to copy number 2-8. If I understand correctly, averaged intra- amplitude shall be the mean amplitude

from 81bp-152bp, averaged inter- amplitude shall be the mean amplitude from 159bp-221bp. The ratio shall not be as shown in y-axis.
Response: We thank the reviewer for the detailed evaluation of the figure. We have corrected the values and revised the figure to better demonstrate the fragmentation differences between cfDNA associated with linker and core histones.

7. line 342 shall be "combination" rather than "combiation"
Response: We thank the reviewer for thorough evaluation of our manuscript. We have corrected the typo.

Reviewer #1 (Remarks on code availability):

The authors have addressed my comments thoroughly. However, several results still do not convincingly demonstrate that the more complex mathematical model yields meaningful new biological insight or a clear translational advantage. For example, Figure R3 is underwhelming: the Lorentzian model appears only marginally better than the Gaussian model.

Response: We thank the reviewer for the careful evaluation of Figure R3 and the detailed feedback. We agree that the R^2 values of the Lorentzian model are only marginally higher than those of the Gaussian model.

However, beyond the R^2 and residual values shown in Figure R3, the Lorentzian model demonstrates progressively changing amplitude and scale values across all components. In contrast, the Gaussian model produces several outlier amplitude and scale values (highlighted in yellow frames in the right bar plots of Figure R2). These outliers indicate instability in the parameter estimates and suggest that the Gaussian distribution is less suitable for modelling cfDNA size profiles.

In addition to quantitatively validating the suitability of the Lorentzian distribution for approximating cfDNA size profiles, we also identified two mathematical properties that further support its compatibility with modelling fragment length distributions.

First, a random variable following a Lorentzian distribution (e.g., fragment length distribution) can be expressed as the ratio of two Gaussian-distributed variables. Because cfDNA molecules arise from the interplay between nuclease-mediated DNA cleavage and protection by DNA-binding proteins, this mathematical property may reflect the underlying biological processes involved in cfDNA generation.

Second, the Lorentzian distribution is a stable distribution, meaning that a linear combination of two independent Lorentzian-distributed variables also follows a Lorentzian distribution. Since cfDNA represents a mixture of DNA fragments released from multiple tissues, the observation that the overall cfDNA size profile follows a Lorentzian distribution suggests that cfDNA fragments originating from individual tissues may also exhibit Lorentzian-like distributions.

Reviewer #2 (Remarks to the Author):

Thanks for the rebuttal and additional analyses. Overall, the revisions addressed my prior concerns. The authors now provide clearer methodological details and a more coherent biological interpretation. I think it's good to go.

Response: We thank the reviewer for the positive feedback and for recognizing the improvements made in revision.

Reviewer #2 (Remarks on code availability):

I'm not familiar with coding. I focused more on wet lab.

Response: We appreciate the reviewer’s comment. We have made the code publicly available in GitHub and provided clear documentation and example workflows to ensure that it can be easily understood and used by others.
